# Herbal Medicine for Colorectal Cancer Treatment: Molecular Mechanisms and Clinical Applications

**DOI:** 10.1111/cpr.70065

**Published:** 2025-06-09

**Authors:** Zuqing Su, Yanlin Li, Zihao Zhou, Bing Feng, Haiming Chen, Guangjuan Zheng

**Affiliations:** ^1^ State Key Laboratory of Traditional Chinese Medicine Syndrome The Second Affiliated Hospital of Guangzhou University of Chinese Medicine Guangzhou China; ^2^ Guangdong Provincial Key Laboratory of Chinese Medicine for Prevention and Treatment of Refractory Chronic Diseases The Second Affiliated Hospital of Guangzhou University of Chinese Medicine Guangzhou China; ^3^ Guangdong‐Hong Kong‐Macau Joint Lab on Chinese Medicine and Immune Disease Research The Second Affiliated Hospital of Guangzhou University of Chinese Medicine Guangzhou China; ^4^ State Key Laboratory of Dampness Syndrome of Chinese Medicine The Second Affiliated Hospital of Guangzhou University of Chinese Medicine Guangzhou China

**Keywords:** colorectal cancer, herbal medicine, molecular mechanisms

## Abstract

Colorectal cancer (CRC) is one of the most common malignant tumours and is the second leading cause of cancer‐related mortality worldwide. Despite the availability of preventative, diagnostic and treatment methods including endoscopic treatment, surgical intervention, radiotherapy, biologics, salvage therapy and immunotherapy, the mortality rate associated with CRC remains alarming. Consequently, there is a pressing need to search for medicines for the treatment of CRC. Phytomedicines have been shown to suppress the proliferation and metastasis of CRC through various mechanisms, including immune regulation, modulation of gut microbiota, targeting of stem cells, macrophage polarisation, glycolysis, ferroptosis induction, modulation of extracellular vesicles, activation of mitochondria‐induced apoptosis, inflammation reduction, oxidative stress management and intervention of autophagy. Furthermore, numerous studies have reported the anti‐cancer and anti‐metastatic effects of various phytomedicines, including curcumin, resveratrol, berberine, shikonin, dihydroartemisinin, fucoidan, luteolin, andrographolide, piperine, kaempferol, emodin, cannabidiol, tanshinone IIA and evodiamine. In this review, we sort out the effects and mechanisms of phytomedicines on CRC and outline the major phytomedicines commonly used in CRC treatment. We hope that these phytomedicines may serve as promising drugs or important lead compounds for the management of CRC.

## Graphical abstract

Colorectal cancer is a prevalent gastrointestinal tumour and the second deadliest cancer worldwide, affecting approximately 10% of all cancers and cancer‐related deaths. Current clinical methods for treating CRC cause gastrointestinal abnormalities and myelosuppression. Phytomedicines inhibit CRC through immune regulation, gut microbiota, stem cells, macrophage, glycolysis, ferroptosis, mitochondrial functions and so forth.

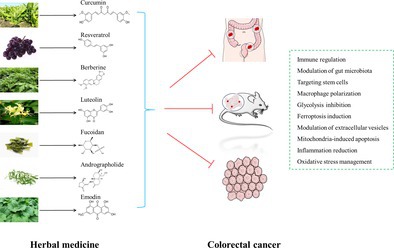

**Abbreviations**
5‐HT3: 5‐hydroxytryptamine receptor subtype 3
ABCG2: ATP binding cassette subfamily G member 2
ACF: aberrant crypt foci
ADCY4: adenylate cyclase 4
AIF: apoptosis‐inducing factor
ALOX12B: arachidonate 12‐lipoxygenase, 12R type
AOM: azoxymethane
Apaf‐1: apoptotic protease activating factor‐1
ARRDC4: arrestin domain containing 4
CAC: colitis‐associated colorectal cancer
CCL2: C–C motif chemokine ligand 2
CI: confidence interval
CRC: colorectal cancer
CSCs: cancer stem cells
CXB: celecoxib
CXCR5: C–X–C motif chemokine receptor 5
DCs: dendritic cells
DEPDC5: dishevelled, Egl‐10 and pleckstrin domain‐containing protein 5
DSS: dextran sulfate sodium
DT‐13: saponin monomer 13 of the dwarf lilyturf tuber
EMT: epithelial–mesenchymal transition
FAK: focal adhesion kinase
FLIM: fluorescence lifetime imaging microscopy
GFP: green fluorescent protein
GLUT1: glucose transporter type 1
GPR81: G protein‐coupled receptor 81
GRh3: ginsenoside Rh3
GRP78: glucose‐regulated protein 78 kD
GSDMD: gasdermin D
HIF‐1α: hypoxia‐inducible factor 1 subunit alpha
HPA: hypothalamic–pituitary–adrenal
ICIs: immune checkpoint inhibitors
ITGBL1: integrin subunit beta like 1
JAK2: Janus kinase 2
JNK‐Mff: c‐Jun N‐terminal kinase—Mitochondrial fission factor
JPJDR: JianPi JieDu Recipe
KLT: Kanglaite
LDHA: lactate dehydrogenase A
LNT: lentinan
LPS: lipopolysaccharide
MDSCs: myeloid‐derived suppressor cells
MMP: matrix metalloproteinases
MRP2: multidrug resistance proteins
MSD: the modified Shenlingbaizhu decoction
NLRP3: NOD‐, LRR‐ and pyrin domain‐containing protein 3
NRF2: nuclear factor erythroid 2‐related factor 2
PAD4: protein‐arginine deiminase type‐4
PD‐1: programmed cell death protein 1
PKM2: pyruvate kinase M2
Poly E: polyphenon E
PRDX2: peroxiredoxin 2
ROS: reactive oxygen species
SLC7A11: suppressing solute carrier family 7 member 11
SP: side population
STAT4: signal transducer and activator of transcription 4
TAZ: transcriptional co‐activator with PDZ‐binding motif
TCM: Traditional Chinese Medicine
TER: triterpenoids
TIMP: tissue inhibitor of metalloprotease
TLR4: Toll‐like receptor 4
TNFAIP3: TNF‐alpha‐induced protein 3
TOX2: TOX high mobility group box family member 2
Tregs: regulatory T cells
TXYF: Tong‐Xie‐Yao‐Fang
WMW: Wumei Wan
XIAP: X‐linked inhibitor of apoptosis protein
YAP1: Yes‐associated protein 1
YYFZBJS: Yi‐Yi‐Fu‐Zi‐Bai‐Jiang‐San
ZO‐1: Zonula occludens‐1

## 1 Introduction

Colorectal cancer (CRC) is a prevalent gastrointestinal tumour and the second deadliest cancer worldwide [1]. Recent epidemiological data show that poor dietary habits and a sedentary lifestyle, characterised by insufficient physical activity, have contributed to a year‐on‐year increase in the incidence rate of CRC, posing a significant threat to human health [2]. The incidence of CRC has declined in developed countries due to the ongoing promotion of CRC screening and advancements in screening technologies. While the 5‐year survival rate for stage I CRC is 90%, this rate decreases to 72% for locally advanced CRC and drops to a mere 14% for patients with metastatic CRC [3]. Current clinical methods for treating CRC include surgical intervention, radiotherapy, biologics and immunotherapy. However, these approaches have various limitations. For example, endoscopic treatment is primarily suitable for early‐stage tumours (non‐metastatic), while radiation therapy can induce side effects such as gastrointestinal abnormalities and myelosuppression. Additionally, there is a limited population suitable for targeted therapy, and off‐target effects can occur [4, 5]. Recently, the role of phytomedicines against CRC, whether as primary or adjunctive therapies, has received increasing attention. Research indicates that phytomedicines affect CRC through immune regulation (CD4^+^/CD8^+^ T lymphocytes, dendritic cells, Th17 cells and regulatory T cells), gut microbiota, colorectal cancer stem cells, macrophage polarisation, glycolysis, ferroptosis, extracellular vesicles, mitochondrial function, inflammation, oxidative stress and autophagy. Therefore, this review aims to outline the efficacy and molecular mechanisms of phytomedicines against CRC and summarise promising drug candidates, with the goal of identifying precursor drugs for future drug development.

## 2 Cell Proliferation

### 2.1 Immune Regulation

#### 2.1.1 CD4 ^+^/CD8 ^+^ T Lymphocytes

Currently, cancer immunotherapy represents one of the most innovative options for the treatment of CRC [6]. It has evolved from a basic adjuvant therapy into one of the most effective treatment modalities available. Compared to traditional therapies, immunotherapy overcomes the problems of radiotherapy and chemotherapy by regulating the patient's own immune system [7]. CD4^+^ and CD8^+^ T cells are crucial components of the cellular immune response. CD4^+^ T cells are important for regulating infections and are critical for the development of memory CD8^+^ T cells after stimulation [8, 9]. Insufficient CD4^+^ and CD8^+^ T cells can cause rapid growth and metastasis of tumours. Conversely, enhancing the proportion of these cells within the tumour immune microenvironment can significantly improve the anti‐cancer effects [10].

**FIGURE 1:**
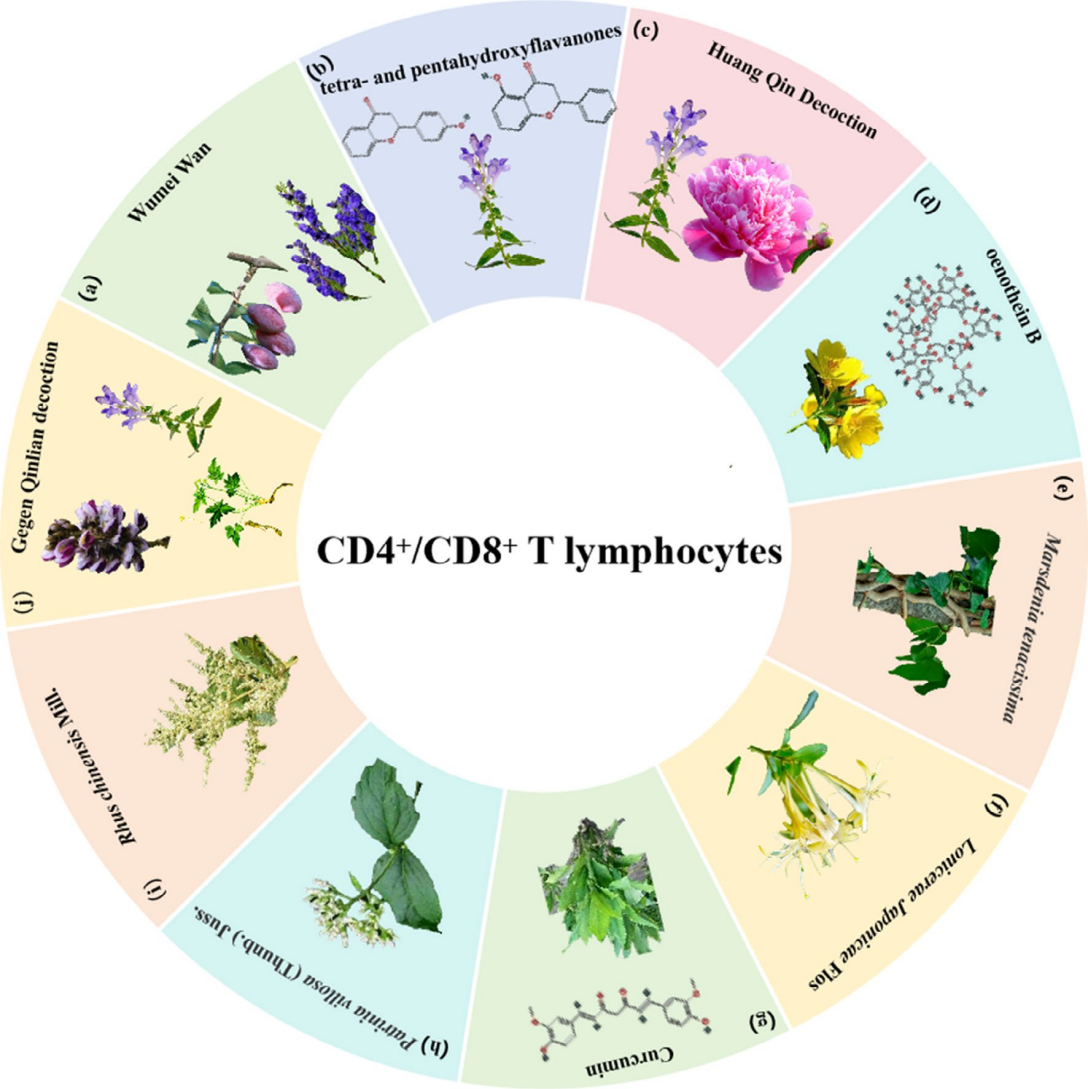
Herbal medicine treats CRC by regulating CD4^+^/CD8^+^ T lymphocytes. (a) *Prunus mume* Siebold & Zucc. and *Aconitum carmichaelii* Debeaux. (b) *Scutellaria baicalensis* Georgi. (c) *Scutellaria baicalensis* Georgi and *Paeonia lactiflora* Pall. (d) *Oenothera biennis* L. (e) *Marsdenia tenacissima* (Roxb.) Wight et Arn. (f) *Lonicerae Japonicae* Flos. (g) *Curcuma longa* L. (h) *Patrinia villosa* (Thunb.) Juss. (i) *Rhus chinensis* Mill. (j) *Pueraria montana* (Lour.) Merr., *Scutellaria baicalensis* Georgi and *Coptis chinensis* Franch.

Wumei Wan (WMW) is a traditional Chinese medicine (TCM) prescription. In a male C57BL/6J mouse model of colitis‐associated colorectal cancer (CAC) induced by azoxymethane (AOM) and dextran sulfate sodium (DSS), WMW effectively suppresses colonic inflammation and tumour growth during the early CAC. Additionally, WMW inhibits myeloid‐derived suppressor cells (MDSCs) and enhances CD4^+^ and CD8^+^ T‐cell populations in the spleen. Transcriptomic and serum metabolomic analyses indicate that amino acid metabolism and the PI3K/AKT signalling pathway are critical for WMW to exert an inhibitory effect on early‐stage CAC [10]. However, the specific metabolites that ultimately influence the regulation of CD4^+^ and CD8^+^ T‐cell function through the modulation of MDSCs and their mechanisms of action have not been addressed. In an AOM/DSS‐induced CAC mouse model, Kimura and Sumiyoshi et al. explore the therapeutic effects of tetra‐ and pentahydroxyflavanones isolated from *Scutellariae* radix on CRC. They found that tetra‐ and pentahydroxyflavanones decreased the tumour volume by 60.6% and 72.9%, respectively, in the colons. Additionally, these compounds suppressed IL‐10 and programmed cell death protein 1 (PD‐1) expressions by inhibiting cyclooxygenase‐2 (COX‐2) and TOX/TOX2 expressions, which could transcriptionally and epigenetically induce CD8^+^ T‐cell exhaustion. Therefore, tetra‐ and pentahydroxyflavanones inhibit AOM/DSS‐induced CRC by downregulating COX‐2 and TOX/TOX2 expressions, thereby decreasing IL‐10 and PD‐1 levels in tumour tissues [11]. Additional research is required to elucidate the impact of tetra‐ and pentahydroxyflavanones on the recovery from CD8^+^ T‐cell exhaustion both in vivo and in vitro. Huang Qin Decoction (HQD), a traditional Chinese medicine, is utilised for the treatment of CRC. In an AOM/DSS‐induced CAC mouse model, Pan et al. demonstrated that HQD significantly suppressed the incidence of colon cancers, alleviated intestinal inflammation and diminished neutrophil infiltration in the colon. Additionally, HQD restored intestinal mucosal permeability by enhancing intestinal tight junction protein (occludin and ZO‐1) expressions and improved the immunosurveillance capacity of CD8^+^ T cells. Further analyses using network pharmacology, immunohistochemistry and immunoblotting indicated that HQD interfered with the initiation of CAC by modulating PAD4‐dependent neutrophil extracellular traps [12]. HQD has been recognised for its efficacy in the management of gastrointestinal cancers. This article illustrates that HQD mitigates tumour‐associated inflammation via the modulation of neutrophils; however, further investigation into the precise mechanisms underlying this effect is warranted. Immune checkpoint inhibitors (ICIs) represent a significant advancement in cancer immunotherapy, in which ICIs intercept immune checkpoint proteins on immune cells, thereby preventing immune cells from recognising and killing cancer cells. By inhibiting these checkpoints, ICIs enable the immune system to effectively recognise and target cancer cells [13]. PD‐1 is an important immune checkpoint protein primarily expressed on the surface of T lymphocytes, including CD8^+^ T cells. When the PD‐1 protein on CD8^+^ T cells binds to PD‐L1 on cancer cells, it signals the immune system to stop targeting and eliminating those cancer cells [14]. Evening primrose ( *Oenothera biennis* ) is used globally for the treatment of inflammatory and metabolic diseases. Lee et al. found that evening primrose root extract effectively blocked the molecular interaction between PD‐L1 and PD‐1, thereby increasing CD8^+^ T‐cell‐mediated tumour cytotoxicity and subsequently reducing tumour growth. Additionally, oenothein B (an active compound derived from evening primrose) and FOLFOX (5‐fluorouracil plus oxaliplatin) exhibited a synergistic effect in inhibiting hPD‐L1‐MC38 cell proliferation in an ex vivo model by activating CD8^+^ tumour‐infiltrating T lymphocytes [14]. *Marsdenia tenacissima* (Roxb.) Wight et Arn. is a widely used Chinese herbal medicine. Yi et al. found that *Marsdenia tenacissima* tablets effectively elevated CD3^+^/CD8^+^ tumour‐infiltrating T cells in 13 out of 17 CRC patients (76.5%). Additionally, *Marsdenia tenacissima* tablets suppressed TGF‐β1 and PD‐L1 expressions in the CRC cell lines HCT116 and LoVo while enhancing the density of CD3^+^/CD8^+^ T cells and inhibiting tumour growth in CAC mouse models [15]. *Rhus chinensis* Mill., a medicinal plant belonging to the Rhus genus of the sumac family, is frequently utilised in the treatment of cancer in China, India and Japan. Wang et al. found that the triterpenoids of *Rhus chinensis* prevented CD8^+^ T‐cell dysfunction and promoted glycolytic activation in these cells, which is beneficial for T cells in recognising and eliminating tumour cells [16]. Honeysuckle (*Lonicerae Japonicae* Flos) is a TCM with potent anti‐inflammatory, antioxidant and anti‐tumour properties. In a CT26 tumour‐bearing C57BL/6 mouse model, both *Sidt1* ^+/+^ and *Sidt1* ^*−*/*−*^ mouse models, honeysuckle‐derived microRNA2911 slowed CRC by promoting CD4^+^ and CD8^+^ T‐cell infiltration and down‐regulating the expression of TGF‐β1. As anticipated, the therapeutic effects of microRNA2911 were cancelled in T‐cell‐deficient mice, suggesting that microRNA2911 depends on functionally normal T cells to exert its anti‐CRC effects [17]. It is widely recognised that honeysuckle contains a diverse array of microRNAs, including miR160, miR162, miR166, miR171, miR2914 and miR2910. The potential anti‐tumour properties of these microRNAs merit further investigation. Additionally, the prospect of a synergistic effect among them in combating CRC appears to be highly promising. Yang et al. found that *Patrinia villosa* aqueous extract exhibited significant anti‐proliferative and anti‐metastatic effects by regulating the TGF‐β R1‐smad2/3‐E‐cadherin and FAK‐RhoA‐cofilin pathways. Additionally, it increased helper T cells (CD3^+^CD4^+^) and cytotoxic T lymphocytes (CD3^+^CD8^+^) percentages in spleen and thymus tissues [18]. Turmeric is the root of *Curcuma longa* L. and is used as a dietary supplement and a therapeutic drug for metabolic disorders and cancer [19]. Li et al. found that turmeric extract, which contains absorbable curcumin, inhibited tumour cell growth and liver and lung metastasis in a xenograft model by targeting cofilin, FAK/pSrc, AKT, ERK and STAT3. Furthermore, it increased CD4^+^ helper T lymphocytes in the thymus and CD8^+^ cytotoxic T lymphocytes in tumour tissue [20]. Furthermore, the researchers also validated the reduction in immune function following FOLFOX treatment in a murine model. However, they found that turmeric extract enhanced the immune capacity of mice, indicating that turmeric extract is worth further research in conjunction with FOLFOX treatment. In a mouse xenograft model of CT26 tumours, Gegen Qinlian decoction, a TCM, combined with anti‐mouse PD‐1 significantly increased the proportion of CD8^+^ T cells in both peripheral blood and tumour tissues [21]. In conclusion, herbal medicines may be a promising strategy to significantly enhance anti‐PD‐1‐mediated immunotherapy for CRC patients by modulating the percentages of CD4^+^ and CD8^+^ T lymphocytes (Figure 1).

#### 2.1.2 Dendritic Cells

Dendritic cells (DCs) derive their name from the numerous dendrites that extend from their cell membranes, resembling the structure of nerve cells. These cells hold significant potential for clinical applications in tumour treatment due to their unique anti‐tumour responses and their role in regulating T‐cell activity [22, 23]. While DCs do not directly attack CRC cells, they function as antigen‐presenting cells that capture information from these cancer cells. This information is processed within the DCs, enabling them to effectively communicate their characteristics to T cells [24]. The ability of DCs to present antigens is crucial for the initiation of both innate and adaptive immunity. By targeting the potential influence of DCs on the tumour microenvironment in CRC, a novel approach to immunotherapy for this disease appears increasingly viable [25, 26].

**FIGURE 2:**
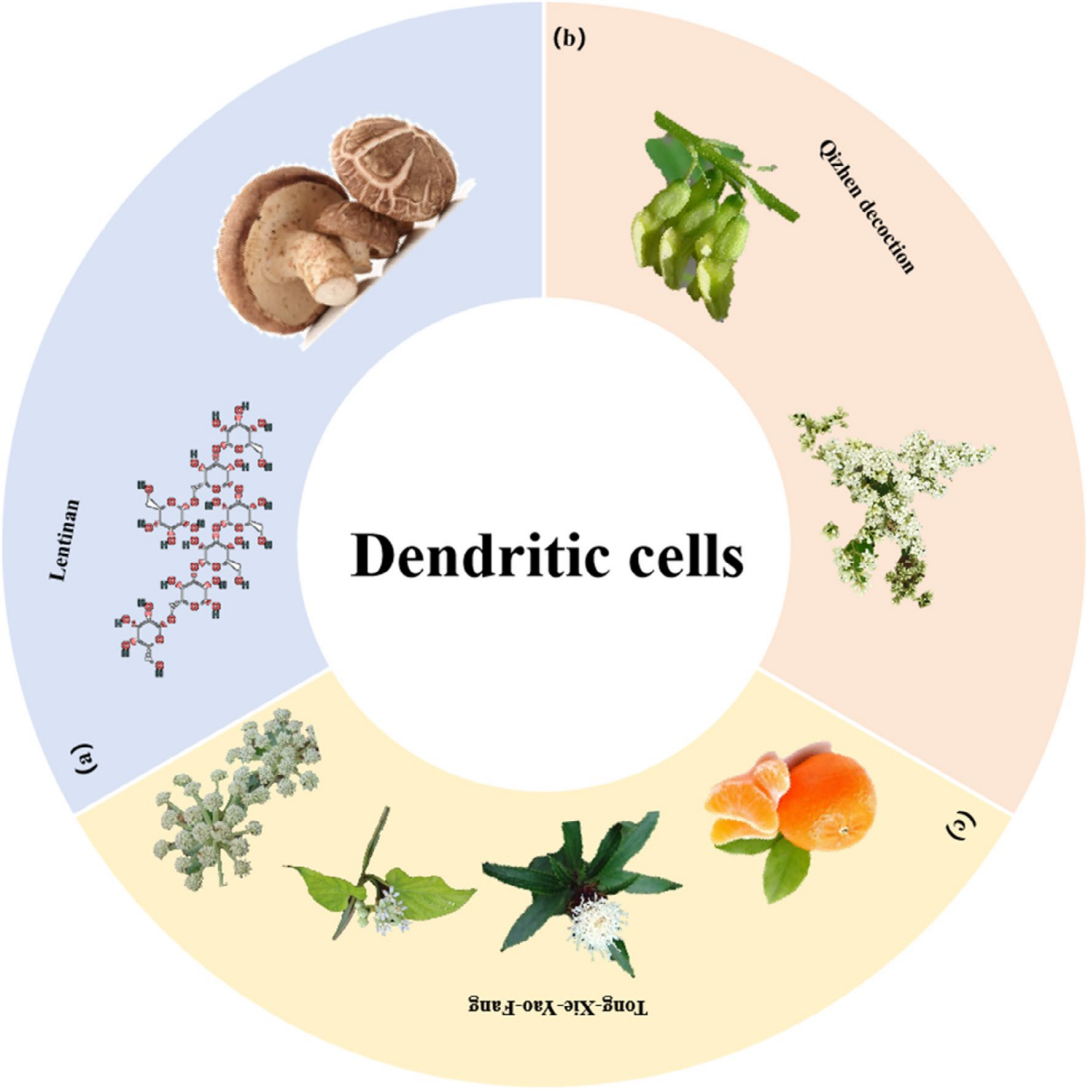
Herbal medicine treats CRC by regulating dendritic cells. (a) *Lentinus edodes* (Berk.) Sing. (b) *Astragalus membranaceus* (Fisch.) Bunge and *Ligustrum lucidum* Ait. (c) *Citrus reticulata* Blanco, *Atractylodes macrocephala* Koidz., *Cynanchum otophyllum* Schneid. and *Saposhnikovia divaricata* (Turcz.) Schischk.

Lentinan (LNT) is a water‐soluble polysaccharide extracted from *Lentinus edodes* (Berk.) sing., known for its potent immune‐enhancing properties. Mao et al. found that LNT could promote the maturation of DCs and polarise tumour‐associated macrophages to the anti‐tumorigenic M1 phenotype (CD11b^+^CD80^+^) in the CT26 CRC tumour model [27]. Furthermore, Huang and colleagues found that LNT suppressed tumour angiogenesis through interferon γ and in a T‐cell‐independent manner in the CT26 CRC tumour model [28]. Qizhen decoction is a traditional Chinese herbal formula used to treat CRC with significant efficacy [29]. In an AOM/DSS‐induced CRC mouse model, QZD combined with a PD‐1 inhibitor (InVivoMab) promoted DCs maturation, consequently releasing IL‐12 and activating the JAK2/STAT4 pathway, which induced effector T‐cell activation by increasing the abundance of *Akkermansia* [30]. Tong‐Xie‐Yao‐Fang (TXYF) is a classical formula commonly used to treat intestinal disorders in China. TXYF was shown to suppress tumour growth in CRC mice with chronic restraint stress by inhibiting the hypothalamic–pituitary–adrenal axis and facilitating DCs maturation. This response was evidenced by an increase in the percentages of CD4^+^ T cells, CD4^+^/CD8^+^ T cells and Th1 cells [31]. The findings indicate that DCs may serve as a crucial intermediary in the exacerbation of CRC associated with depression. Consequently, targeting DCs could represent a novel approach in the clinical management of CRC (Figure 2).

#### 2.1.3 Th17 Cells

**FIGURE 3:**
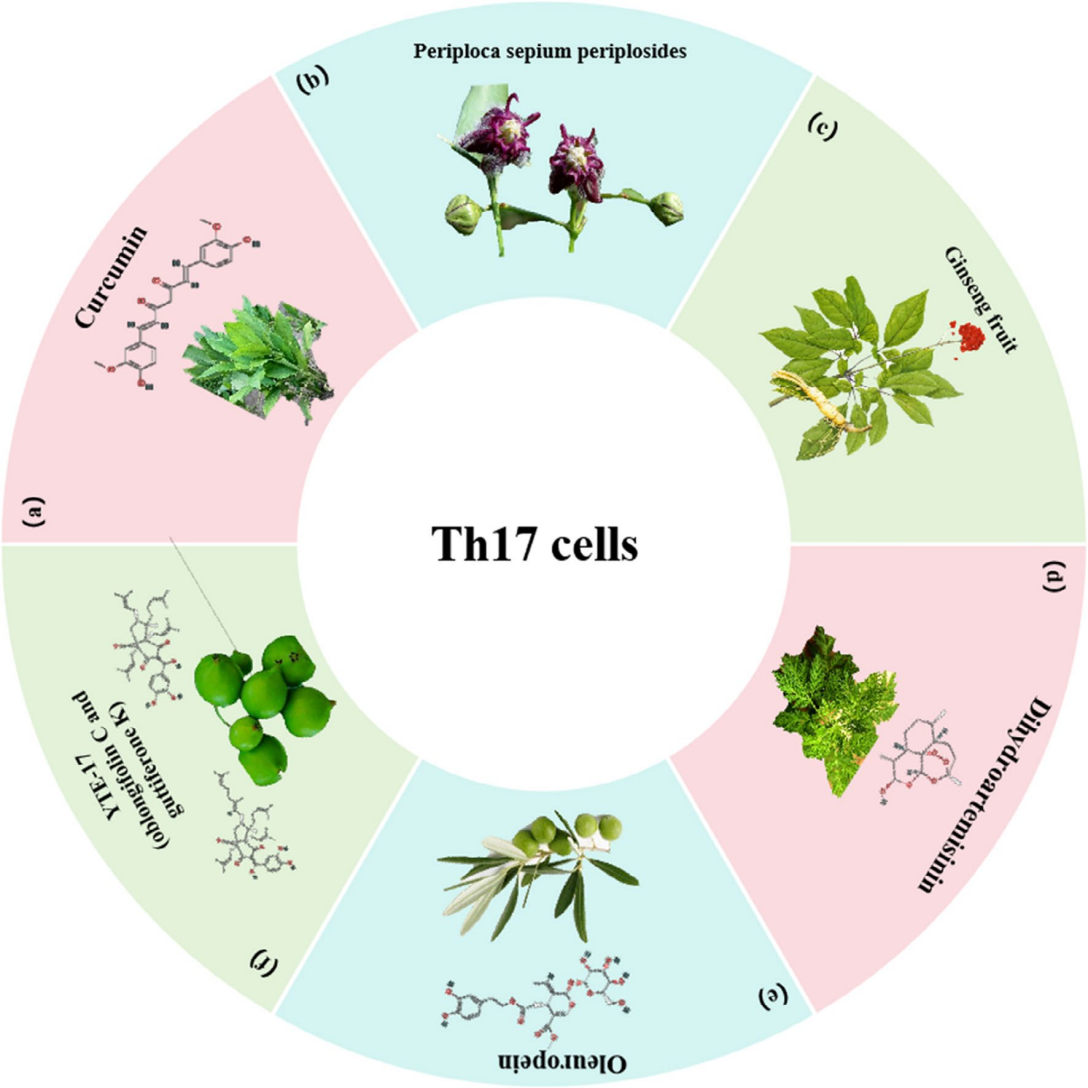
Herbal medicine treats CRC by regulating Th17 cells. (a) *Curcuma longa* L. (b) *Periploca sepium* Bunge. (c) *Panax ginseng* C.A. Meyer. (d) *Artemisia caruifolia Buch*.‐Ham. ex Roxb. (e) *Olea europaea* L. (f) *Garcinia yunnanensis* H. H. Hu.

Th17 cells are a subset of CD4^+^ T cells that secrete interleukin‐17 and are considered protumorigenic immune cells in CRC. *Curcumae Longae* Rhizoma, the root of *Curcuma longa* L., has been used as a dietary supplement for thousands of years and is commonly employed to treat metabolic disorders and cancer [32]. The main components of *Curcumae Longae* Rhizoma are curcuminoids, with curcumin being the active ingredient. Wan et al. found that a novel surfactant‐formulated curcumin inhibited SW480 cell proliferation by promoting cyclin B1‐induced early apoptosis and inducing cell cycle arrest. Additionally, it suppressed Th17 cell differentiation to alleviate intestinal inflammation [32]. *Periploca sepium* periplosides, extracted from the dried root of *Periploca sepium* Bunge (*P*. *sepium*), are used to treat rheumatoid arthritis, dyspepsia and stomachache [33]. Lin et al. discovered that *Periploca sepium* periplosides improved the structure of gut microbiota, thereby suppressing colitis and CAC by inhibiting the pathogenic Th17 cell population in both the DSS‐induced acute colitis model and the DSS + AOM‐induced CAC model (C57BL/6) [33]. This research possesses substantial scholarly value, and it is anticipated that future investigations will explore in greater depth the specific types of gut microbiota that affect the regulation of Th17 cells, along with the underlying mechanisms governing this regulatory process. Ginseng fruit ( *Panax ginseng* C.A. Meyer), which encompasses both berries and seeds, is recognised for its immune‐enhancing properties. Wang et al. found that while ginseng berry concentrate did not affect Th1 and Treg cell differentiation, it significantly inhibited Th17 cell differentiation, thereby regulating the Th17/Treg balance in adaptive immunity [34]. Future research is anticipated to investigate this mechanism using CRC animal models. Beyond that, there are numerous chemical compounds found in plants that have been shown to inhibit CRC by inhibiting Th17 cells, including dihydroartemisinin [35], oleuropein [36] and YTE‐17 (oblongifolin C and guttiferone K) [37] (Figure 3).

#### 2.1.4 Regulatory T Cells

Regulatory T cells (Tregs) are a subset of CD4^+^ T cells characterised by their low proliferation capacity and immunosuppressive effects. Considering that they break the immune responses of Th1, Th2 and Th17 cells through the secretion of IL‐10 and TGF‐β1, Tregs are believed to contribute to CRC evasion of immune surveillance [38, 39, 40].

**FIGURE 4:**
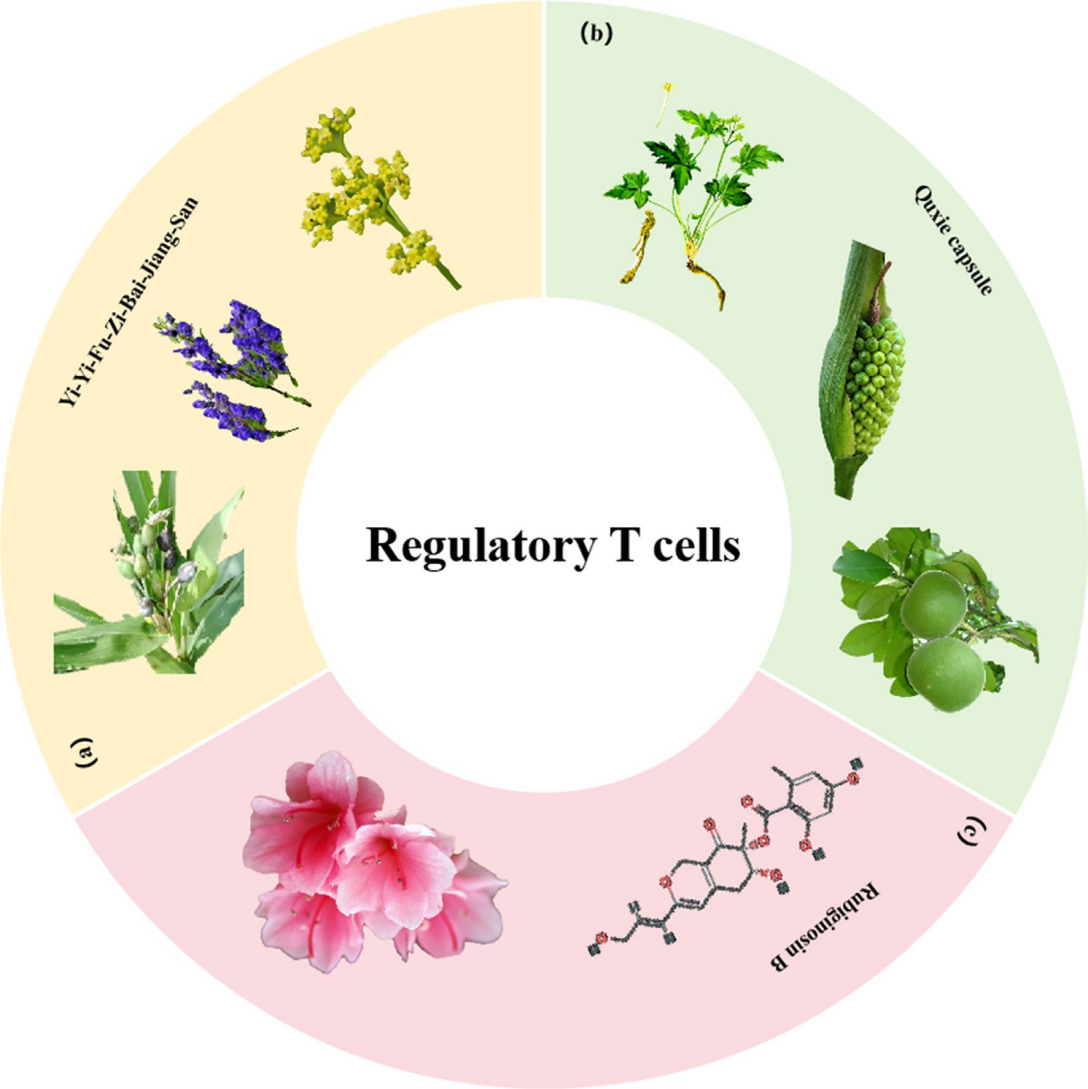
Herbal medicine treats CRC by regulating regulatory T cells. (a) *Coix lacryma*‐*jobi* , *Aconitum carmichaelii* Debeaux and *Patrinia scabiosifolia* Fisch. ex Trevir. (b) *Coptis chinensis* Franch., *Pinellia ternata* (Thunb.) Ten. ex Breitenb. and *Citrus grandis* ‘Tomentosa’. (c) *Rhododendron brachypodum* Fang & P. S. Liu.

Yi‐Yi‐Fu‐Zi‐Bai‐Jiang‐San (YYFZBJS) is a commonly used herbal formulation for the treatment of gastrointestinal disorders in China. A study involving C57BL/6J *Apc* ^*Min*/+^ mice demonstrated that YYFZBJS effectively regulated gut flora, including *Bacteroides fragilis* and *Lachnospiraceae*. Furthermore, when faeces from YYFZBJS‐treated human volunteers were administered to both conventional and germ‐free mice, a significant inhibition of intestinal tumour growth was observed. Additional research indicated that the underlying mechanisms are associated with the regulation of gut microbiota, inhibition of CD4^+^CD25^+^Foxp3^+^ Tregs and a reduction in intestinal inflammation [41]. However, Zhang et al. further demonstrated that YYFZBJS suppressed tumour growth by promoting CD4^+^CD25^+^ Tregs‐induced immunosuppression by targeting HIF‐1α‐induced hypoxia in MC‐38 cells and in AOM/DSS‐induced CRC mice [42]. The aforementioned research findings prompt consideration of whether HIF‐1α serves as a primary target for the regulation of Tregs by intestinal microbiota. Additionally, it is essential to explore the mediators through which intestinal flora exerts this regulatory effect, including inflammatory metabolites and short‐chain fatty acids. Quxie capsule is an herbal remedy used for the treatment of advanced CRC for over years. Chen et al. found that the Quxie capsule elevated the ratios of Th17/Tregs while inhibiting Foxp3 expression, an important moderator of Tregs, thereby suppressing colorectal tumorigenesis in CRC mice and in HCT26 and HCT116 cells [43]. Nonetheless, there have been sporadic instances of patients experiencing abdominal discomfort or diarrhoea potentially attributable to Quxie capsule, as it contains certain herbal components, such as *Crotonis* fructus, known to stimulate bowel activity [43]. It is important to note that these adverse effects are generally mild and manageable, and CRC patients often express a willingness to persist with Quxie capsule treatment. Rubiginosin B is obtained from *Rhododendron brachypodum* (R. brachypodum). Geng et al. found that rubiginosin B selectively suppressed TGFβ‐induced CD4^+^Foxp3^+^ Tregs cell differentiation, thereby enhancing the ability of the immune system to monitor and kill tumour cells by regulating the calcineurin‐NFAT pathway in the CT26 and MC38 xenograft tumour models [44] (Figure 4).

### 2.2 Gut Microbiota

There is a consensus that dysbiosis of the gut microbiota is positively associated with CRC [45, 46]. The underlying mechanisms include genotoxin‐mediated promotion of mutagenesis, regulation of oncogenic signalling pathways, inflammation induction and immune evasion [45].

**FIGURE 5:**
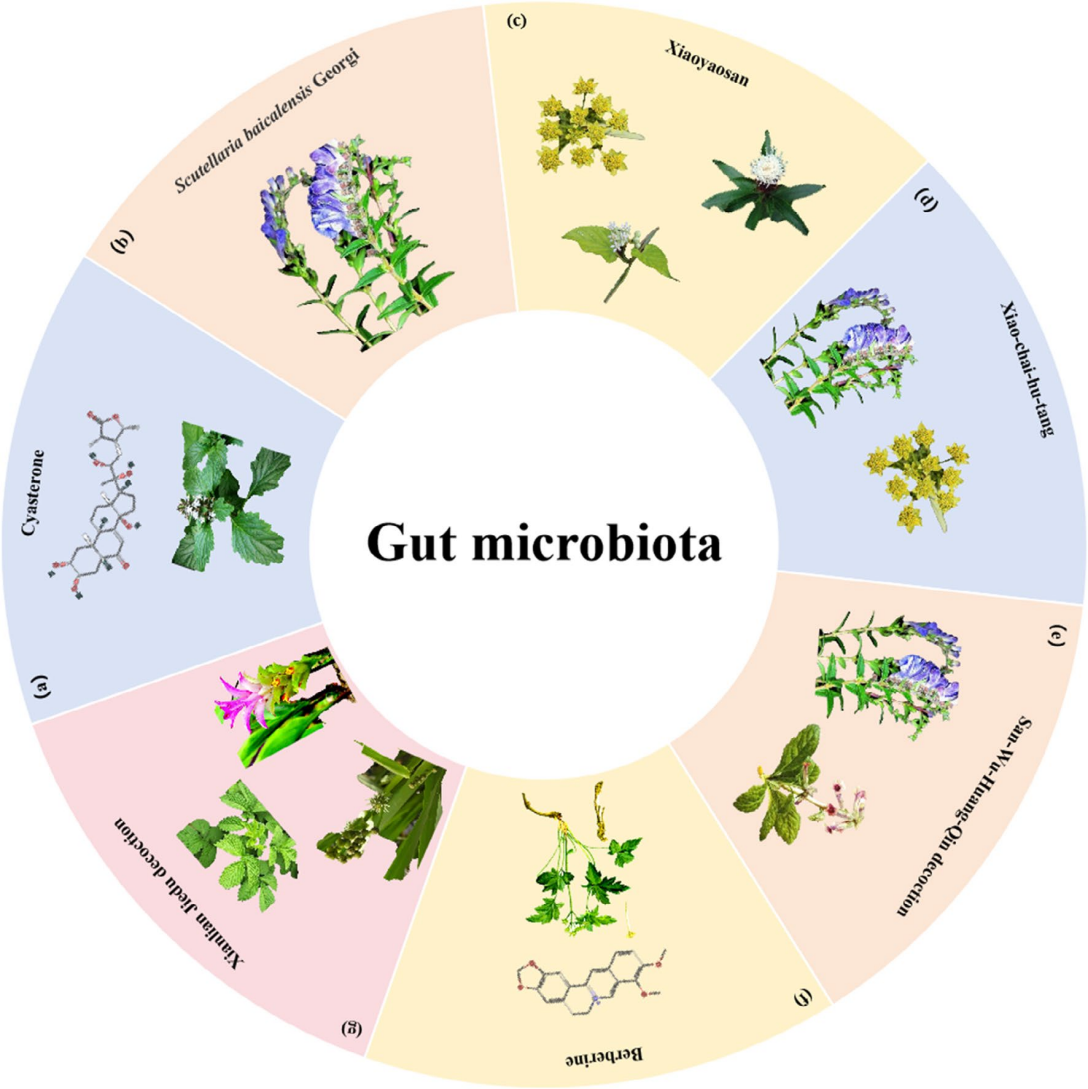
Herbal medicine treats CRC by regulating gut microbiota. (a) *Ajuga decumbens* Thunb. (b) *Scutellaria baicalensi*s Georgi. (c) *Bupleurum chinense* DC., *Atractylodes macrocephala* Koidz. and *Cynanchum otophyllum* Schneid. (d) *Scutellaria baicalensis* Georgi, and *Bupleurum chinense* DC. (e) *Scutellaria baicalensis* Georgi, and *Rehmanniaglutinosa* (Gaertn.) Libosch. ex Fisch. & C. A. Mey. (f) *Coptis chinensis* Franch. (g) *Sparganium stoloniferum* (Buch.‐Ham. ex Graebn.) Buch.‐Ham. ex Juz., *Agrimonia pilosa* Ledeb. and *Curcuma phaeocaulis* Valeton.

Cyasterone is a compound isolated from *Ajuga decumbens* Thunb (Labiatae) or *Cyathula officinalis* Kuan [47]. CRC with mutations in the *BRAF* ^*V600E*^ gene is highly malignant and associated with poor outcomes and prognosis. In a *BRAF* ^*V600E*^‐mutant mouse model of CRC, cyasterone enhanced gut microbiota diversity and beneficial bacteria abundance, including *Prevotellaceae*, *Muribaculaceae* and *Ruminococcaceae*. Although this study demonstrated that cyasterone modulates the abundance of several beneficial bacteria, its efficacy in *BRAF* ^*V600E*^‐mutant CRC was not investigated, and the role of these probiotics remains unclear [47]. Huangqin decoction is often used to treat gastrointestinal symptoms in China for over years. Zhu et al. discovered that Huangqin decoction attenuated colitis and tumour volume, promoted apoptosis and inhibited the PI3K/AKT pathway in AOM/DSS‐induced CRC mice, as well as in SW480 and HT‐29 cells. This effect was associated with an increased abundance of *Clostridium* and elevated levels of faecal butyric acid [48]. Additionally, Ji and colleagues further confirmed that Huangqin decoction enhanced the abundance of *Lachnospiraceae*, *Firmicutes*, *Fusobacteria* and *Clostridium*, while reducing the abundance of *Eggerthellales* in a deoxycholic acid‐induced CRC mouse model [49]. Xiaoyaosan has been used to treat depression in China for hundreds of years. In a CRC xenograft mouse model with depression, Xiaoyaosan effectively suppressed tumour growth and prolonged overall survival by regulating the abundance of *Bacteroides*, *Lactobacillus*, *Desulfovibrio* and *Rikenellaceae* [50]. Shao et al. reported that Xiao‐Chai‐Hu‐Tang, a well‐known TCM prescription, inhibited tumour growth in CRC patients and in a subcutaneous tumour model in C57BL/6J (MC38 cells) under depression by regulating the gut microbiota‐mediated TLR4/MyD88/NF‐κB pathway [51]. This finding may offer promising news for CRC patients suffering from depression. San‐Wu‐Huang‐Qin decoction, a notable TCM prescription, effectively suppressed tumour growth and improved the mucosal barrier in CRC mice, partially by regulating gut microbiota, specifically *Escherichia*‐*Shigella*, a lipopolysaccharide (LPS)‐producing bacterium [52]. Berberine, a compound derived from *Coptis chinensis*, might impede CRC by suppressing the Hedgehog pathway and modulating gut microbiota in CRC mice. Additionally, 5‐fluorouracil has been shown to reduce the α‐diversity of the microbial community in CRC mice [53]. Multiple studies have confirmed that berberine decreases the diversity of intestinal flora in mice with CRC [54, 55, 56, 57]. However, several investigations have suggested that berberine does not have an impact on the diversity of the intestinal microbiota in mice with CRC [58, 59, 60]. Even some studies have found that berberine can significantly increase the diversity of the intestinal microbiota in mice with CRC [61, 62, 63]. The conflicting results may be related to inconsistencies in the preparation protocols for CRC in mice, the sampling sites of intestinal flora, dosing cycles and the presence or absence of environmental contamination during sampling. Cheng et al. found that Xianlian Jiedu decoction, a well‐known TCM prescription, could inhibit tumour growth in CRC mice. Mechanistically, Xianlian Jiedu decoction restored gut microbiota homeostasis and increased levels of short‐chain fatty acids, sphingolipids and glycerophospholipids by inhibiting the abundance of *Turicibacter* and *Clostridium_sensu_stricto_1* [64] (Figure 5).

A comprehensive analysis of the available literature reveals that most research investigating the role of herbal medicine in the treatment of CRC through the modulation of intestinal flora is relatively simplistic. These studies primarily describe changes in the abundance of relevant intestinal flora in CRC models following the application of herbal medicine, often lacking validation experiments and separate investigations into the role of the predominant flora alone. Additionally, it is important to acknowledge that the outcomes of certain animal studies frequently diverge from those observed in clinical trials, particularly with respect to alterations in the composition of intestinal microbiota and the levels of metabolic byproducts. In general, mice exhibit heightened sensitivity to the modulation of intestinal microbiota, likely due to the controlled nature of their living conditions and dietary intake, in contrast to the variable living environments and dietary practices of humans. Consequently, when developing therapeutic strategies for CRC that target gut microbiota, it is essential to meticulously evaluate the influence of diverse dietary habits on the effectiveness of these treatments.

### 2.3 Cancer Stem Cells

Cancer stem cells (CSCs) are unique cells located within tumour tissues with the ability to self‐renew and differentiate into various cell types. They can replicate indefinitely, giving rise to new CSCs. CSCs contribute to cellular heterogeneity, lineage plasticity and drug resistance [65]. Identifying molecular markers and signalling pathways associated with CSCs in CRC can help develop more precisely targeted therapies [66, 67]. Given their significant role in the development of CRC, targeting CSCs has become a vital therapeutic strategy [68].

**FIGURE 6:**
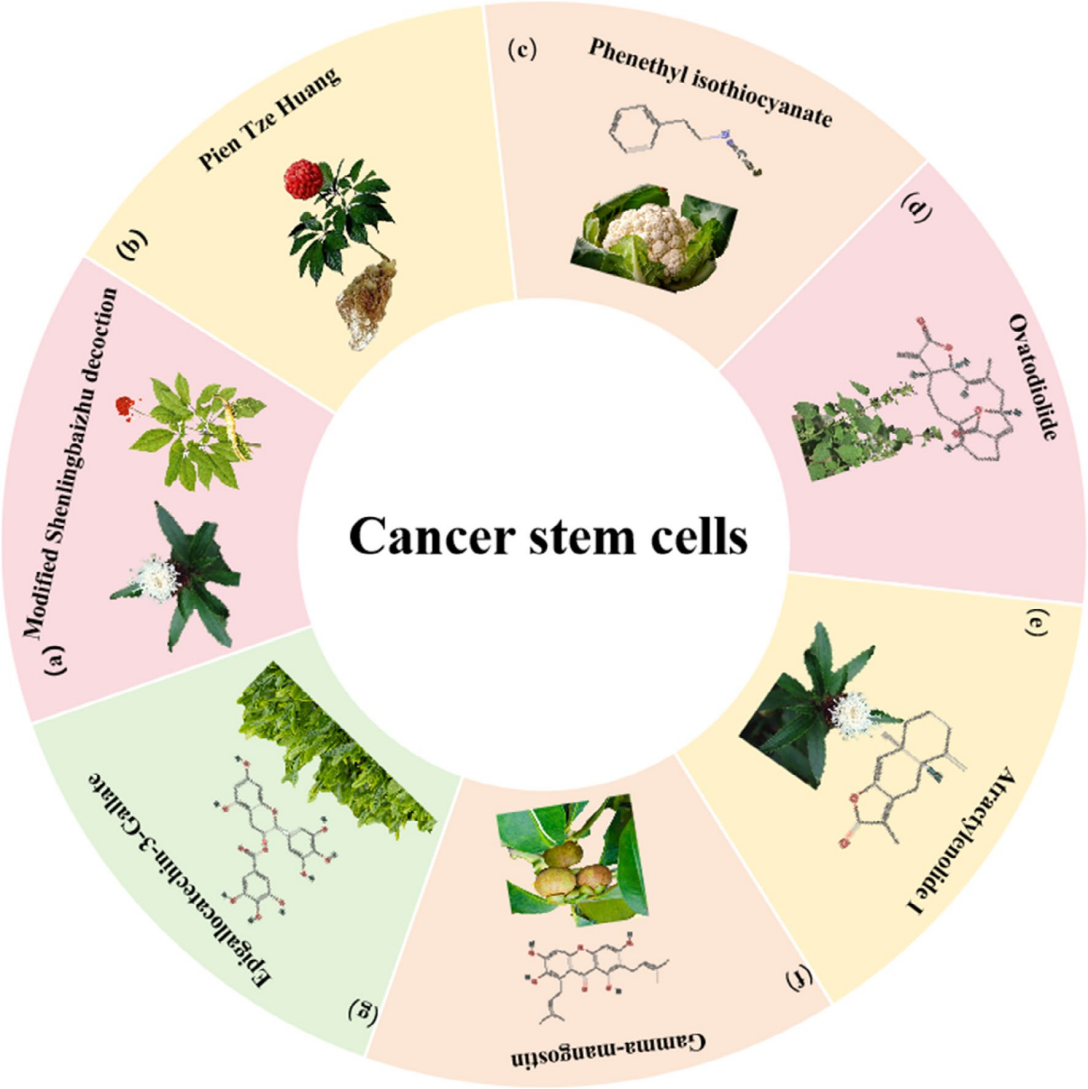
Herbal medicine treats CRC by regulating cancer stem cells. (a) *Panax ginseng* C. A. Mey., *Atractylodes macrocephala* Koidz. (b) *Panax notoginseng* (Burkill) F. H. Chen ex C. H. Chow. (c) *Brassica oleracea var*. *botrytis* Linnaeus. (d) *Anisomeles indica* (L.) O.Ktze. (e) *Atractylodes macrocephala* Koidz. (f) *Garcinia mangostana* L. (g) *Green Tea* polyphenols.

The modified Shenlingbaizhu decoction (MSD), a TCM formula, has been utilised for cancer treatment for several years at Nanfang Hospital in China. Dai proved that MSD significantly inhibited tumours and CD133^+^ CSCs in the tumour tissues of CRC mice. Furthermore, MSD suppressed the pluripotency of CRC cells primarily by inhibiting the epithelial–mesenchymal transition (EMT) induced by TGF‐β/Smad [69]. Pien Tze Huang (PZH), a TCM formula, notably decreased the proportion of CRC stem‐like side population cells and diminished the sphere‐forming capacity of HT‐29 cells, which is beneficial to the treatment of CRC [70]. Peng et al. further demonstrated that PZH exerts a negative regulatory effect on the properties of CSCs in SW480 cells by inhibiting the Notch1 signalling pathway [71]. Additionally, Cao et al. found that PZH augments T‐cell‐mediated cytotoxicity by suppressing the expression of cancer stem cell markers in HCT15, HCT116, as well as in SW480 cells and CRC patient‐derived organoids [72]. These findings underscore the necessity of conducting clinical trials to evaluate the efficacy of PZH in the treatment of CRC. Chu Won Nho et al. found that phenethyl isothiocyanate, a glucosinolate derived from cruciferous vegetables, could prevent CRC growth by inhibiting CSCs in HCT116 cells and a mouse xenograft model by partially regulating inflammation‐mediated tumour microenvironments, although the precise role of inflammation requires further investigation [73]. Han et al. also demonstrated that phenethyl isothiocyanate exerts an inhibitory effect on CSCs in DLD‐1 and SW480 cell lines through the suppression of the Wnt/β‐catenin signalling pathway [74]. Additionally, numerous chemical compounds derived from plants have been shown to inhibit CRC by targeting CSCs, including ovatodiolide [75], atractylenolide I [76], ginsenoside Rg3 [77], epigallocatechin‐3‐gallate [78] and gamma‐mangostin [79] (Figure 6).

### 2.4 Macrophage Polarisation

Macrophages can polarise into M1 and M2 phenotypes within the tumour microenvironment. M1 macrophages exhibit anti‐tumour properties and promote tumour immunotherapy, whereas M2 macrophages facilitate tumorigenesis and metastasis [80]. Furthermore, macrophages are involved not only in tumour immunity but also in the inflammatory environment that precedes tumour formation. This has led to the recognition of macrophages as a significant target for immunotherapy of CRC [81].

**FIGURE 7:**
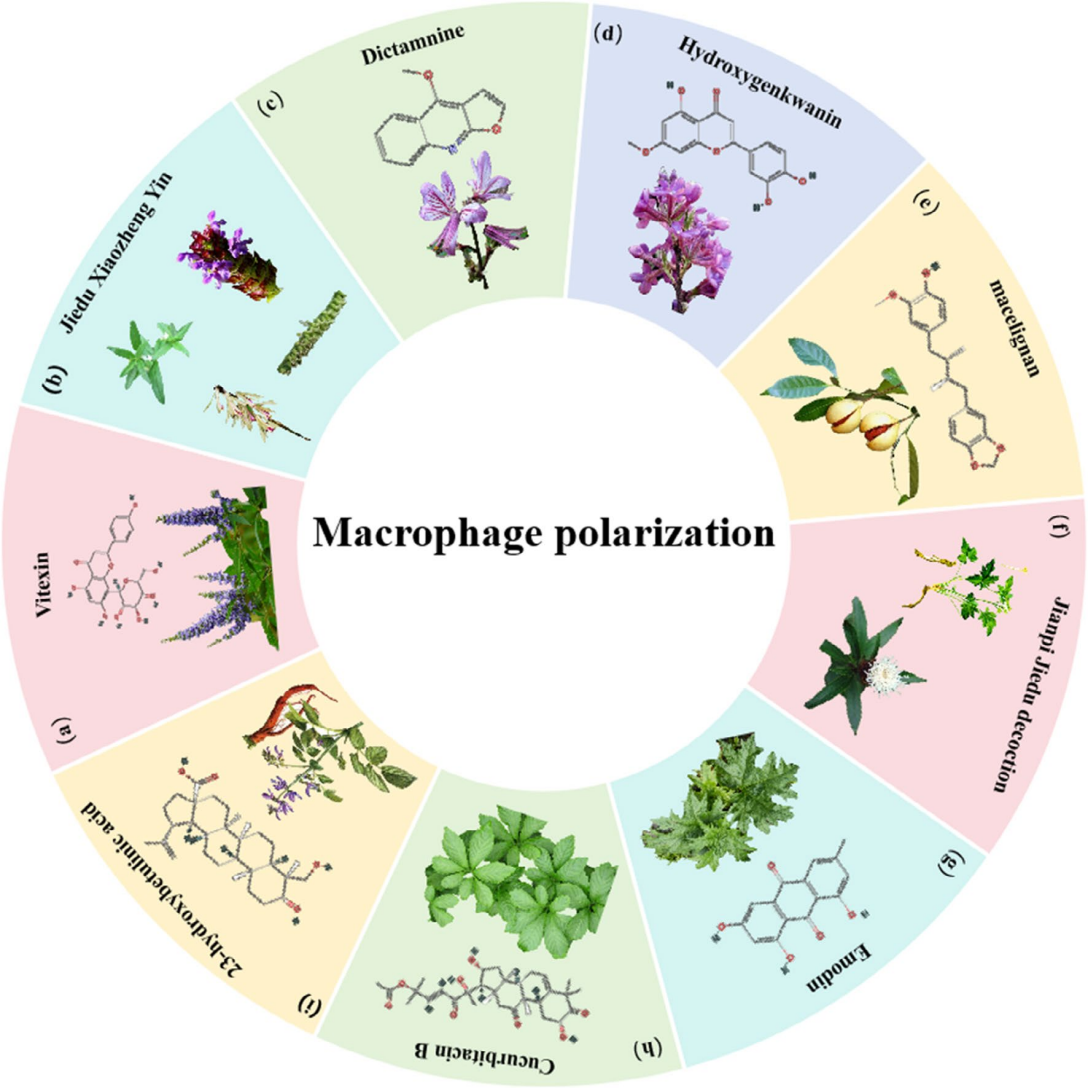
Herbal medicine treats CRC by regulating macrophage polarisation. (a) *Vitex negundo* L. var. cannabifolia (Sieb. et Zucc.) Hand.‐Mazz. (b) *Hedyotis diffusa* Willd, *Spica* prunellae, *Cremastra appendiculata* (D. Don) Makino and *Sophora Flavescens* . (c) *Dictamnus dasycarpus* Turcz. (d) *Daphne genkwa* Siebold & Zucc. (e) *Myristica fragrans* Houtt. (f) *Coptis chinensis* Franch., and *Atractylodes macrocephala* Koidz. (g) *Rheum palmatum* L. (h) *Gynostemma pentaphyllum* (Thunb.) Makino. (i) *Salvia miltiorrhiza* Bunge.

Vitexin (apigenin‐8‐C‐glucoside) is found in various edible and medicinal plants, such as *Crataegus pinnatifida* Bge. and *Vigna radiata* (L.) Wilczek [82]. In AOM/DSS‐induced CRC mice, vitexin has been shown to suppress chronic CAC partly by upregulating M1 macrophage polarisation, which triggers inflammation in colonic tumour tissue. Simultaneously, it significantly inhibits M1 macrophage polarisation in adjacent non‐cancerous tissues, thereby reducing inflammation‐induced carcinogenesis [83]. Tang and colleagues discovered that vitexin targeted the vitamin D receptor (VDR) and influenced macrophage polarisation via the VDR/PBLD (phenazine biosynthesis‐like domain protein) signalling pathway, consequently mitigating the progression from chronic colitis to CRC [84]. This helps us better understand the mechanisms by which vitexin inhibits the development of chronic colitis to CRC. Jiedu Xiaozheng Yin, composed of *Hedyotis diffusa* Willd, *Spica prunellae*, *Pseudobulbus cremastrae* and *Sophora flavescens* , decreases tumour growth by inducing M1 macrophage polarisation through the TLR4 pathway and inhibiting M2 macrophage polarisation in the tumour tissue of CRC mice [85]. Dictamnine, extracted from *Dictamnus dasycarpus* Turcz., inhibits CRC tumour growth by inducing ferroptosis and suppressing M2 macrophage polarisation by regulating the MAPK pathway in DLD‐1 xenograft mice [86]. Additionally, hydroxygenkwanin [87], macelignan [88], Jianpi Jiedu decoction [89], emodin [90], curcumin [91], 23‐hydroxybetulinic acid [92] and cucurbitacin B [93] have also been shown to inhibit M2 macrophage polarisation, thereby contributing to their anti‐metastatic and anti‐proliferative effects against CRC (Figure 7).

### 2.5 Glycolysis

The Warburg effect refers to the phenomenon in which cancer cells primarily obtain energy through glycolysis, a characteristic of tumour metabolism. Furthermore, studies have shown that glycolysis produces various metabolites that favour tumour cell proliferation and metastasis [94]. In recent years, targeting glycolysis has emerged as a significant strategy for exploring treatment options for CRC.

**FIGURE 8:**
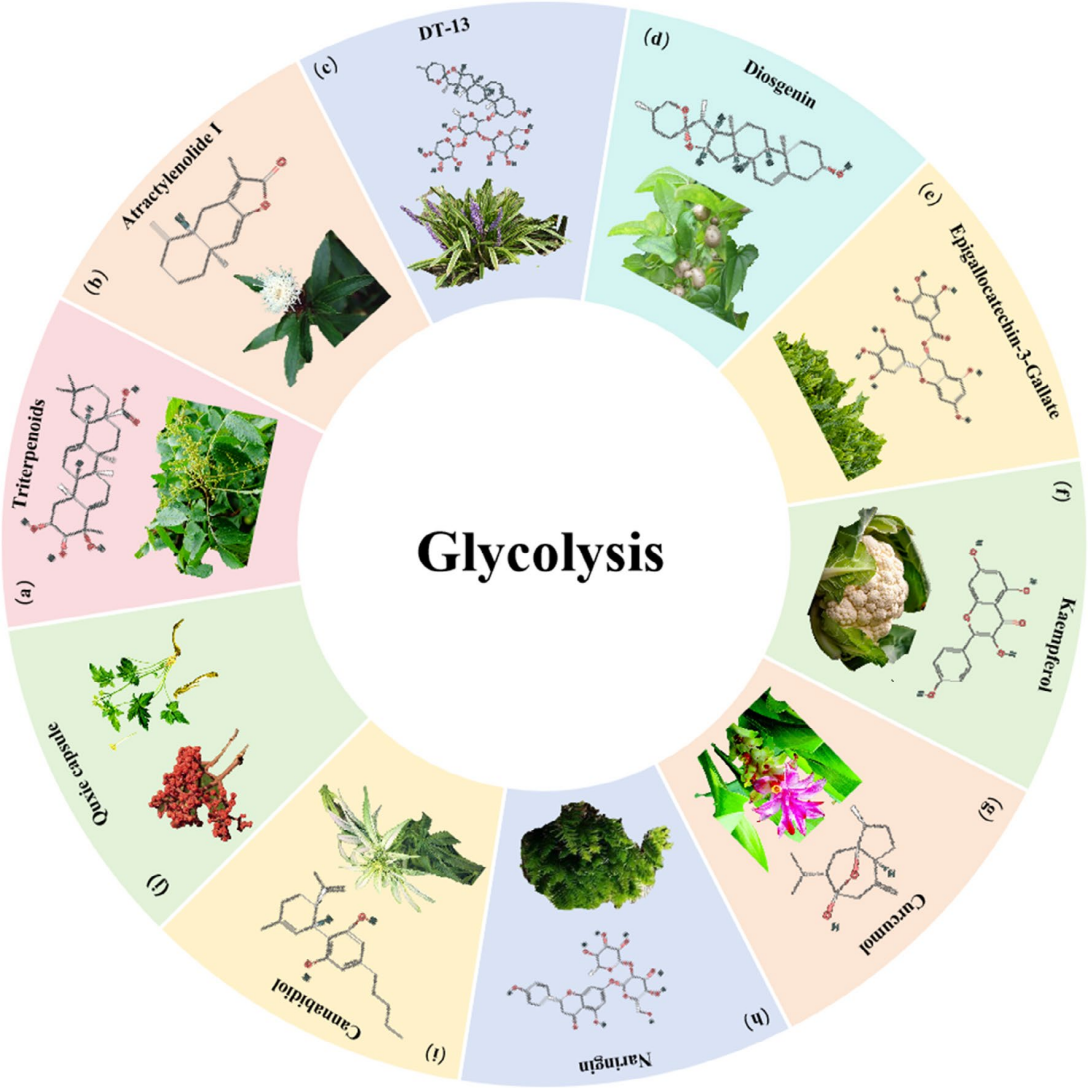
Herbal medicine treats CRC by regulating glycolysis. (a) *Rhus chinensis* Mill. (b) *Atractylodes macrocephala* Koidz. (c) *Liriopes* Radix. (d) *Dioscorea polystachya* Turcz. (e) Green Tea polyphenols. (f) *Brassica oleracea var*. *botrytis* Linnaeus. (g) *Curcuma phaeocaulis* Valeton. (h) *Drynaria roosii* Nakaike. (i) *Cannabis sativa* L. (j) *Evodiae* fructus, and *Coptis chinensis* Franch.

*Rhus chinensis* Mill., a medicinal plant belonging to the *Rhus* genus of the sumac family, is frequently utilised in cancer treatment in China, India and Japan. Wang et al. demonstrated that the triterpenoids (TER) from *Rhus chinensis* Mill. inhibited glycolysis by suppressing glucose uptake and lactate acid production by inhibiting glycolysis gene expressions, including GLUT1, LDHA and PKM2, in SW620 and HCT116 cells [95]. Furthermore, Wang et al. identified that TER not only suppressed glycolysis and glutaminolysis [96] but also promoted glycolytic gene expression in CD8^+^ T cells, thereby enhancing T‐cells recognition and significantly inhibiting CRC tumour growth [16]. Atractylenolide I is a natural compound derived from *Atractylodis Macrocephalae* Rhizoma. Wang et al. found that atractylenolide I significantly affected the Warburg effect and disrupted stemness maintenance by suppressing the AKT/mTOR pathway, which induced cell apoptosis and inhibited invasion in COLO205 and HCT116 cells, as well as in HCT116 xenografted nude mice [76]. Ma et al. provided additional evidence that atractylenolide I induced apoptosis and inhibited glycolysis by obstructing the JAK2/STAT3 signalling pathway in HCT116 and SW480 cells, as well as in HCT116 xenografted nude mouse models [97]. Saponin monomer 13 of the dwarf lilyturf tuber (DT‐13) is a natural product extracted from *Liriopes* Radix, and its anti‐inflammatory and antitumor activities have been reported in several publications [98, 99, 100, 101]. DT‐13 has been shown to block GLUT1‐mediated glycolysis in HCT‐15 and HT‐29 CRC cells, as well as in an HCT‐15 orthotopic nude mouse model of CRC. Additionally, DT‐13 inhibited the number of spontaneous adenomas in the intestines of APC^min^ mice, a model of familial adenomatous polyposis that exhibits a phenotype similar to the human disease, by regulating the AMPK/mTOR pathway [98]. The findings from the orthotopic xenograft implantation mouse model indicated that DT‐13 exhibited a superior antitumor effect at a dosage of 1.25 mg/kg compared to 2.5 mg/kg. This interesting result may be attributed to factors such as the drug's absorption and metabolism, as well as its specific targets and mechanisms of action. Future investigations should focus on elucidating its intrinsic mechanisms by examining its pharmacokinetic and pharmacodynamic properties, conducting molecular target and pathway analyses, validating findings through cell and animal models and assessing metabolism and potential toxicity accumulation. Furthermore, various botanical drugs have been demonstrated to regulate glycolysis to inhibit CRC cell growth, including diosgenin [102], epigallocatechin‐3‐gallate [103], kaempferol [104], curcumol [105], naringin [106], cannabidiol [107] and Quxie capsule [108] (Figure 8).

At present, the detection of tumour glycolysis levels primarily relies on the measurement of glycolytic enzymes and their associated products. However, these methods do not fully capture the true state of glycolysis. As technology continues to advance, it is essential to integrate metabolite quantification, enzyme activity analysis and cutting‐edge techniques in the assessment of tumour glycolysis levels, such as Seahorse XF, fluorescence lifetime imaging microscopy (FLIM) and ^13^C metabolic flux analysis.

### 2.6 Ferroptosis

**FIGURE 9:**
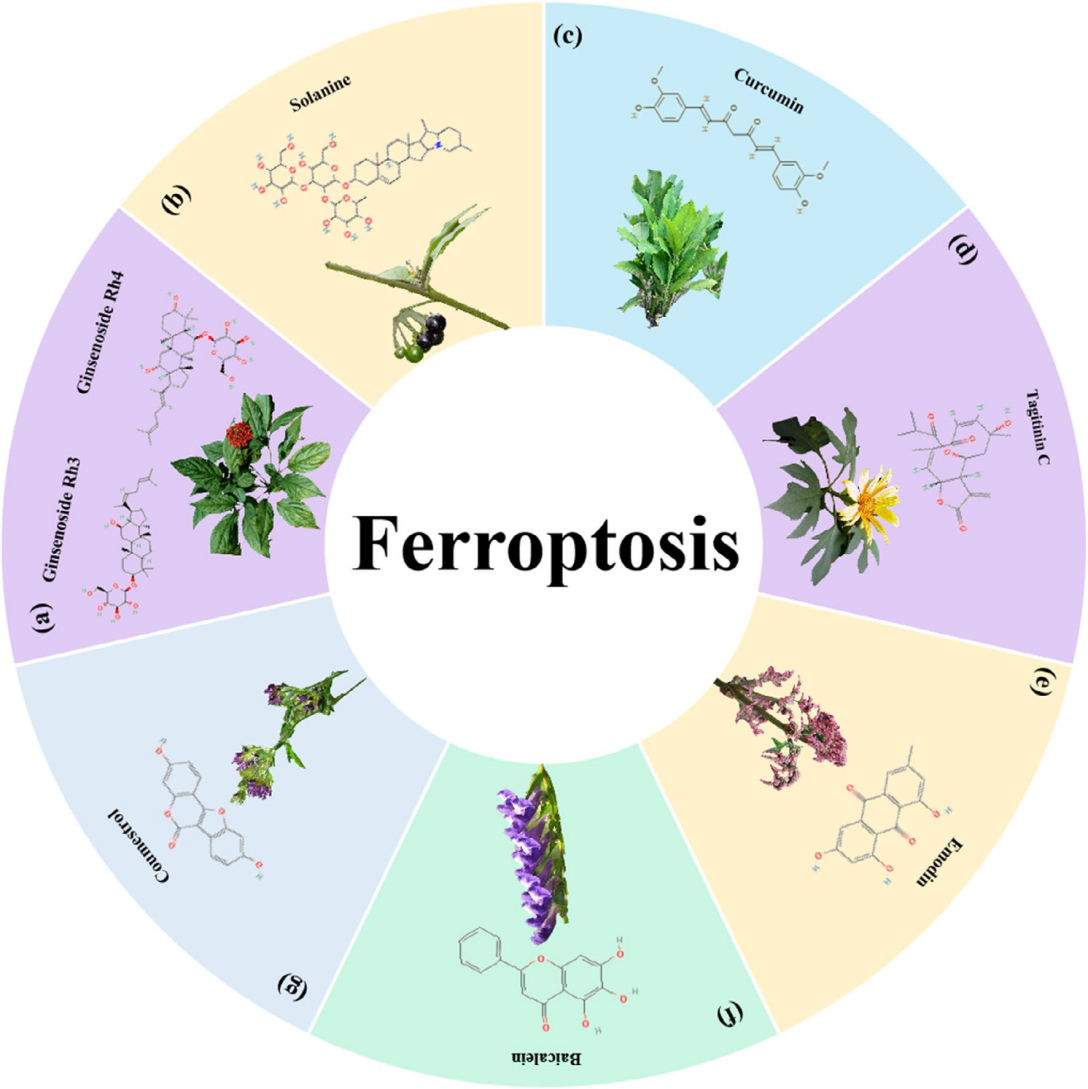
Herbal medicine treats CRC by regulating ferroptosis. (a) *Panax ginseng* C. A. Mey. (b) *Solanum nigrum* L. (c) *Curcuma longa* L. (d) *Tithonia diversifolia* (Hemsl.) A. Grey. (e) *Rheum palmatum* L. (f) *Scutellaria baicalensis* Georgi. (g) *Medicago sativa* L.

**FIGURE 10:**
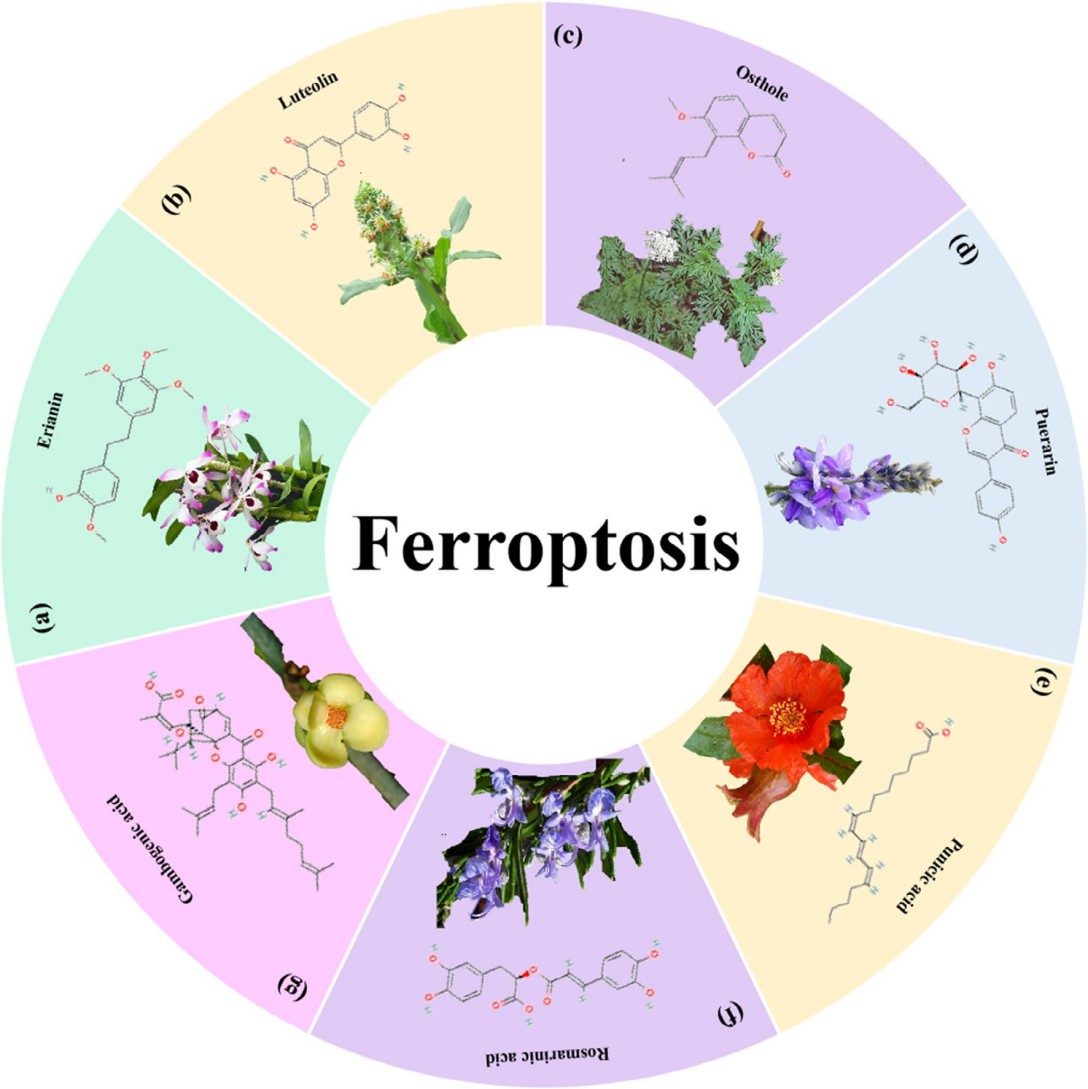
Herbal medicine treats CRC by regulating ferroptosis. (a) *Dendrobium nobile* Lindl. (b) *Reseda odorata* L. (c) *Cnidium monnieri* (L.) Spreng. (d) *Pueraria lobata* (Willd) Ohwi. (e) *Punica granatum* L. (f) *Rosmarinus officinalis* L. (g) *Garcinia hanburyi* Hook.f.

Ferroptosis is a type of cell death that relies on iron and is marked by the accumulation of lipid peroxides, resulting in harm to the membranes of tumour cells. This process leads to several phenomena, including increased lipid peroxidation, failure of antioxidant defences, elevated ROS, mitochondrial atrophy and cell membrane rupture [109, 110, 111]. As the investigation of ferroptosis as a potential treatment for CRC is still in its early stages, further investigation is needed to determine how to effectively trigger ferroptosis in CRC cells while minimising disruption to normal cells [112]. Ginsenoside Rh3 (GRh3), a bacterial byproduct of ginsenoside Rg5, induces ferroptosis via triggering gasdermin D‐dependent pyroptosis and suppressing solute carrier family 7 member 11. This inhibition activates ferroptosis through the Stat3/p53/NRF2 pathway in HT29 and HCT116 cells, as well as in HT29 and HCT116 xenograft models in nude mice [113]. In vivo studies have indicated that GRh3 does not cause substantial harm to hepatic and renal tissues. Furthermore, in vitro investigations have revealed that GRh3 does not exert a significant inhibitory effect on normal colorectal cells [113]. Therefore, the current research data indicate that GRh3 can significantly inhibit CRC and has no toxic or side effects on normal tissues. Solanine, a steroidal alkaloid, is the main chemical component of *Solanum nigrum* L [114]. Lu et al. found that solanine could induce ferroptotic cell death through the ALOX12B/ADCY4 molecular axis in HCT116 and SW480 cells [115]. Curcumin, the main component of *Curcumae Longae* Rhizoma, is proven to trigger ferroptosis by regulating the JNK signalling pathway [116], suppressing glutathione peroxidase‐4 and ferroptosis suppressor protein‐1 (in combination with andrographis) [117], inhibiting the PI3K/AKT/mTOR signalling pathway [118] and regulating the p53 and solute carrier family 7 member 11/glutathione/glutathione peroxidase 4 signalling axis [119]. Studies have demonstrated that curcumin can synergistically enhance the anti‐tumour efficacy of chemotherapeutic agents (e.g., 5‐fluorouracil) and natural compounds (e.g., andrographolide) while exhibiting low toxicity toward normal tissues. However, its clinical application is hindered by poor bioavailability, primarily attributed to low water solubility, which limits drug absorption and accelerates metabolic clearance. To address this challenge, research efforts are focused on developing advanced delivery systems, such as nanomaterial‐based carriers or plant extracellular vesicles. These technologies aim to improve drug solubility, enable targeted delivery and enhance in vivo stability, thereby unlocking the full therapeutic potential of curcumin. The role of ferroptosis in CRC has garnered increasing attention and has been demonstrated in the mechanisms of action of various botanicals, including tagitinin C [120], emodin [121], baicalein [122], coumestrol [123], erianin [124], luteolin [125], osthole [126], puerarin [127], punicic acid [128], rosmarinic acid [129], ginsenoside Rh4 [130] and gambogenic acid [131] (Figures 9 and 10).

Currently, investigations into ferroptosis predominantly rely on in vitro experiments, with a limited number of associated animal models. This disparity arises from the significantly more intricate metabolic processes that occur in vivo compared to those observed in cellular studies. Furthermore, existing research utilising animal models primarily distinguishes between control and drug treatment groups, with a notable absence of positive control groups. Future studies on the mechanisms underlying ferroptosis should aim to enhance the validation of findings through in vivo experimentation.

### 2.7 Extracellular Vesicles

**FIGURE 11:**
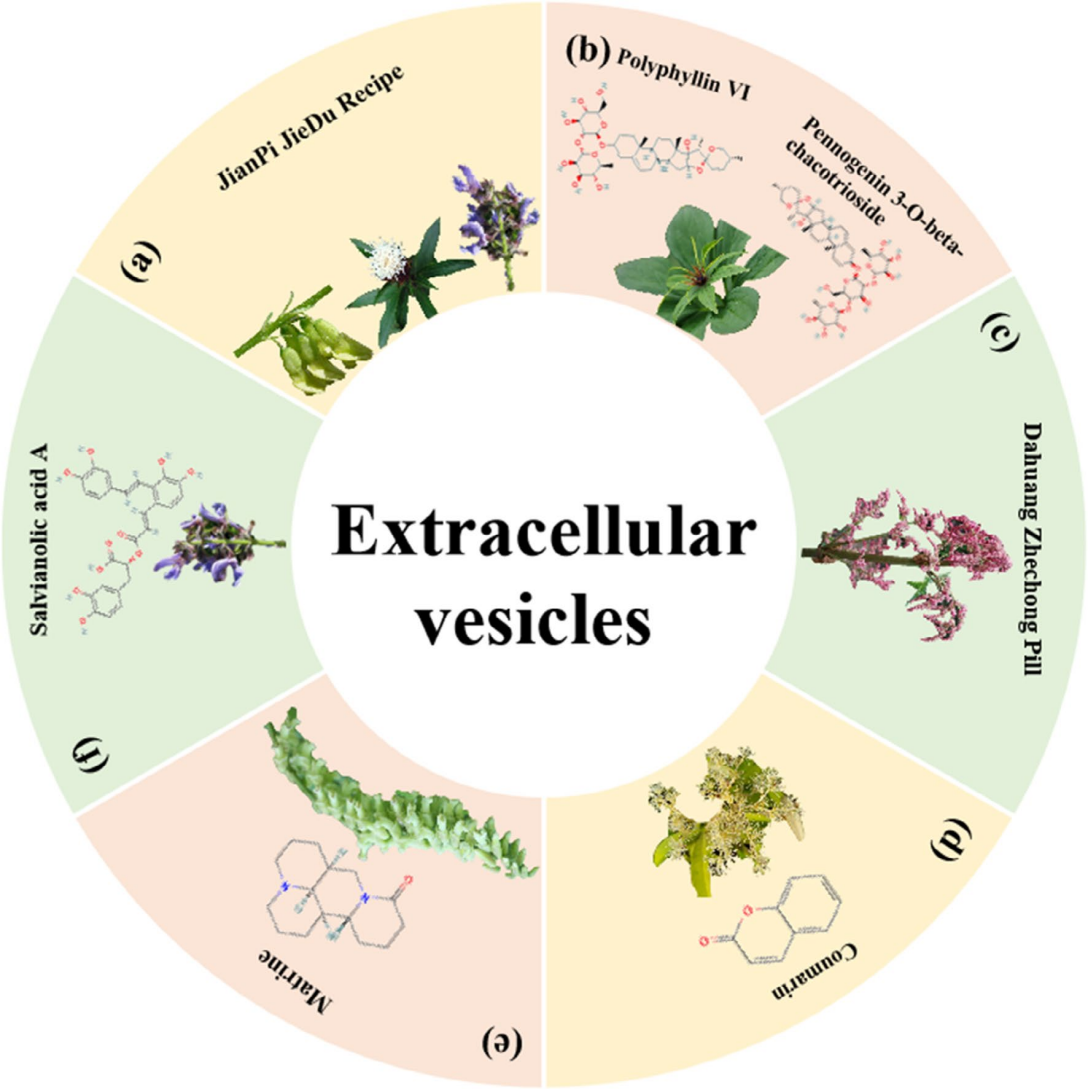
Herbal medicine treats CRC by regulating extracellular vesicles. (a) *Astragalus membranaceus* (Fisch.) Bunge, *Atractylodes macrocephala* Koidz. and *Salvia miltiorrhiza* Bunge. (b) *Paris polyphylla* Sm. (c) *Rheum palmatum* L. (d) *Pileostegia tomentella* Hand. Mazz (Saxifragaceae). (e) *Sophora flavescens* Ait. (f) *Salvia miltiorrhiza* Bunge.

An exosome is a membrane‐bound vesicle released from a cell through the fusion of an intracellular multivesicular body with the cell membrane. Exosomes transport nucleic acids, proteins, lipids and other biological macromolecules. They bind to surface receptors on recipient cells via specific surface molecules, facilitating the delivery of their contents for intercellular communication. Increasing evidence indicates that exosomal miRNAs serve as crucial mediators of communication between tumour cells and the tumour microenvironment [132, 133]. The modulation of exosome levels containing specific contents represents a promising approach for CRC treatment [134, 135]. JianPi JieDu Recipe (JPJDR), a TCM prescription, has been frequently utilised to treat gastrointestinal tumours. Ji and colleagues discovered that JPJDR suppressed the growth and migration of CRC cells by modulating the ITGBL1‐rich extracellular vesicle‐mediated TNFAIP3‐NF‐κB pathway, which subsequently inhibited fibroblast activation [136]. *Paris polyphylla*, an herbal medicine, has demonstrated various physiological activities, including antifungal, antidiabetic, anti‐inflammatory, antioxidant and immunomodulatory effects and potential benefits against digestive tract cancers [137, 138, 139, 140, 141]. Tai and colleagues found that pennogenin 3‐O‐beta‐chacotrioside and polyphyllin VI, both extracted from *Paris polyphylla*, could inhibit the release of extracellular vesicles from *Fusobacterium nucleatum* , an intestinal bacterium enriched in CRC lesions and associated with CRC proliferation and metastasis, thereby interfering with the invasion of Caco‐2 and HT‐29 cells [142]. Furthermore, various chemical compounds derived from plants have been found to regulate extracellular vesicles to inhibit CRC tumour growth or metastasis. For example, matrine inhibits the secretion of exosomal circSLC7A6 by regulating CXCR5 [143]; salvianolic acid A inhibits the amount of GRP78 in exosomes [144]; Dahuang Zhechong Pill ameliorates exosomal CCL2 primed pre‐metastatic niche [145]; and total coumarins extracted from *Pileostegia tomentella* Hand. Mazz (Saxifragaceae) upregulate exosomal miR‐375‐3p [146] (Figure 11).

Current research on the molecular mechanisms by which herbal medicines exert therapeutic effects against CRC through extracellular vesicle modulation remains in its early stages. Specifically, the upstream and downstream regulatory mechanisms by which certain drugs inhibit CRC are not yet fully elucidated. Furthermore, many studies lack validation in animal models and subsequent verification of clinical efficacy. Additionally, the active components of herbal formulations and the synergistic effects of component combinations remain unclear. Further investigation is also required to evaluate drug bioavailability, in vivo metabolic stability and long‐term toxicity.

### 2.8 Mitochondrial Function

**FIGURE 12:**
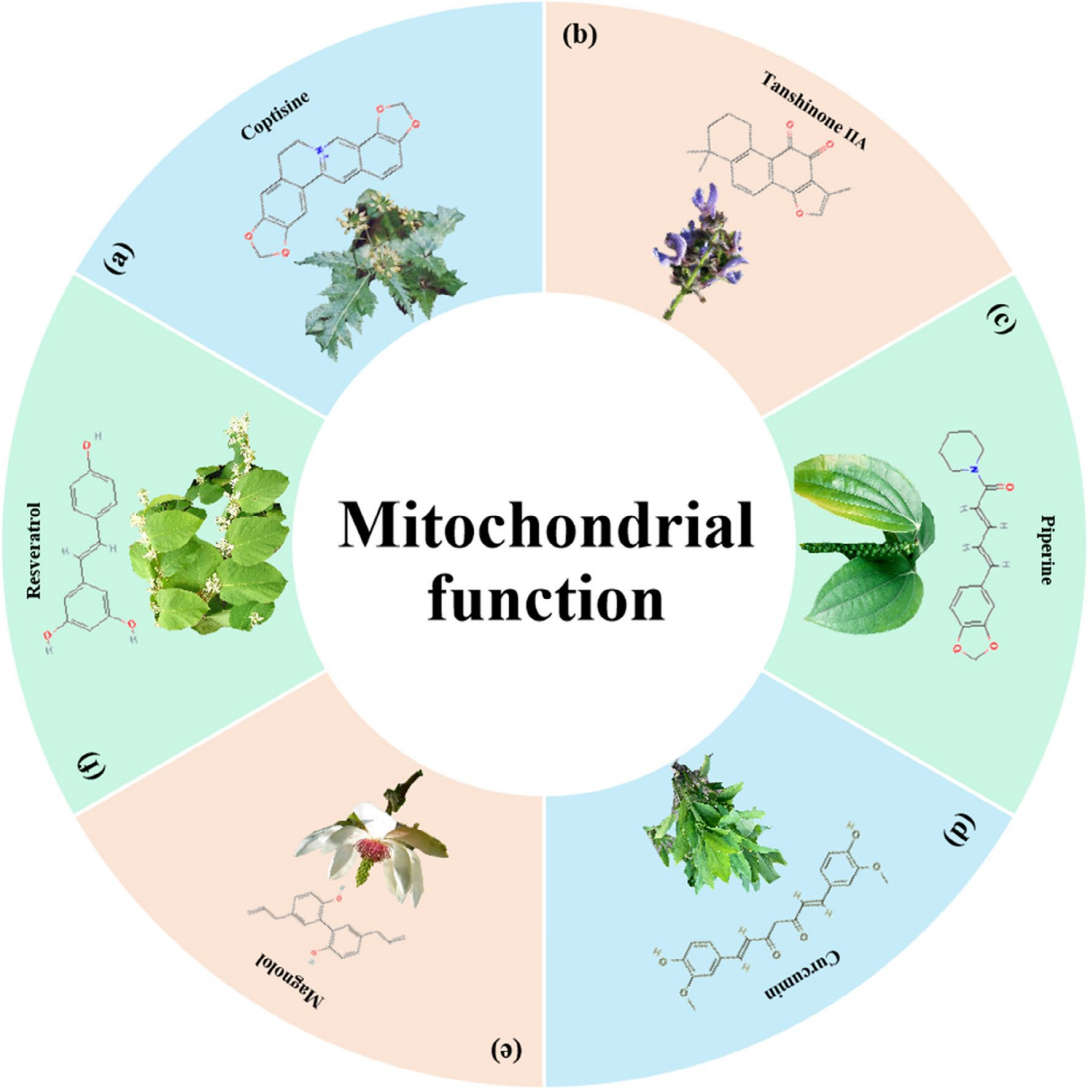
Herbal medicine treats CRC by regulating mitochondrial function. (a) *Coptis chinensis* Franch. (b) *Salvia miltiorrhiza* Bunge. (c) *Piper nigrum* L. (d) *Curcuma longa* L. (e) *Magnolia officinalis* Rehd. et Wils. (f) *Polygonum cuspidatum* Sieb. et Zucc.

**FIGURE 13:**
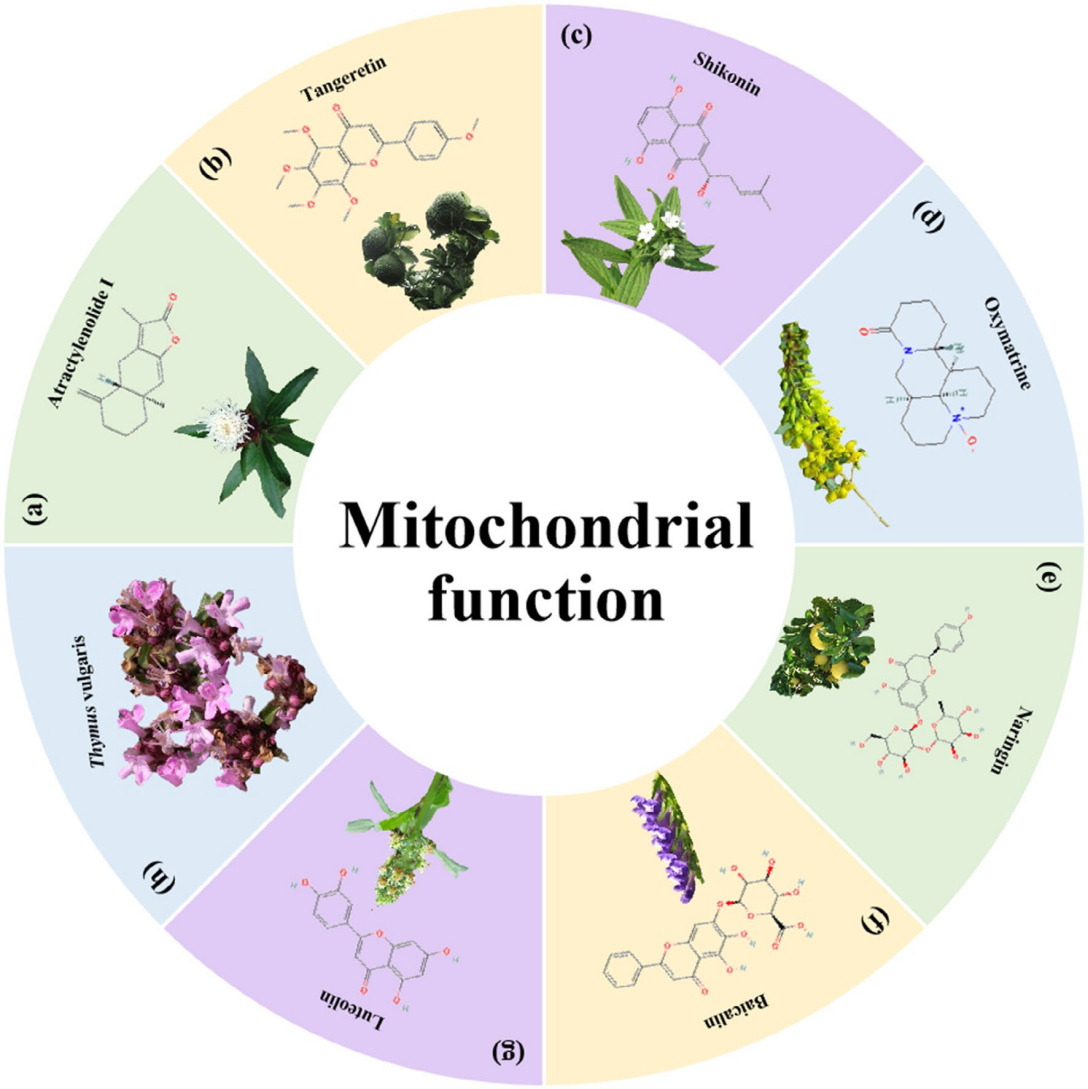
Herbal medicine treats CRC by regulating mitochondrial function. (a) *Atractylodes macrocephala* Koidz. (b) *Citrus aurantium* L. (c) *Lithospermum erythrorhizon* Siebold & Zucc. (d) *Sophora tonkinensis* Gagnep. (e) *Citrus paradisi* Macf. (f) *Scutellaria baicalensis* Georgi. (g) *Reseda odorata* L. (h) *Thymus mongolicus* (Ronniger) Ronniger.

Abnormal mitochondrial function can lead to metabolic imbalances and altered redox status, which may promote the development of cancer. Conversely, mitochondria can also influence cancer progression by participating in processes such as apoptosis. Given the important role of mitochondria in CRC, treatment strategies targeting mitochondrial function are gaining increasing attention [147, 148]. Coptisine is an isoquinoline alkaloid extracted from *Coptis chinensis* Franch. Han et al. proved that coptisine significantly suppressed cytoactive and migration in HCT‐116 cells by disrupting mitochondrial function, which was indicated by a lower mitochondrial membrane potential (MMP) and increased levels of ROS. Consequently, Han et al. speculated that coptisine promoted HCT‐116 cell apoptosis via activating mitochondrial‐related apoptotic pathways [149]. Furthermore, an analysis of the visceral index and mortality rates in the coptisine‐treated animals revealed no indications of drug‐related toxicity. These results collectively support the conclusion that coptisine is a compound characterised by low toxicity and possesses anti‐tumour properties [149]. Tanshinone IIA, a main natural product derived from *Salvia miltiorrhiza* Bunge, has demonstrated a range of physiological activities, including effects on hepatic steatosis, insulin resistance, cardiovascular disorders and cancers [150, 151, 152]. Recent evidence suggests that excessive mitochondrial fission disrupts mitochondrial physiological function, leading to a decrease in MMP, an increase in mitochondrial ROS level and ultimately triggering mitochondria‐mediated apoptosis [153]. Li et al. found that tanshinone IIA inhibited the proliferation and migration of SW837 cells by triggering the JNK‐Mff pathway, which mediates mitochondrial fission [153]. Huo et al. demonstrated that tanshinone IIA promotes IL2‐mediated SW480 cell apoptosis through the activation of INF2‐mediated mitochondrial fission and the stimulation of the Mst1‐Hippo signalling pathway [154]. However, all of the aforementioned research results are based on cell experiments, and further animal experiments and clinical trials are necessary to support these findings. Piperine is the major bioactive compound found in black pepper ( *Piper nigrum* ). Srikanta Kumar Rath et al. found that piperine enhanced the oral bioavailability of CXB (a selective COX‐2 inhibitor) by 129% in adult female BALB/c mice. Additionally, piperine increased the toxicity of CXB to HT‐29 cells, but not to IEC‐6 cells, by inducing mitochondria‐mediated apoptosis. This was evidenced by higher ROS levels, caspase activation and increased apoptotic protein expressions. Furthermore, the combination of piperine and CXB reduced the population of CD133^+^/CD44^+^ CSCs and stemness‐related protein expressions. In the CT26 xenograft BALB/c mouse model, piperine and CXB synergistically suppressed tumour growth, as indicated by the tumour volume in the piperine and CXB group, which increased by 20.9% compared to the vehicle group (77.9%), the CXB group (66.9%) and the piperine group (75.4%). Mechanistically, piperine synergizes with CXB to suppress CRC cell proliferation by regulating the Wnt/β‐catenin pathway and inducing mitochondria‐mediated apoptosis in HT‐29 cells [155]. The role of piperine in inhibiting the proliferation of CRC by regulating mitochondrial‐mediated apoptosis has also been confirmed by multiple studies [156, 157, 158, 159]. However, all the above research results are based on in vitro cell experiments, and further animal experiments are necessary to validate these findings. What is more, considering that pepper is commonly used as a flavouring agent, its consumption may influence the bioavailability, efficacy and even toxicity of certain drugs. Therefore, more comprehensive studies are necessary. Mitochondrial‐associated apoptosis is a crucial mechanism involved in the action of various compounds against CRC, including curcumin [160], magnolol [161], resveratrol [162], atractylenolide I [163], tangeretin [164], shikonin [165], oxymatrine [166], naringin [167], baicalin [168], luteolin [169] and *Thymus vulgaris* [170] (Figures 12 and 13).

### 2.9 Inflammation

**FIGURE 14:**
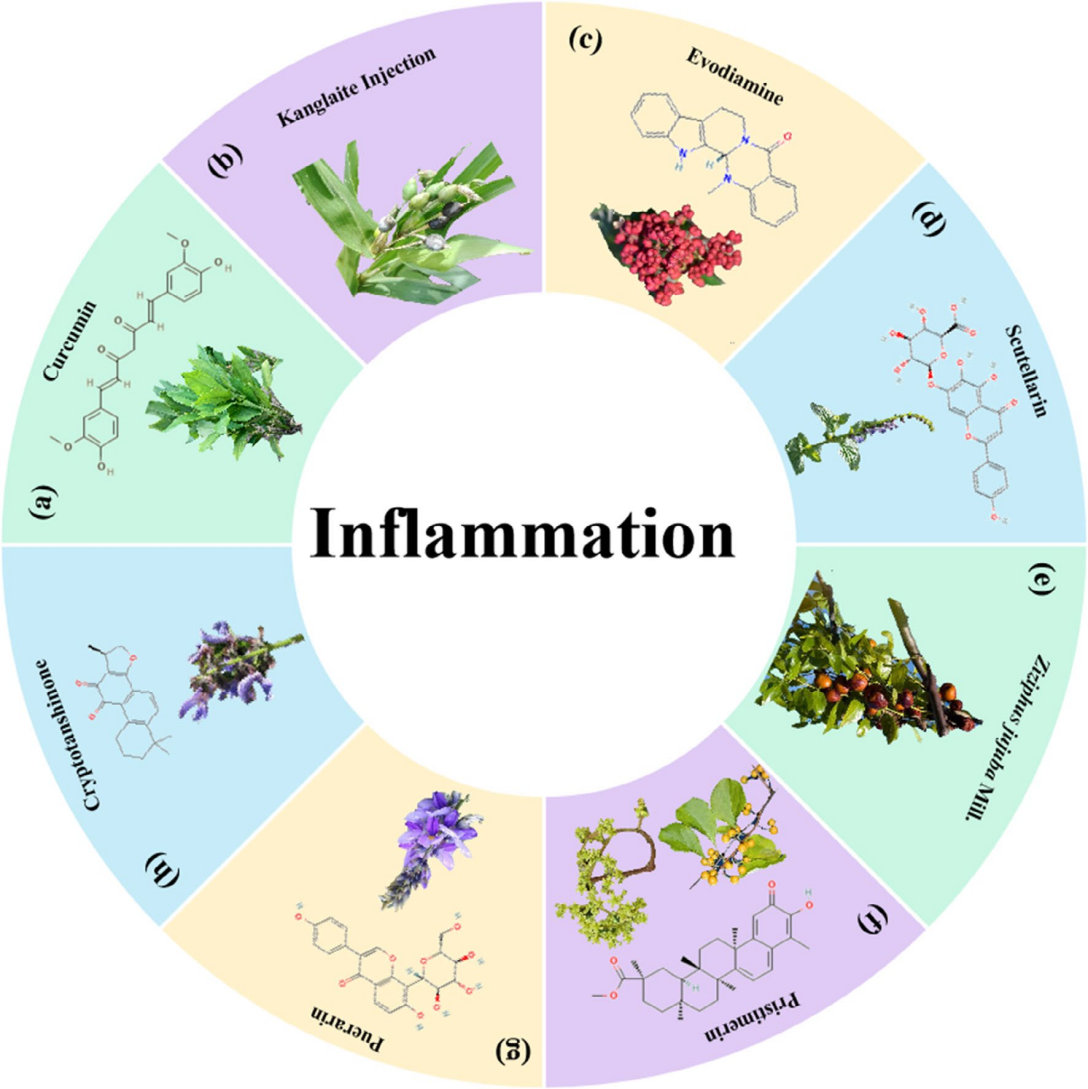
Herbal medicine treats CRC by regulating inflammation. (a) *Curcuma longa* L. (b) *Coix lacryma*‐*jobi* (adlay) seed. (c) *Tetradium ruticarpum* (A. Juss.) T. G. Hartley. (d) *Scutellaria altissima* L. (e) *Ziziphus jujuba* Mill. (f) *Celastraceae and Hippocrateaceae* families. (g) *Pueraria lobata* (Willd.) Ohwi. (h) *Salvia miltiorrhiza* Bunge.

Chronic inflammatory stimulation is a significant contributor to CRC, which accounts for the heightened risk of CRC in individuals with ulcerative colitis (UC) [171]. The secretion of pro‐inflammatory cytokines is a hallmark of the chronic inflammatory response and accelerates tumour growth and metastasis [172]. Exploring botanical treatments for CRC may be beneficial, and increasing the intake of an anti‐inflammatory diet is also an effective strategy for preventing CRC in patients with UC [173, 174]. Curcumin, a flavonoid derived from *Curcuma longa* L., is proven to promote SW480 and HCT116 cell apoptosis by activating the NLRP3 inflammasome. However, the mechanisms underlying the promotion of apoptosis in LoVo and HT‐29 cells remain unclear, suggesting that NLRP3 inflammasome activation is only one of the pathways by which curcumin causes CRC cell apoptosis [175]. The role of curcumin in inducing apoptosis in CRC cells by regulating inflammation has been confirmed by multiple studies [176, 177]. Although several Phase I clinical studies have clearly demonstrated the safety of oral curcumin, high concentrations of curcumin have been found to potentially provoke glutathione depletion and caspase‐3 activation, which may cause hepatocytotoxicity in rat hepatocytes [178]. Furthermore, Torti and colleagues discovered that curcumin could induce iron depletion in mice with low iron content in the body by inhibiting hepcidin. This might be an important consideration when curcumin is used to treat patients with borderline iron deficiency or those exhibiting anaemia due to diseases [179]. Kanglaite injection is an extract derived from *Coix lacryma*‐*jobi* (adlay) seed. Wang et al. demonstrated that Kanglaite enhanced the sensitivity of CRC cells to Taxol by suppressing NF‐κΒ and promoting connexin 43 in a mouse model with subcutaneous tumours [180]. Shi et al. also proposed that Kanglaite inhibits EMT by inhibiting NF‐κΒ in CRC cells as well as in a mouse model with subcutaneous tumours using CT26 cells [181]. Evodiamine is an anti‐tumour active ingredient extracted from *Tetradium ruticarpum* (A. Juss.) T. G. Hartley [182]. Sui et al. found that evodiamine could inhibit ABCG2‐mediated drug resistance by suppressing NF‐κB in HCT‐116/L‐OHP cells and in a colorectal multidrug‐resistant cancer xenograft model in nude mice [182]. Zhao et al. also found that evodiamine promoted apoptosis and suppressed migration of HCT‐116 cells by suppressing the JAK2/STAT3 pathway, which activates NF‐κB [183]. The effect of evodiamine in regulating inflammation against CRC has also been supported by numerous studies [184, 185, 186, 187]. Scutellarin, a phytochemical flavonoid derived from *Scutellaria altissima* L., inhibits CAC by suppressing the Hedgehog pathway and the NF‐κB‐mediated inflammatory response in mice with AOM/DSS‐induced CRC [188]. Xu and colleagues further discovered that scutellarin suppressed serum TNF‐α and IL‐6 levels in mice, increased the expression of Bax and decreased the levels of Bcl‐2 in the CAC tissues by inhibiting the Wnt/β‐catenin signalling pathway. These findings suggest that scutellarin may mitigate inflammation through multiple mechanisms, thereby exerting a therapeutic effect on CAC [189]. Jujube ( *Ziziphus jujuba* Mill.), a common fruit in the Rhamnaceae family, has been used for decades to treat CRC. Ruan et al. found that the bioactive triterpenes in jujube prevented CRC by inhibiting the PI3K/AKT/NF‐κB pathway. Various triterpenes were identified in this study, which significantly contributed to subsequent development efforts [190]. Pristimerin is a triterpenoid derived from the *Celastraceae* and *Hippocrateaceae* families. Yousef et al. found that pristimerin prevented CRC growth by inhibiting the NF‐κB pathway in HCT‐116 cells and in a BALB/c nude mouse model with HCT‐116 xenografts [191, 192]. Kim and colleagues further discovered that the administration of pristimerin resulted in a reduction in inflammation and cellular proliferation triggered by AOM/DSS in colonic tissue. Additionally, pristimerin promoted apoptosis and modulated the AKT/FOXO3a signalling pathway [193]. Puerarin is an isoflavone isolated from *Pueraria lobata* (Willd.) Ohwi, with significant anti‐CRC activity [127, 194, 195, 196, 197]. Nonetheless, the clinical efficacy of puerarin is limited due to its poor solubility and low bioavailability. Deng et al. prepared colon‐specific microspheres loaded with puerarin by emulsification/internal gelation, which inhibited tumorigenesis and metastasis in CRC, partly by suppressing inflammation [198]. However, the mechanism by which puerarin exerts its therapeutic effects on CRC by regulating inflammation still requires further research for confirmation. Cryptotanshinone, a natural ingredient derived from *Salvia miltiorrhiza* Bunge, has been shown to significantly suppress the proliferation and growth of CRC [199, 200, 201]. Li et al. confirmed that cryptotanshinone induced CRC cell apoptosis by inhibiting Stat3, a known NF‐κB activator [199]. Zhang et al. found that cryptotanshinone prevented the proliferation and metastasis of CRC by inhibiting inflammation and angiogenesis by the regulation of the MMP/TIMP system, the PI3K/AKT/mTOR pathway and the HIF‐1α pathway in CT26 cells and a CT26 xenograft model using BALB/c mice [202]. These findings further suggest that the inflammatory response plays a crucial role in CRC treatment through the action of cryptotanshinone (Figure 14).

### 2.10 Oxidative Stress

Growing evidence indicates that chronic oxidative stress can activate inflammatory signalling pathways, which may subsequently trigger various transcription factors or lead to the deregulation of gene expression, thereby influencing tumour development and the survival of cancer cells. Additionally, oxidative stress causes excessive lipid peroxidation, and several lipid peroxidation products, including 4‐hydroxynonenal and acrolein, have been implicated in the development of CRC [203, 204]. Zerumbone, a sesquiterpene derived from the edible ginger ( *Zingiber zerumbet* Smith), has been shown to suppress CRC by various mechanisms, including the modulation of oxidative stress, gut microbiota and inflammation [205, 206, 207, 208, 209, 210, 211, 212]. Memari et al. also found that zerumbone promoted apoptosis and suppressed metastasis in HT‐29 cells by elevating ROS levels [210]. Sithara et al. demonstrated that zerumbone induces apoptosis in SW480 cells, partly by increasing cellular ROS levels and decreasing antioxidant levels [213]. Dihydroartemisinin is a well‐known antimalarial drug derived from *Artemisia caruifolia* Buch.‐Ham. ex Roxb. Yu et al. found that dihydroartemisinin promoted the anti‐tumour activity of oxaliplatin by altering the PRDX2‐ROS pathways in HCT116 and RKO cells, as well as in an HCT116 xenograft model using nude mice [214]. Additionally, Yao et al. found that dihydroartemisinin promoted the antitumor capacity of 5‐fluorouracil against the resistant HCT116 TP53^−/−^ cells through ROS‐mediated apoptosis [215]. However, the mechanisms by which dihydroartemisinin exerts its therapeutic effects on CRC by regulating oxidative stress still require more research data from various cell lines and animal models for confirmation. Dihydromyricetin is a flavonoid compound extracted from the leaves of *Vitis heyneana* Roem. et Schult. Wang et al. found that dihydromyricetin counteracted multidrug resistance caused by MRP2 by inhibiting the NF‐κB‐Nrf2 pathway in HCT116 and HCT8 cells, as well as in a xenograft model using BALB/c mice with NF‐κB/p65‐overexpressing HCT116/OXA cells [216]. Zhang et al. believed that the involvement of semaphoring 4D is essential for the initiation of the anti‐oxidant and anti‐inflammatory properties of dihydromyricetin in colon cancer [217]. Andrographolide is a bicyclic diterpenoid lactone extracted from *Andrographis paniculata* (Burm.f.) Nees. Khan and colleagues discovered that andrographolide suppressed the proliferation of SW‐480, DLD‐1, HT‐29 and HCT‐116 cells via augmenting intracellular ROS generation [218, 219, 220, 221]. Additionally, Banerjee et al. reported that andrographolide induced apoptosis in T84 and COLO205 cells by triggering ROS [222]. Andrographolide could also enhance the anti‐tumour capacity of melatonin and cisplatin through ROS‐mediated ER stress [223, 224].

**FIGURE 15:**
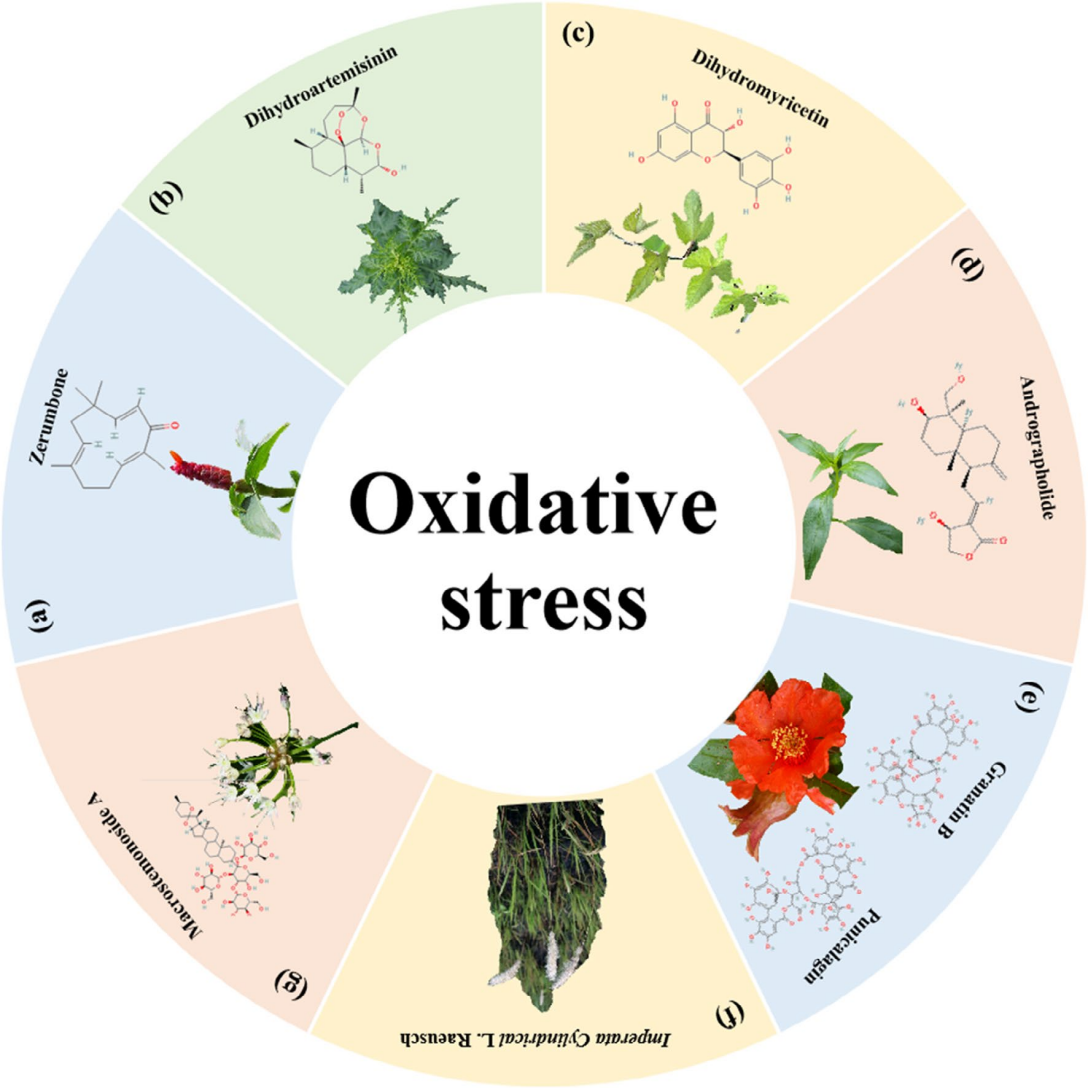
Herbal medicine treats CRC by regulating oxidative stress. (a) *Zingiber zerumbet* Smith. (b) *Artemisia annua* L. (c) *Vitis heyneana Roem*. et Schult. (d) *Andrographis paniculata* (Burm. f.) Nees. (e) *Punica granatum* L. (f) *Imperata Cylindrical* L. Raeusch. (g) *Allium macrostemon* Bunge.

**FIGURE 16:**
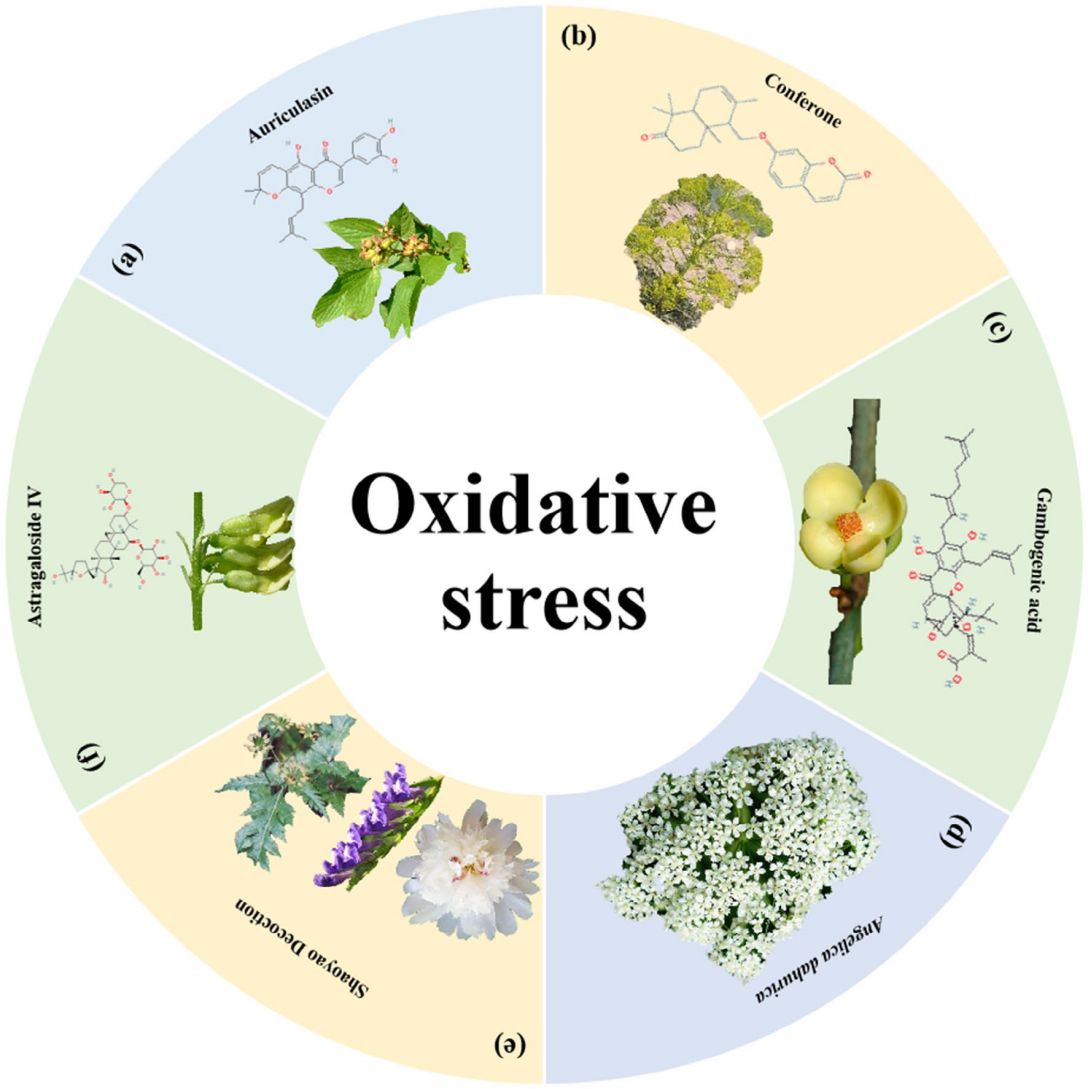
Herbal medicine treats CRC by regulating oxidative stress. (a) *Flemingia prostrata* Roxb. f. ex Roxb. (b) *Ferula fukanensis* K. M. Shen. (c) *Garcinia hanburyi* Hook.f. (d) *Angelica dahurica* (Fisch. ex Hoffm.) Benth. et Hook. f. (e) *Paeonia lactiflora* Pall., *Scutellaria baicalensis* Georgi and *Coptis chinensis* Franch. (f) *Astragalus membranaceus* (Fisch.) Bunge.

Furthermore, numerous phytomedicines have been shown to regulate oxidative stress to inhibit CRC growth, including granatin B, punicalagin, *imperata cylindrical* L. Raeusch (IMP), macrostemonoside A, auriculasin, conferone, gambogenic acid, *Angelica* dahurica, Shaoyao decoction and astragaloside IV [225, 226, 227, 228, 229, 230, 231, 232, 233, 234] (Figures 15 and 16).

### 2.11 Autophagy

Autophagy is a cellular degradation process observed in eukaryotic cells that plays a crucial role in preserving metabolic homeostasis by facilitating the breakdown of misfolded or aggregated proteins, as well as damaged organelles [235]. Autophagy exhibits a multifaceted and dual role in the progression of CRC, with its mechanisms differing based on tumour stage, microenvironment and genetic context. These mechanisms can be categorised into the following primary aspects: inhibiting early carcinogenesis and maintaining genomic stability [236]; promoting advanced tumour survival and metabolic adaptation [237]; regulating metastasis and drug resistance [238, 239]; and modulating the immune microenvironment [240]. In conclusion, autophagy exhibits spatiotemporal dynamics in CRC, and targeting autophagy should be integrated with tumour staging and molecular characteristics. For instance, inhibiting autophagy in advanced CRC may enhance sensitivity to chemotherapy, while early intervention may necessitate the activation of autophagy to prevent carcinogenesis.

**FIGURE 17:**
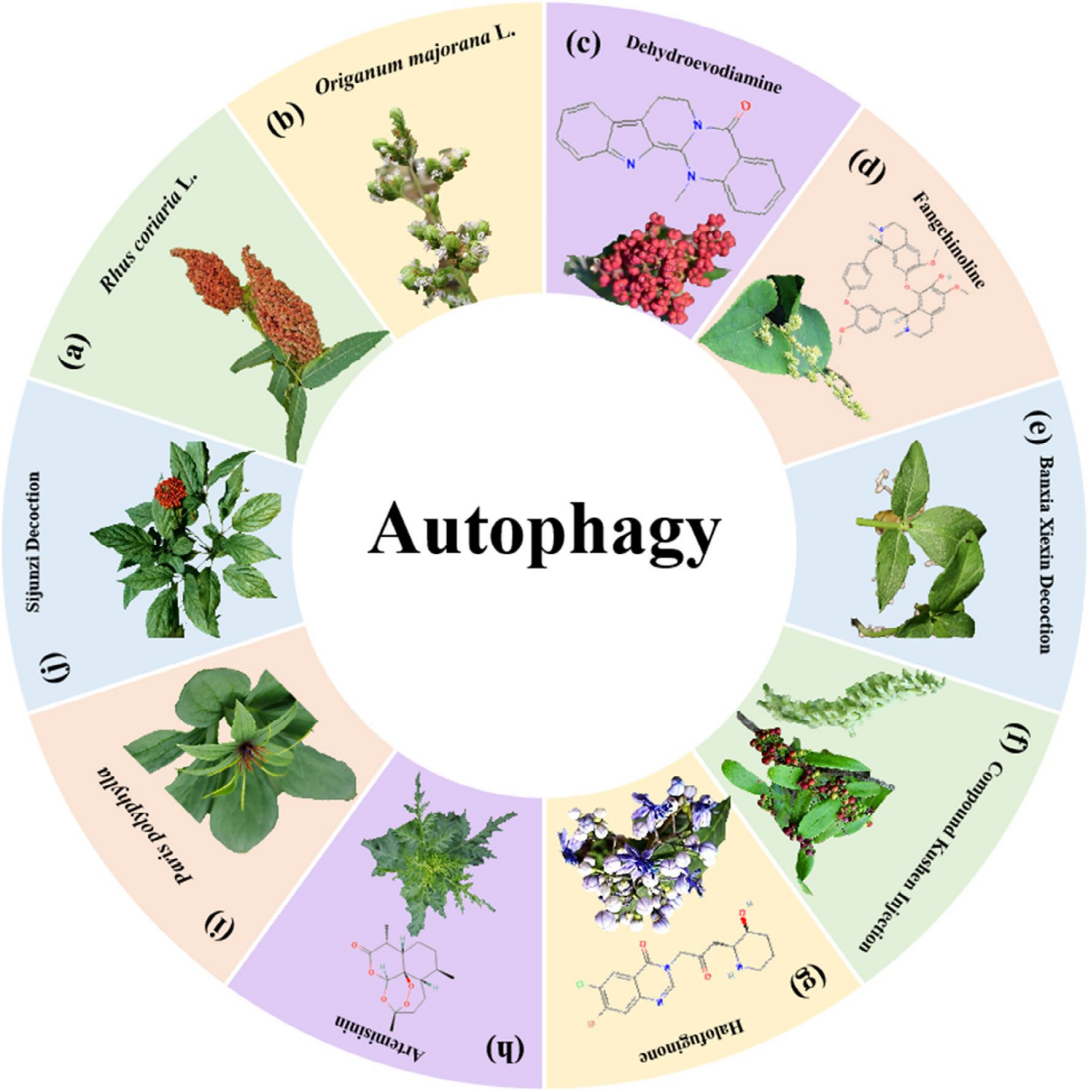
Herbal medicine treats CRC by regulating autophagy. (a) *Rhus coriaria* L. (b) *Origanum majorana* L. (c) *Tetradium ruticarpum* (A. Juss.) T. G. Hartley. (d) *Stephaniae tetrandine* S. Moore. (e) *Pinellia ternata* (Thunb.) Ten. ex Breitenb. (f) *Sophora flavescens* Ait. and *Smilax glabra* Roxb. (g) *Dichroa febrifuga* Lour. (h) *Artemisia annua* L. (i) *Paris polyphylla* Sm. (j) *Panax ginseng* C. A. Mey.

*Rhus coriaria* , commonly known as sumac, is a member of the Anacardiaceae family [241]. Research conducted by Ali Eid et al. has demonstrated that *Rhus coriaria* extract exhibited significant anti‐CRC properties, primarily through the stimulation of proteolysis and the induction of autophagic and apoptotic cell death, as evidenced by studies involving HT‐29 and Caco‐2 cells [241]. The resistance to 5‐fluorouracil is a critical factor that influences the effectiveness of treatments for CRC [242]. In their further investigations, Ali Eid et al. found that *Rhus coriaria* inhibited the viability, colony formation, and growth of HCT‐116‐WT and HCT‐116‐5FU‐R cells by triggering autophagic cell death. These findings suggest that *Rhus coriaria* may represent a promising and valuable source for the development of novel therapeutic agents for CRC [243]. *Origanum majorana* is an herbaceous plant found in southern Europe and the Mediterranean area. Research conducted by Ali Eid and colleagues has demonstrated that *Origanum majorana* ethanolic extract displayed significant anti‐proliferative effects on HT‐29 and Caco‐2 cells, primarily through the mechanisms of autophagy and apoptosis induction [244]. Furthermore, their studies indicated that the essential oil of *Origanum majorana* triggered p38 MAPK‐mediated autophagy, apoptosis and caspase‐dependent cleavage of P70S6K, thereby inhibiting cellular viability and colony growth in HT‐29 cells [245]. Dehydroevodiamine is a quinazoline alkaloid derived from *Tetradium ruticarpum* (A. Juss.) T. G. Hartley. A study conducted by Seung‐Heon Hong and colleagues demonstrated that dehydroevodiamine inhibited the cell viability of HCT116, CT26, SW480 and LoVo cells by inducing caspase‐dependent apoptosis and autophagy. In this study, dehydroevodiamine was also proved to inhibit the lung metastasis of CT26 cells in an in vivo model by regulating EMT [246]. Fangchinoline is an isoquinoline alkaloid isolated from *Stephaniae tetrandine* S. Moore (Menispermaceae). Feng et al. found that fangchinoline induced apoptosis in HT29 and HCT116 cells, and HT29 xenograft model by activating AMPK/mTOR/ULK1‐mediated autophagy [247]. Furthermore, numerous phytomedicines have been shown to regulate autophagy to inhibit CRC growth, including Banxia Xiexin decoction [248], compound Kushen injection [249], halofuginone and artemisinin [250], *Paris polyphylla* [251] and Sijunzi Decoction [252] (Figure 17).

## 3 CRC Metastasis

Metastasis remains the primary reason for mortality in individuals with solid tumours, such as CRC [253]. Despite significant advancements in our understanding of CRC, its metastasis continues to pose a considerable challenge, primarily due to limited treatment options and a poor prognosis [254]. Wogonin, a flavonoid derived from the herbal medicine *Scutellaria* baicalensis, has also been found to suppress the proliferation of SW620 and RKO cells and to inhibit EMT by downregulating AKT expression. The inhibitory effect of Wogonin on SW620 or HCT‐29 xenograft BALB/c nude mouse model is confirmed; however, its impact on EMT remains unclear [255]. Gentisic acid, a phenolic acid and a minor product of salicylic acid metabolism, has also been investigated. Feng et al. developed an animal model of lung metastasis by injecting MC38 cells into the tail vein. They showed that gentisic acid suppressed lung metastasis of CRC by impacting GPR81‐mediated DEPDC5 degradation [256]. Yiqi Huayu Jiedu Decoction, a TCM compound, is proven to inhibit liver metastasis by promoting the function of natural killer cells in a mouse model of CRC liver metastasis, which was prepared by injecting CT26‐GFP cells under the spleen envelope [257]. Echinacoside, a natural polyphenol compound, inhibits CRC liver metastasis by benefiting butyrate‐producing gut bacteria, thereby suppressing the PI3K/AKT signalling pathway and EMT in a mouse model of liver metastasis induced by intrasplenic injection [258]. Macelignan, a type of lignan isolated from *Myristica fragrans* , prevents CRC metastasis by inhibiting macrophage M2 polarisation through the ROS‐mediated PI3K/AKT pathway in a nude mouse model of CRC liver metastases [88]. Brusatol, a primary quassinoid extracted from *Bruceae* Fructus, suppresses the proliferation and lung metastasis of HCT116, HT29 and SW480 cells, as well as in the HCT116 and HT29 xenograft BALB/c nude mouse model, by activating ARRDC4 through modulation of the PI3K/YAP1/TAZ pathway [259]. *Astragalus mongholicus* Bunge‐ *Curcuma aromatica* Salisb. (AC), a TCM combination, is frequently employed to treat CRC. Tang et al. found that AC inhibited liver metastasis in CRC through modulating EMT by CXCL8/CXCR2 and PI3K/AKT/mTOR pathways [260, 261]. Yi Ai Fang, a TCM formula, influences the formation of vasculogenic mimicry in CRC by modulating HIF‐1α and EMT in HCT‐116 cells, as well as in an HCT‐116 xenograft nude mouse model. These findings suggest that Yi Ai Fang may possess the potential to inhibit tumour migration [262]. Ji et al. found that resveratrol suppressed lung metastases of LoVo cells in a mouse tail vein injection model, as well as lung and liver metastases in a mouse orthotopic transplantation tumour model. Resveratrol inhibited cell invasion by suppressing EMT through the TGF‐β1/Smads pathway, which regulated Snail/E‐cadherin expression [263]. Although many herbs have been shown to inhibit EMT, their anti‐metastatic effects require further validation through additional animal and human studies, such as lycorine [264], asperuloside [265], glabridin [266], ginsenoside Rb2 [267], physciosporin [268], fucoidan [269] and Danggui‐Sayuk‐Ga‐Osuyu‐Senggang‐Tang [270].

## 4 Clinical Applications

### 4.1 Curcumin

Curcumin is a flavonoid derived from *Curcuma longa* L. Y. Panahi and colleagues conducted a double‐blind, placebo‐controlled trial involving patients with stage 3 CRC aged ≥ 20 years who received postoperative chemotherapy. A total of 72 patients were randomly divided into curcuminoids capsules (500 mg/day) or placebo capsules, with 36 patients in each group, for 8 weeks. Although three subjects in the curcuminoids group and two in the placebo group were lost to follow‐up, the curcuminoids group showed significant improvements in serum C‐reactive protein levels (*p* = 0.002), erythrocyte sedimentation rate (*p* = 0.0001), function (*p* = 0.002) and overall quality of life (*p* = 0.020) [271]. Wang and colleagues conducted a clinical trial involving 126 patients with CRC who were randomly divided into curcumin capsule group (360 mg/day, three times/day) for 10–30 days (10–15 days: 12 cases, 16–20 days: 15 cases, 21–25 days: 18 cases and 26–30 days: 18 cases) and placebo capsule group before surgery [272]. The results of this study indicated that curcumin supplementation led to an increase in body weight among CRC patients, as well as an increase in p53 expression, DNA fragmentation and Bax levels, while Bcl‐2 expression decreased in CRC tissues [272]. MB‐6 is a botanical product composed of fermented soybean extract, green tea extract, *Antrodia camphorata* mycelia, spirulina, grape seed extract and curcumin extract. In a proof‐of‐concept clinical study, 72 patients with metastatic CRC received FOLFOX4 chemotherapy in combination with either MB‐6 (*n* = 34) or a placebo (*n* = 38) for 16 weeks. Although there were no notable differences between the MB‐6 and placebo groups in terms of the best overall response rate and overall survival, patients receiving MB‐6 exhibited a reduced rate of disease progression (0.0% vs. 15.8%, *p* = 0.026). Additionally, patients in the placebo group experienced a greater proportion of adverse events of at least grade 4 (28.9% vs. 2.9%, *p* = 0.004) and a higher incidence of increased serum creatinine levels (29% vs. 5.9%, *p* = 0.014) [273]. Because cancer patients need to take drugs for a long time, the safety of these drugs is of great concern. Multiple Phase I clinical trials of oral curcumin have shown that curcumin, whether administered alone or in combination with FOLFOX chemotherapy, is reliable in the effective dose range [274, 275, 276, 277].

### 4.2 Resveratrol

A clinical pharmacological investigation was conducted to evaluate the effects of resveratrol and its metabolites in CRC patients. The study involved 20 participants who ingested eight daily doses of resveratrol at dosages of either 0.5 g or 1.0 g prior to surgical resection. The findings indicated that although resveratrol could reduce tumour cell proliferation by 5%, this reduction was not statistically significant (*p* = 0.05). Furthermore, orally 0.5 g or 1.0 g resveratrol daily is adequate to elicit anti‐cancer effects in the human gastrointestinal tract [278]. Despite resveratrol having shown significant efficacy in animal models of CRC, its clinical effectiveness is significantly hindered by its low bioavailability. In order to address this limitation, Gescher and colleagues developed a micronised formulation of resveratrol and assessed its safety, pharmacokinetics and pharmacodynamics in CRC patients with hepatic metastases who were scheduled for hepatectomy (5.0 g daily for 14 days) [279]. Research indicated that plasma micronised resveratrol levels were 3.6‐fold higher than non‐micronised resveratrol and treatment with micronised resveratrol resulted in a significant 39% increase in cleaved caspase‐3 levels in malignant hepatic tissue. In summary, although resveratrol has shown remarkable efficacy in preclinical models of CRC, further clinical exploration is warranted to fully assess its potential clinical utility.

### 4.3 Berberine

Berberine is an alkaloid derived from *Coptis chinensis*. Approximately 90% of CRC cases arise from adenomatous lesions, particularly advanced colorectal adenomas [280]. A double‐blind, randomised, placebo‐controlled trial was performed involving patients aged 18 to 75 years who had undergone complete polypectomy within the preceding 6 months and presented with a minimum of one and a maximum of six colorectal adenomas. Participants were randomly assigned (1:1) to receive either berberine (0.3 g twice daily) or placebo tablets. In the two‐year observational study, it was observed that 36% of participants in the berberine group experienced recurrent adenomas, compared to 47% in the placebo group (unadjusted relative risk ratio for recurrence: 0.77, 95% CI 0.66–0.91; *p* = 0.001), without CRC development. The most frequently reported adverse event was constipation (1% in the berberine group, < 0.5% in the placebo group) without serious adverse events [280].

### 4.4 Fucoidan

Fucoidan is a sulfated polysaccharide that is naturally extracted from various species of brown, green and red seaweeds, predominantly composed of L‐fucose and sulfate groups [281]. A double‐blind, randomised, placebo‐controlled study was conducted involving 87 patients with locally advanced rectal cancer. Participants were randomly assigned to either the fucoidan group (*n* = 44) or the placebo group (*n* = 43). Those in the fucoidan group orally received 4 g of low‐molecular‐weight fucoidan (twice a day, 3 months). This fucoidan was obtained through enzyme hydrolysis of the original substance, and participants also underwent neoadjuvant concurrent chemoradiotherapy. In contrast, participants in the placebo group orally received 4 g of cellulose powder (twice a day, 3 months). Although enhanced physical well‐being was observed at 2 and 3 months (both *p* < 0.0125), along with improvements in skin rash, itching and fatigue (both *p* < 0.05) and an increase in the abundance of the genus *Parabacteroides* in the gut microbiota in the fucoidan group, the improvements in the functional assessment of cancer therapy for patients with CRC were not statistically significant [282]. However, a prospective, randomised, double‐blind, controlled trial conducted by the same research team confirmed the efficacy of low‐molecular‐weight fucoidan as a chemotherapy‐targeted drug supplement in patients with metastatic CRC [283]. In this clinical trial, 54 patients with metastatic CRC were randomly assigned to either the fucoidan group (*n* = 28) or the Control group (*n* = 26). Participants in the fucoidan group orally received 4 g of low‐molecular‐weight fucoidan (twice a day, 6 months). In contrast, participants in the Control group orally received 4 g of cellulose powder (twice a day, 6 months). All patients underwent chemotherapy with targeted therapy. The disease control rates for the primary endpoint were 92.8% in the fucoidan group and 69.2% in the Control group, respectively (*p* = 0.026). However, secondary endpoints, including the overall response rate, progression‐free survival, overall survival, adverse effects and quality of life, were not statistically significant. In summary, the studies indicate that low‐molecular‐weight fucoidan exhibits varying effects in different disease states of CRC, highlighting the need for additional clinical data to confirm its clinical efficacy.

### 4.5 Epigallocatechin‐3‐Gallate

Epigallocatechin 3‐gallate is one of the most important and abundant polyphenols found in green tea [284]. In order to examine the potential biological preventive effects of flavonoids, a study was conducted to assess the recurrence risk of neoplasia in patients who had undergone resection for CRC [285]. A total of 36 patients were included in the study, all of whom had resected colon cancer. The participants were categorised into two groups: one group received a flavonoid mixture consisting of a daily dose of 20 mg of apigenin and 20 mg of epigallocatechin‐3‐gallate (*n* = 14), while the other group served as a matched control (*n* = 22) for a duration of 3–4 years. A total of 29 patients underwent surveillance colonoscopy. Among those who received flavonoid treatment, there was no recurrence of cancer, and only one adenoma was identified. In contrast, the control group experienced three instances of cancer recurrence and four adenomas. The overall recurrence rate for neoplasia was 7% (1 out of 14) in the treated cohort, compared to 47% (7 out of 15) in the control group, with a statistically significant difference observed (*p* = 0.027).

CRC typically follows an ‘adenoma‐cancer’ sequence, in which colorectal adenomas represent the stage preceding the occurrence of CRC. Colorectal adenomas are benign tumours that originate from the glandular epithelium of the colorectal mucosa and are the most prevalent precancerous lesions associated with CRC, accounting for approximately 85% to 90% of all cases. A randomised clinical trial was conducted involving 176 patients who underwent complete resection of colorectal adenomas through endoscopic polypectomy [286]. The participants were randomly assigned to one of two groups: the supplementation group (0.9 g of green tea extract tablets daily for 12 months) or the control group without green tea extract tablets. Each green tea extract tablet (500 mg) contained 225 mg of green tea extract, comprising 51.5 mg of (−)‐epigallocatechin‐3‐gallate, 11.6 mg of (−)‐epicatechin, 65.5 mg of (−)‐epigallocatechin, 5.7 mg of (−)‐epicatechin gallate and 10.9 mg of caffeine. A follow‐up colonoscopy was performed 12 months later on 143 patients (71 in the control group and 72 in the green tea extract tablet group). The incidence of metachronous adenomas was found to be 42.3% (30 out of 71) in the control group, compared to 23.6% (17 out of 72) in the green tea extract tablet group (relative risk, 0.56; 95% confidence interval, 0.34–0.92). Additionally, the mean number of relapsed adenomas was significantly lower in the green tea extract tablet group than in the control group (0.7 ± 1.1 vs. 0.3 ± 0.6, *p* = 0.010). However, no significant differences were observed between the two groups regarding body mass index, dietary intakes, serum lipid profiles, fasting serum glucose levels and serum C‐reactive protein levels (*p* > 0.05).

Aberrant crypt foci (ACF) are the earliest morphologically detectable lesions in the colorectal mucosa and are believed to be precursors to adenomas and cancers [287]. Stephen Sontag and colleagues conducted a randomised phase II trial evaluating polyphenon E (Poly E) in patients at high risk of recurrent colonic neoplasia. A total of 39 participants with a history of colorectal adenomas or cancers, who exhibited five or more rectal aberrant crypt foci (ACF), were assigned to either the Poly E group oral Poly E (780 mg of epigallocatechin‐3‐gallate) or a placebo group. Of these, 32 out of 39 participants successfully completed the 6‐month treatment period. Poly E demonstrated a favourable tolerance profile and exhibited no significant toxicity at the doses utilised in the study. However, it did not demonstrate a statistically significant reduction in the number of rectal ACF when compared to the placebo group [287].

### 4.6 Quxie Capsule

Quxie capsule, consisting of *Croton tiglium* , *Evodia* rutaecarpa, *Rhizoma* zingiberis, *Cinnamomum cassia* Presl, *Radix* aconiti, *Pinellia ternata* and *Pericarpium citri* Reticulatae, is an herbal formulation that has been utilised for the treatment of CRC for more than 10 years. A block‐randomised, double‐blind, placebo‐controlled trial involving 60 patients with metastatic CRC was conducted by YANG and colleagues. Participants were assigned to either the Quxie capsule group or the Control group. The patients assigned to the Quxie capsule group underwent standard treatment protocols, which encompassed chemotherapy, radiotherapy, targeted therapy and supportive care. Additionally, they received Chinese herbal medicine in conjunction with the Quxie capsule (50 mg/kg twice daily from day 1 to day 20, over a 30‐day treatment cycle) for a duration of 3 months. The participants in the control group were administered standard therapeutic interventions alongside Chinese herbal medicine in conjunction with a placebo for a duration of 3 months. The findings of this study showed that Quxie capsule significantly increased the median overall survival to 23.9 months, compared to 14.3 months in the Control group (*p* < 0.05), without significant differences between the two groups in progression‐free survival [288]. YANG and colleagues also investigated the safety of the Quxie capsule in patients with metastatic CRC, and the results showed no severe haematological toxicity or liver and renal function injury [289]. In summary, Quxie capsule prolongs the overall survival of patients with metastatic CRC without serious complications.

### 4.7 Jianpi Jiedu Formula

Over the past few years, TCM has received more recognition for its effectiveness and safety in treating CRC. Many Chinese patients with CRC seek assistance from TCM during or after first‐line treatment [290]. Jianpi Jiedu formula is a TCM formula, consisting of *Radix* Astragali, *Radix* Ginseng, *Rhizoma Atractylodis* Macrocephalae, Poria, *Semen* Coicis, *Rhizoma* Smilacis Chinensis, *Herba Hedyotidis* Diffusae, *Herba Scutellariae* Barbatae, *Rhizoma* Paridis and *Radix Actinidiae* Chinensis. In comparison to the chemotherapy group (*n* = 295), the Jianpi Jiedu formula combined with chemotherapy prolonged the mean survival time by 5.594 months and the median survival time by 6 months for patients with stage II and III CRC (*n* = 171, *p* = 0.004) [291]. However, the study was a retrospective clinical investigation rather than a controlled trial and it lacked a placebo control group. Consequently, the clinical efficacy of the Jianpi Jiedu formula on CRC still requires validation through additional controlled clinical studies.

### 4.8 Hezhong Granules

Hezhong granules, consisting of *Pinellia ternata* (*Thunb*.) Makino, *Zingiber officinale* Roscoe, *Scutellaria baicalensis* Georgi, *Coptis chinensis* Franch., *Evodia rutaecarpa* (*Juss*.) Benth, *Panax ginseng* C. A. Mey, *Poria cocos* (Schw.) Wolf and *Cinnamomum verum* J. Presl, is an herbal formulation that has been utilised to treat CRC. A prospective randomised controlled trial enrolling 112 patients with advanced CRC was carried out from October 2020 to February 2022 across 12 hospitals in southwestern China. Compared to patients in the placebo group (a 5‐HT3‐receptor antagonist, dexamethasone and a placebo), the incidence of chemotherapy‐induced nausea and vomiting was significantly lower in those from the Hezhong granules group (a 5‐HT3‐receptor antagonist, dexamethasone and Hezhong granules). Furthermore, Hezhong granules did not cause serious adverse events, indicating that they are both effective and well‐tolerated for preventing nausea and vomiting caused by chemotherapy in patients with advanced CRC [292].

### 4.9 Shenbai Granules

Shenbai granules, consisting of *Sophorae flavescentis* radix, *Hedyotis* diffusa, *Codonopsis* radix, *Atractylodis macrocephalae* rhizoma, *Coicis* semen, *Coptidis* rhizoma, *Mume* fructus and *Zingiberis rhizoma* praeparatum, have been utilised for the management of CRC for several years. Cheng and colleagues conducted a multicentre, randomised, double‐blind, placebo‐controlled clinical trial with a follow‐up period of 2 years, involving 400 patients with adenomas who had undergone complete polypectomy within the preceding 6 months. The results of this study demonstrated that Shenbai granules significantly decreased the recurrence rate of adenomas. This was evidenced by notable differences between the Shenbai granules group and the placebo group regarding the proportion of patients experiencing at least one recurrent adenoma (42.5% vs. 58.6%; OR, 0.47; 95% CI, 0.29–0.74; *p* = 0.001) and sessile serrated lesion (1.8% vs. 8.3%; OR, 0.20; 95% CI, 0.06–0.72; *p* = 0.01). However, Shenbai granules had no significant effect on the proportion of patients who developed polypoid lesions or high‐risk adenomas [293].

Although recent years have witnessed an increasing number of herbal medicines and their active ingredients demonstrated to have therapeutic effects on CRC, these remedies are primarily regarded as important adjuvant therapies rather than primary treatments. However, many clinical studies conducted to date are retrospective rather than randomised controlled trials, indicating that numerous research findings require validation through higher‐quality and rigorously designed randomised controlled trials. It is hoped that the discovery of the anti‐tumour efficacy of paclitaxel, along with its widespread clinical application, will reinforce our belief in the significant developmental potential of herbal medicines.

## 5 Reproducibility Analysis

The pertinent literature for the last 5 years were gathered from several databases such as PubMed, Google Scholar, Medline, ScienceDirect, Springer Link and Web of Science, using the following keywords: ‘phytomedicine’, ‘botanical drug’, ‘herbal medicine’, ‘natural medicine’, ‘natural drug’ and ‘colorectal cancer’. Non‐experimental articles, such as reviews and meta‐analyses, were excluded from this study (Table 1). In Table 2, we present the frequency of different botanicals reported for the treatment of CRC. Due to the extensive number of references, only the frequency of reported botanicals is provided (see Table S1 for details on specific references). Curcumin, resveratrol, berberine, shikonin, dihydroartemisinin, fucoidan, luteolin, andrographolide, piperine, kaempferol, emodin, cannabidiol, tanshinone IIA and evodiamine have consistently been reported to exhibit therapeutic effects on CRC. These compounds may hold significant potential for the development of future anti‐CRC drugs or precursor agents.

**TABLE 1:** The efficacy and mechanism of herbal medicine in the treatment of CRC.

Herbal medicine	Plant origin	Model	Effects	Molecular mechanisms	References
Wumei Wan	*Prunus mume* (Siebold) Siebold & Zucc., *Coptis chinensis* Franch., *Neolitsea cassia* (L.) Kosterm., *Codonopsis pilosula* (Franch.) Nannf., *Zanthoxylum bungeanum* Maxim., *Zingiber officinale* Roscoe, *Aconitum carmichaelii* Debeaux, *Phellodendron amurense* Rupr., *Asarum heterotropoides* F. Schmidt and *Angelica sinensis* (Oliv.) Diels.	● AOM/DSS‐induced CAC mouse model (C57BL/6J)	● Inhibits tumour growth	● Suppresses intestinal inflammation (IL‐1β, IL‐6 and TNF‐α) ● Suppresses myeloid‐derived suppressor cells and increase CD4^+^ T and CD8^+^ T cells in spleen	[10]
Tetra‐ and pentahydroxyflavanones	*Scutellaria baicalensis* Georgi.	● AOM/DSS‐induced CAC mouse model (C57BL/6J)	● Inhibits tumour growth	● Suppresses IL‐10 and PD‐1 through inhibiting COX‐2 and CD8^+^ T‐cell exhaustion by TOX/TOX2	[11]
Huang Qin decoction	*S*. *baicalensis* , *P*. *lactiflora* , *G*. *uralensis* and *Z*. *jujuba*	● AOM/DSS‐induced CAC mouse model (C57BL/6J)	● Inhibits tumour growth	● Suppresses intestinal inflammation (TNF‐α and IL‐1β) ● Neutrophil infiltration in colon ● Restores intestinal mucosal permeability (occludin and ZO‐1) ● Improves the immunosurveillance of CD8^+^ T cells ● Modulates PAD4‐dependent neutrophil extracellular traps	[12]
Evening primrose root extract and oenothein B	Evening primrose ( *Oenothera biennis* )	● Co‐culture system using human PD‐L1‐expressed murine MC38 cells and CD8^+^ tumour‐infiltrating T lymphocytes expressing humanised PD‐1 ● CRC C57BL/6J mouse model bearing MC38 cells expressing humanised PD‐L1 and PD‐1 proteins	● Inhibits tumour growth	● Suppresses the binding between PD‐L1 and PD‐1 to increase CD8^+^ T cell‐mediated tumour cytotoxicity	[14]
Marsdenia tenacissima tablet	*Marsdenia tenacissima* (Roxb.) Wight et Arn.	● CRC patients ● HCT116 and LoVo cells ● AOM/DSS‐induced CAC mouse model (BALB/c) ● Subcutaneous CT26 tumour model (BALB/c)	● Inhibits tumour growth ● Inhibits the immune escape of cancer cells	● Increases the number of CD3^+^/CD8^+^ tumour‐infiltrating T cells ● Suppresses TGF‐β1 and PD‐L1 expressions	[15]
Triterpenoids of *Rhus chinensis*	*Rhus chinensis* Mill.	● DLD1 and CT26 cells ● Subcutaneous CT26 tumour model (BALB/c)	● Inhibits tumour growth	● Prevents CD8^+^ T‐cell dysfunction by enhancing mTOR and glycolytic gene expression	[16]
Honeysuckle‐derived microRNA2911	*Lonicerae Japonicae* Flos	● CT26 tumour‐bearing C57BL/6, Sidt1^+/+^ and Sidt1^−/−^ mouse models	● Inhibits tumour growth	● Increases T lymphocytes (CD4^+^ T cells and CD8^+^ T cells) infiltration ● Suppresses TGF‐β1 expression	[17]
*Patrinia villosa* aqueous extract	*Patrinia villosa* Juss.	● HCT116, SW480, colon 26‐luc cells ● Xenograft HCT116 tumour model (nude mice) ● Zebrafish model	● Inhibits tumour growth ● Inhibits tumour metastasis	● Regulates TGF‐β R1‐smad2/3‐E‐cadherin and FAK‐RhoA‐cofilin pathways ● Increases helper T (CD3^+^CD4^+^) and cytotoxic T lymphocytes (CD3^+^CD8^+^) percentage ● Increases the relative abundance of gut microbiota	[18]
Turmeric extract, with absorbable curcumin	*Curcuma longa* L.	● HCT116, HT‐29 and colon 26, colon 26‐M01 cells ● Orthotopic xenografts colon 26‐M01 tumour model (BALB/c)	● Inhibits tumour growth ● Inhibits tumour metastasis (liver and lung)	● Regulates cofilin, FAK/pSrc, AKT, Erk and STAT3 ● Increases CD4^+^ helper T lymphocytes in thymus and CD8^+^ cytotoxic T lymphocytes in tumour tissue	[20]
Gegen Qinlian decoction	*Radix* Puerariae, *Scutellariae* Radix, *Coptidis* Rhizoma and *liquorice*	● Xenograft CT26 tumour model (BALB/c)	● Inhibits tumour growth	● Increases the proportion of CD8^+^ T cells in peripheral blood and tumour tissues ● Remodels gut microbiota	[21]
Lentinan	*Lentinus edodes* (Berk.) sing.	● Xenograft CT26 tumour model (BALB/c) ● AOM/DSS‐induced CAC mouse model (BALB/c)	● Inhibits tumour growth	● Promotes dendritic cell maturation ● Polarises tumour‐associated macrophage from a pro‐tumorigenic M2 to an anti‐tumorigenic M1 phenotype (CD11b^+^CD80^+^)	[27]
		● Xenograft CT26 tumour model (BALB/c) ● HCT26 cells	● Inhibits tumour growth	● Promotes tumour infiltration of CD4^+^, CD8^+^, NK1.1^+^ cells, monocytes and neutrophils ● Suppresses tumour‐associated macrophages	[28]
Qizhen decoction	*Astragali* Radix, *Ligustri Lucidi* Fructus, *Codonopsis* Radix, *Salviae Miltiorrhizae Radix* Et Rhizoma, *Radix Paeoniae* Alba, *Herba Hedyotidis* Diffusae, *Polygoni Cuspidati Rhizoma* Et Radix, *Fiveleaf Gynostemma* Herb	● AOM/DSS‐induced CRC mouse model (C57BL/6J)	● Inhibits tumour growth	● Promotes the maturation of dendritic cells to release IL‐12 and activate the JAK2/STAT4 pathway to induce effector T‐cell activation by increasing the abundance of Akkermansia	[30]
Tong‐Xie‐Yao‐Fang	*Atractylodes macrocephala* Koidz., *Paeonia lactiflora* Pall., *Citrus reticulata* Blanco, *Saposhnikovia divaricate* (Turcz.) Schischk	● Mouse model of chronic stress by chronic restraint stress and subcutaneous injection of CT26‐Luc cells (BALB/c)	● Inhibits tumour growth	● Suppresses the HPA axis ● Facilitates dendritic cells maturation, thereby triggering T‐cell‐mediated immune response, evidenced by increased CD4+ T cells, CD4+/CD8+ T cells and Th1 cells percentage ● Promotes serum IFN‐γ, IL‐18, IL‐2 and IL‐12 levels ● Suppresses serum IL‐4 and IL‐10 levels	[31]
Curcumin	*Curcumae Longae* Rhizoma	● SW480 cells	● Inhibits tumour growth	● Suppresses early apoptosis and arrested ● Suppresses cell cycle in G2/M phase via increasing cyclin B1 ● Suppresses Th17 cell differentiation	[32]
Periploca sepium periplosides	*Cortex Periplocae*	● DSS/AOM induced‐CAC model (C57BL/6)	● Inhibits tumour growth	● Modulates the gut microbiota structure ● Suppresses pathogenic Th17 cell population	[33]
Ginseng berry	*Panax ginseng* C.A. Meyer	● HCT116 and HT29 cells	● Inhibits tumour growth	● Suppresses Th17 cell differentiation and thus regulates the balance of Th17/Treg	[34]
Yi‐Yi‐Fu‐Zi‐Bai‐Jiang‐San	*Semen Coicis*, *monkshood* and *Herba Patriniae*	● *Apc* ^Min/+^ mice (C57BL/6J)	● Inhibits tumour growth	● Regulates gut flora, including *Bacteroides fragilis* and *Lachnospiraceae* ● Suppresses CD4^+^CD25^+^Foxp3^+^ Tregs by regulating gut microbiota	[41]
		● MC‐38 and IEC‐6 cells ● AOM/DSS‐induced CRC mice (C57BL/6J)	● Inhibits tumour growth	● Promotes Tregs‐induced immunosuppression through HIF‐1α‐mediated hypoxia	[42]
Quxie capsule	*Evodiae fructus*, *Zingiberis rhizoma*, *Cortex cinnamomi*, *Radix aconiti*, *Coptis chinensis*, *Pinelliae rhizoma*, *Citri grandis exocarpium*, *Poria*, *Arecae semen*, *Magnoliae officnalis*, *Aurantii fructus immaturus*, *Acori tatarinowii rhizoma*, *Corydalis rhizoma*, *Panax Ginseng* , *Lignum aquilariae resinatum*, *Radix platycodonis*, *Succinum*, *Crotonis fructus*, *Galli Gigerii endothelium corneum*, *Hordei fructus germinatus* and *Gleditsiae fructus abnormalis*	● Xenograft HCT26 tumour model ● HCT26 and HCT116 cells	● Inhibits tumour growth	● Increases the ratios of Th1/Th2 and Th17/Treg cells ● Suppresses the level of Foxp3 (the key regulator of Treg cells)	[43]
Rubiginosin B	*Rhododendron brachypodum* (R. brachypodum)	● CT26 and MC38 xenograft tumour model	● Inhibits tumour growth	● Suppresses TGFβ‐induced CD4^+^Foxp3^+^ regulatory T cells differentiation	[44]
Cyasterone	*Ajuga decumbens* Thunb (Labiatae) or *Cyathula officinalis* Kuan	● *BRAF* ^*V600E*^‐mutant mouse model with CRC	● Inhibits tumour growth	● Enhances the diversity of the gut microbiota ● Elevates the abundance of beneficial bacteria, including *Prevotellaceae*, *Muribaculaceae* and *Ruminococcaceae*	[47]
Huangqin decoction	*Scutellaria baicalensis* Georgi, *Paeonia lactiflora* Pall, *Ziziphus jujuba* Mill and *Glycyrrhiza uralensis* Fisch	● AOM/DSS‐induced CRC mice (C57BL/6J) ● SW480 and HT‐29 cells	● Inhibits tumour growth	● Microbial butyrate mediated PI3K/AKT Pathway suppression	[48]
		● Deoxycholic acid‐induced *Apc* ^Min/+^ mice	● Inhibits tumour growth	● Increases the abundance of *Lachnospiraceae*, *Firmicutes*, *Fusobacteria* and *Clostridium* ● Suppresses the abundance of *Eggerthellales*	[49]
Xiaoyaosan	*Angelica* sinensis, *Paeonia lactiflora* , *Bupleuri* Radix, *Atractylodis Macrocephalae* Rhizoma, *Glycyrrhizae* Radix, Poria, *Zingiberis Rhizoma* Recens, *Menthae Haplocalycis* Herba	● CRC xenograft model in mice with chronic restraint stress (athymic nude mice)	● Inhibits tumour growth	● Regulates the abundance of *Bacteroides*, *Lactobacillus*, *Desulfovibrio* and *Rikenellaceae*	[50]
Xiao‐Chai‐Hu‐Tang	*Bupleuri* Radix, *Scutellariae* Radix, *Ginseng Radix* et Rhizoma, *Pinelliae* Rhizoma, *Glycyrrhizae Radix* et Rhizoma, *Zingiberis Rhizoma* Recens and *Jujubae* Fructus	● CRC patients ● MC38 xenograft model in mice under depression (C57BL/6J)	● Inhibits tumour growth	● Regulates gut microbiota‐mediated TLR4/MyD88/NF‐κB pathway	[51]
San‐Wu‐Huang‐Qin decoction	*Scutellariae* Radix, *Sophorae Flavescentis* Radix and *Rehmanniae* Radix	● AOM/DSS‐induced CRC mouse model (C57BL/6J)	● Inhibits tumour growth	● Improves mucosal barrier partially by targeting gut microbiota: *Escherichia*‐*Shigella*	[52]
Berberine	*Coptis chinensis*	● AOM/DSS‐induced CRC mouse model (C57BL/6) ● HT‐29 cells	● Inhibits tumour growth	● Suppresses Hedgehog pathway ● Modulates gut microbiota	[53]
Xianlian Jiedu decoction	*Agrimoniae* Herba, *Coptidis* Rhizoma, *Sophorae Flavescentis* Radix, Coicis Semen, Sparg*a*nii Rhizoma, *Curcumae* Rhizoma, *Astragali* Radix and *Atractylodis Macrocephalae* Rhizoma	● AOM/DSS‐induced CRC mouse model (C57BL/6)	● Inhibits tumour growth	● Improves gut microbiota disorders and associated short‐chain fatty acids, sphingolipid and glycerophospholipid levels by inhibiting the abundance of *Turicibacter* and *Clostridium*_*sensu*_*stricto*_*1*	[64]
Modified Shenlingbaiaizhu decoction	*Panax ginseng* C. A. Mey, *Wolfiporia cocos* (F.A. Wolf) Ryvarden & Gilb, *Wikstroemia* indica (L.) C. A. Mey, *Atractylodes macrocephala* Koidz, *Ficus hirta* Vahl, *Polygonum chinense* L, *Kalopanax septemlobus* (Thunb.) Koidz, *Curcuma zedoaria* (Christm) Roscoe, *Coix lacryma*‐jobi L, *Ailanthus altissima* (Mill.) Swingle	● Orthotopic tumour ● SW480 and MC38 cells	● Inhibits tumour growth	● Suppresses the pluripotency of colorectal cancer stem cells by inhibiting TGF‐β mediated EMT program	[69]
Pien Tze Huang	*Moschus*, *Calculus Bovis*, *Snake Gall* and *Radix Notoginseng*	● HT‐29 SP cells	● Inhibits cell proliferation	● Suppresses stem‐like side population	[70]
		● SW480 cells	● Inhibits cell proliferation	● Suppresses stem‐like side population ● Suppresses the Notch1 signalling pathway	[71]
		● HCT116, SW480 and HCT15 cells ● MC38 xenograft tumour mouse model (C57BL/6) ● CRC patient‐derived organoids	● Inhibits tumour growth	● Promotes T cell‐mediated killing of CRC by inhibiting stemness and PD‐L1 expression	[72]
Phenethyl isothiocyanate	Cruciferous vegetables	● HCT116 cells ● HCT116 xenograft tumour mouse model	● Inhibits tumour growth	● Suppresses cancer stem cell properties	[73]
		● DLD‐1 and SW480 cells	● Inhibits cell proliferation	● Suppresses cancer stem cell properties by inhibiting the Wnt/β‐catenin signalling pathway	[74]
Vitexin	*Crataegus pinnatifida* Bge. and *Vigna radiata* (L.) Wilczek	● AOM/DSS‐induced CRC mouse model (BALB/c)	● Inhibits tumour growth	● Increases M1 macrophage polarisation in colonic tumour tissue ● Suppresses M1 macrophage polarisation in adjacent noncancerous tissues	[83]
		● THP‐1, RAW 264.7, CT26. WT and HCT 116 cells ● AOM/DSS‐induced CRC mouse model (C57BL/6)	● Inhibits the transition from chronic colitis to colorectal cancer	● Targets macrophage polarisation via the VDR/PBLD signalling pathway	[84]
Jiedu Xiaozheng Yin	*Hedyotis diffusa* Willd, *Spica* prunellae, *Pseudobulbus* Cremastrae and *Sophora Flavescens*	● AOM/DSS‐induced CRC mouse model (BALB/c) ● RAW264.7 cells	● Inhibits tumour growth	● Increases M1 macrophage polarisation through TLR4 pathway in colonic tumour tissue ● Suppresses M2 macrophage polarisation in colonic tumour tissue	[85]
Dictamnine	*Dictamnus dasycarpus* Turcz.	● DLD‐1 xenograft mouse model (BALB/c nude mice) ● DLD‐1 and LoVo cells ● THP‐1 cells	● Inhibits tumour growth	● Increases ferroptosis and suppressing M2 macrophage polarisation via the MAPK signalling	[86]
Triterpenoids of *Rhus chinensis* Mill.	*Rhus chinensis Mill*	● SW620 and HCT116 cells	● Inhibits cell growth and invasion	● Inhibits the ASIC2‐induced calcineurin/NFAT pathway ● Suppresses glycolysis	[95]
		● SW620 cells	● Inhibits cell proliferation	● Suppresses glycolysis and glutaminolysis	[96]
		● Co‐culture CD8^+^ T cells and CT26 cells	● Inhibits cell proliferation	● Promotes glycolysis in CD8^+^ T‐cells	[16]
Atractylenolide I	*Atractylodis Macrocephalae* Rhizoma	● HCT116, COLO205, SW480 and LOVO cells ● HCT116 xenograft tumour mouse model (Balb/c‐nu/nu nude mice)	● Inhibits tumour growth	● Suppresses glycolysis ● Suppresses AKT/mTOR pathway ● Suppresses stemness maintenance	[76]
		● HCT116 and SW480 cells ● HCT116 xenograft tumour mouse model (nude mice)	● Inhibits tumour growth	● Promotes apoptosis ● Suppresses glycolysis ● Suppresses the JAK2/STAT3 pathway	[97]
Saponin monomer 13 of the dwarf lilyturf tuber (DT‐13)	*Liriopes* Radix	● HCT‐15, HCT‐116, COLO205, HT‐29, SW620 and SW480 cells ● Orthotopic mouse model (HCT‐15, BALB/c nude mice)	● Inhibits tumour growth	● Suppresses glycolysis ● Activates AMPK and inhibited m‐TOR	[98]
Ginsenoside Rh3	Hot‐processed ginseng	● HT29, HCT116, SW620, DLD1 and RKO cells ● HT29 and HCT116 xenograft mouse model (BALB/c nude mouse)	● Inhibits tumour growth	● Activates pyroptosis and ferroptosis ● Regulates the Stat3/p53/NRF2 axis	[113]
Solanine	*Solanum nigrum* L	● HCT116 and SW480 cells	● Inhibits cell proliferation	● Activates ferroptosis by increasing ALOX12B/ADCY4	[115]
Curcumin	*Curcumae Longae* Rhizoma	● SW480 cells	● Inhibits cell proliferation	● Activates JNK‐mediated ferroptosis	[116]
		● HCT‐8 cells	● Inhibits cell proliferation	● Activates ferroptosis by inhibiting PI3K/AKT/mTOR Signalling	[118]
		● SW620 cells and LoVo ● Xenograft SW620 tumour (BALB/c nude mouse)	● Inhibits tumour cell growth	● Activates ferroptosis by regulating p53 and solute carrier family 7 member 11/glutathione/glutathione peroxidase 4 signalling axis	[119]
JianPi JieDu Recipe	*Radix* Astragal, *Rhizoma Atractylodis* Macrocephala, wild grapevines, *Fructus* Akebia, *Salvia chinensis* Benth. and *Evodia* rutaecarpa.	● LoVo, HCT116 and MC‐38 cells ● Liver metastasis model (MC38 cells on C57BL/6 mouse)	● Inhibits tumour growth ● Inhibits colorectal cancer liver metastasis	● Regulates ITGBL1‐rich extracellular vesicles‐mediated activation of cancer‐associated fibroblasts	[136]
Pennogenin 3‐O‐beta‐chacotrioside and polyphyllin VI	*Paris polyphylla*	● Caco‐2 and HT‐29 cells	● Inhibits CRC cell invasion	● Inhibits the release of extracellular vesicles from *Fusobacterium nucleatum*	[142]
Coptisine	*Coptis chinensis* Franch.	● HCT‐116 cells ● HCT‐116 xenograft mouse model (BALB/c nude mouse)	● Inhibits tumour growth	● Inhibits the PI3K/AKT pathway ● Activates mitochondrial‐associated apoptosis	[149]
Tanshinone IIA	*Salvia miltiorrhiza* Bunge	● SW837 and SW480 cells	● Inhibits cell proliferation	● Increases mitochondrial fission by activating JNK‐Mff signalling pathways	[153]
		● SW480 cells	● Inhibits cell proliferation	● Activates INF2‐mediated mitochondrial fission ● Activates Mst1‐Hippo pathway	[154]
Piperine	Black ( *Piper nigrum* ) and long pepper ( *Piper longum* )	● HT‐29 cells ● CT26 xenograft tumour mouse model (BALB/c)	● Inhibits tumour growth	● Synergistically with Celecoxib to inhibit CRC cell proliferation via modulating Wnt/β‐catenin signalling pathway	[155]
Curcumin	*Curcuma longa* L.	● SW480 and HCT116 cells	● Inhibits tumour cell growth	● Increases apoptosis ● Activates NLRP3 inflammasome‐dependent pyroptosis	[175]
Kanglaite (KLT) injection	*Coix lacryma*‐*jobi* (adlay) seed	● HCT106, HCT116, LoVo and CT26 cells ● CT26 xenograft tumour mouse model (BALB/c)	● Inhibits tumour growth	● Suppresses NF‐κΒ ● Promotes connexin 43	[180]
		● HCT106, HCT116, LoVo and CT26 cells ● CT26 xenograft tumour mouse model (BALB/c)	● Inhibits tumour growth	● Suppresses NF‐κΒ	[181]
Evodiamine	*Tetradium ruticarpum* (A. Juss.) T. G. Hartley	● HCT‐116, HCT‐116/L‐OHP cells ● HCT‐116/L‐OHP xenograft tumour mouse model (athymic nude mice)	● Inhibits tumour growth	● Suppresses the p50/p65 NF‐κB pathway	[182]
		● HCT‐116 cells	● Inhibits tumour growth	● Suppresses the JAK2/STAT3 pathway	[183]
Scutellarin	*Scutellaria altissima* L.	● SW480 cells ● AOM/DSS‐induced CRC mouse model (C57BL/6)	● Inhibits tumour growth	● Suppresses Hedgehog pathway and NF‐κB‐mediated inflammation	[188]
		● HT‐29 cells ● AOM/DSS‐induced CRC mouse model (C57BL/6)	● Inhibits tumour growth	● Suppresses serum TNF‐α and IL‐6 levels ● Suppresses inhibiting the Wnt/β‐catenin pathway	[189]
Triterpenes of jujube	*Ziziphus jujuba* Mill.	● AOM/DSS‐induced CRC mouse model (C57BL/6)	● Inhibits tumour growth	● Suppresses the PI3K/AKT/NF‐κB pathway	[190]
Pristimerin	*Celastraceae* and *Hippocrateaceae* families	● HCT‐116 cells ● HCT‐116 xenograft mouse model (BALB/c nude mouse)	● Inhibits tumour growth	● Suppresses the NF‐κB pathway	[191]
		● AOM/DSS‐induced CRC mouse model (BALB/c)	●Inhibits tumour growth	● Suppresses the NF‐κB pathway	[193]
Puerarin	*Pueraria lobata* (Willd.) Ohwi.	● AOM/DSS‐induced CRC mouse model (BALB/c)	● Inhibits tumour growth	● Suppresses inflammation	[198]
Cryptotanshinone	*Salvia miltiorrhiza* Bunge	● CT26 cells ● CT26 xenograft mouse model (BALB/c)	● Inhibits tumour growth	● Suppresses inflammation and angiogenesis by regulating MMP/TIMP system, PI3K/AKT/mTOR and HIF‐1α pathway	[202]
Zerumbone	*Zingiber zerumbet* Smith	● SW480 cells	● Inhibits tumour cell growth	● Increases cellular ROS levels and decreases antioxidant levels	[213]
		● HT‐29 cells	● Inhibits tumour cell growth and metastasis	● Increases ROS level	[210]
Dihydroartemisinin	*Artemisia caruifolia* Buch.‐Ham. ex Roxb	● HCT116 and RKO ● HCT116 xenograft mouse model (BALB/c nude mice)	● PromoteS the anti‐tumour activity of oxaliplatin	● Increases PRDX2‐reactive oxygen species‐mediated multiple signalling pathways	[214]
		● HCT116 cells (TP53^−/−^)	● Inhibits tumour cell growth	● Activates ROS‐mediated apoptosis	[215]
Dihydromyricetin	*Vitis heyneana*	● HCT116, HCT8 and HCT116/OXA cells ● NF‐κB/p65‐overexpressed HCT116/OXA cells xenograft mouse model (BALB/c)	● Inhibits tumour growth ● Reverses MRP2‐induced multidrug resistance	● Suppresses NF‐κB‐Nrf2 signalling	[216]
		● COLO205 cells ● COLO205 xenograft mouse model (BALB/c nude mouse)	● Inhibits tumour growth	● Semaphoring 4D is essential for anti‐oxidant and anti‐inflammatory properties	[217]
Andrographolide	*Andrographis paniculata* (Burm.f.) Nees	● SW‐480, DLD‐1, HT‐29 and HCT‐116 cells	● Inhibits tumour cell growth	● Increases intracellular ROS	[218, 219, 220, 221]
		● T84 and COLO205 cells	● Inhibits tumour cell growth	● Increases intracellular ROS	[222]
*Rhus coriaria* extract	*Rhus coriaria*	● HT‐29 and Caco‐2 cells	● Inhibits cell proliferation	● Activates autophagic and apoptotic cell death	[241]
		● HCT‐116‐WT and HCT‐116‐5FU‐R cells	● Inhibits cell proliferation	● Activates autophagic and apoptotic cell death	[243]
*Origanum majorana* ethanolic extract	*Origanum majorana*	● HT‐29 and Caco‐2 cells	● Inhibits cell proliferation	● Activates autophagic and apoptotic cell death	[244]
*Origanum majorana* essential oil	*Origanum majorana*	● HT‐29 cells	● Inhibits cell proliferation	● Activates p38 MAPK‐mediated autophagy, apoptosis and caspase‐dependent cleavage of P70S6K	[245]
Dehydroevodiamine	*Tetradium ruticarpum* (A. Juss.) T. G. Hartley	● HCT116, CT26, SW480 and LoVo cells	● Inhibits cell proliferation ● Inhibits lung metastasis	● Activates caspase‐dependent apoptosis and autophagy ● Regulating epithelial to mesenchymal transition	[246]
Fangchinoline	*Stephaniae tetrandine* S. Moore (Menispermaceae)	● HT29 and HCT116 cells ● HT29 xenograft mouse model (BALB/c nude mice)	● Inhibits tumour cell growth	● Activates AMPK/mTOR/ULK1‐mediated autophagy	[247]

**TABLE 2:** Herbal medicine for CRC treatment.

Number	Herbal medicine	Number of reported
1	Curcumin	176
2	Resveratrol	50
3	Berberine	40
4	Shikonin	20
5	Dihydroartemisinin	18
6	Fucoidan	16
7	Luteolin	14
8	Andrographolide	14
9	Piperine	13
10	Kaempferol	12
11	Emodin	11
12	Cannabidiol	11
13	Tanshinone IIA	11
14	Evodiamine	10
15	Pien Tze Huang	7
16	Baicalein	7
17	Matrine	7
18	Oxymatrine	7
19	Cryptotanshinone	7
20	Oleuropein	7
21	Epigallocatechin‐3‐gallate	6
22	Astragaloside IV	6
23	Ginsenoside Rg3	5
24	Rosmarinic acid	5
25	Zerumbone	5
26	Baicalin	5
27	Quxie capsule	5
28	Phenethyl isothiocyanate	4
29	Wogonin	4
30	Tetrandrine	3
31	Diosgenin	3
32	Lentinan	2

## 6 Discussion on Challenges and Future Perspectives

This review aims to consolidate the current understanding of the anti‐cancer and anti‐metastatic properties, as well as the underlying mechanisms of phytochemicals in CRC. Recent investigations have increasingly concentrated on phytochemicals due to their protective attributes, therapeutic efficacy and favourable safety profiles, positioning them as promising candidates for CRC intervention. Phytochemicals exert their inhibitory effects on CRC tumour growth through a variety of mechanisms, which include immune system modulation (involving CD4^+^/CD8^+^ T lymphocytes, dendritic cells, Th17 cells and regulatory T cells), alteration of gut microbiota, targeting of colorectal cancer stem cells, macrophage polarisation, regulation of glycolysis, induction of ferroptosis, involvement of extracellular vesicles, modulation of mitochondrial function, management of inflammation and oxidative stress and autophagy. Furthermore, several phytochemicals, such as curcumin, resveratrol, berberine, shikonin, dihydroartemisinin, fucoidan, luteolin, andrographolide, piperine, kaempferol, emodin, cannabidiol, tanshinone IIA and evodiamine, have consistently exhibited anti‐CRC effects. Consequently, these phytochemicals may represent promising therapeutic agents or significant lead compounds for the treatment of CRC. Nevertheless, despite the demonstrated effects of these phytochemicals in both in vitro and in vivo studies, further clinical research is essential to substantiate their anti‐cancer and anti‐metastatic efficacy.

## Author Contributions

**Zuqing Su:** writing – original draft, writing – review and editing, visualisation. **Yanlin Li:** writing – review and editing. **Zihao Zhou:** writing – review and editing. **Bing Feng:** supervision, validation, funding acquisition. **Haiming Chen:** supervision, validation, funding acquisition. **Guangjuan Zheng:** supervision, validation, funding acquisition.

## Ethics Statement

This article does not contain any studies with human or animal subjects.

## Conflicts of Interest

The authors declare no conflicts of interest.

## Supporting information


**Table S1.** Herbal medicine for colorectal cancer treatment.

## Data Availability

The data that support the findings of this study are available from the corresponding author upon reasonable request.
