# Supplementary material for: Herbal Medicine for Colorectal Cancer Treatment: Molecular Mechanisms and Clinical Applications
Source: Cell Prolif. 2025 Jun 9;58(10):e70065. doi: 10.1111/cpr.70065 (PMC12508688; doi:10.1111/cpr.70065)
Supplement: Supplementary file 1 — Table S1. Herbal medicine for colorectal cancer treatment. [file CPR-58-e70065-s001.docx]

**Supplementary table 1. Herbal medicine for colorectal cancer treatment**

| Number | Herbal medicine | Number of reported | Reference |
| --- | --- | --- | --- |
| 1 | Curcumin | 176 | [^1-176^](#_ENREF_1) |
| 2 | Resveratrol | 50 | [^55^](#_ENREF_55)^,^[^83^](#_ENREF_83)^,^[^116^](#_ENREF_116)^,^[^177-223^](#_ENREF_177) |
| 3 | Berberine | 40 | [^224-263^](#_ENREF_224) |
| 4 | Shikonin | 20 | [^264-283^](#_ENREF_264) |
| 5 | Dihydroartemisinin | 18 | [^284-301^](#_ENREF_284) |
| 6 | Fucoidan | 16 | [^302-317^](#_ENREF_302) |
| 7 | Luteolin | 14 | [^318-331^](#_ENREF_318) |
| 8 | Andrographolide | 14 | [^332-345^](#_ENREF_332) |
| 9 | Piperine | 13 | [^13^](#_ENREF_13)^,^[^134^](#_ENREF_134)^,^[^346-356^](#_ENREF_346) |
| 10 | Kaempferol | 12 | [^357-368^](#_ENREF_357) |
| 11 | Emodin | 11 | [^369-379^](#_ENREF_369) |
| 12 | Cannabidiol | 11 | [^380-390^](#_ENREF_380) |
| 13 | Tanshinone IIA | 11 | [^391-401^](#_ENREF_391) |
| 14 | Evodiamine | 10 | [^229^](#_ENREF_229)^,^[^402-410^](#_ENREF_402) |
| 15 | Pien Tze Huang | 7 | [^411-417^](#_ENREF_411) |
| 16 | Baicalein | 7 | [^418-424^](#_ENREF_418) |
| 17 | Matrine | 7 | [^425-431^](#_ENREF_425) |
| 18 | Oxymatrine | 7 | [^432-438^](#_ENREF_432) |
| 19 | Cryptotanshinone | 7 | [^439-445^](#_ENREF_439) |
| 20 | Oleuropein | 7 | [^446-452^](#_ENREF_446) |
| 21 | Epigallocatechin-3-Gallate | 6 | [^453-458^](#_ENREF_453) |
| 22 | Astragaloside IV | 6 | [^459-464^](#_ENREF_459) |
| 23 | Ginsenoside Rg3 | 5 | [^465-469^](#_ENREF_465) |
| 24 | Rosmarinic acid | 5 | [^470-474^](#_ENREF_470) |
| 25 | Zerumbone | 5 | [^475-479^](#_ENREF_475) |
| 26 | Baicalin | 5 | [^480-484^](#_ENREF_480) |
| 27 | Quxie capsule | 5 | [^485-489^](#_ENREF_485) |
| 28 | Phenethyl isothiocyanate | 4 | ^[490-493](#_ENREF_490)^ |
| 29 | Wogonin | 4 | [^494-497^](#_ENREF_494) |
| 30 | Tetrandrine | 3 | [^498-500^](#_ENREF_498) |
| 31 | Diosgenin | 3 | [^501-503^](#_ENREF_501) |
| 32 | Lentinan | 2 | [^504^](#_ENREF_504)^,^[^505^](#_ENREF_505) |

**References**

1. Abedi F, Davaran S, Hekmati M, Akbarzadeh A, Baradaran B, Moghaddam SV. An improved method in fabrication of smart dual-responsive nanogels for controlled release of doxorubicin and curcumin in HT-29 colon cancer cells. *J Nanobiotechnology.* 2021;19(1):18.DOI:10.1186/s12951-020-00764-6

2. Ahmed MB, Islam SU, Sonn JK, Lee YS. PRP4 Kinase Domain Loss Nullifies Drug Resistance and Epithelial-Mesenchymal Transition in Human Colorectal Carcinoma Cells. *Molecules & Cells.* 2020;43(7):662-670.DOI:10.14348/molcells.2020.2263

3. Alam MN, Moni MA, Yu JQ, et al. Promising Anticancer Activity of [Bis(1,8-quinolato)palladium (II)] Alone and in Combination. *Int J Mol Sci.* 2021;22(16).DOI:10.3390/ijms22168471

4. Alizadeh MH, Pooresmaeil M, Namazi H. Carboxymethyl cellulose@multi wall carbon nanotubes functionalized with Ugi reaction as a new curcumin carrier. *Int J Biol Macromol.* 2023;234:123778.DOI:10.1016/j.ijbiomac.2023.123778

5. Almoshari Y, Iqbal H, Razzaq A, et al. Development of nanocubosomes co-loaded with dual anticancer agents curcumin and temozolomide for effective Colon cancer therapy. *Drug Deliv.* 2022;29(1):2633-2643.DOI:10.1080/10717544.2022.2108938

6. An SY, Kim KS, Lee YC, Kim SH. Transcription of human β-galactoside α2,6-sialyltransferase (hST6Gal I) is downregulated by curcumin through AMPK signaling in human colon carcinoma HCT116 cells. *Genes Genomics.* 2023;45(7):901-909.DOI:10.1007/s13258-023-01398-2

7. Atwan QS, Al-Ogaidi I. Enhancing the therapeutic potential of curcumin: a novel nanoformulation for targeted anticancer therapy to colorectal cancer with reduced miR20a and miR21 expression. *Biomed Mater.* 2024;19(2).DOI:10.1088/1748-605X/ad1dfc

8. Azeez HJ, Neri F, Hosseinpour Feizi MA, Babaei E. Transcriptome Profiling of HCT-116 Colorectal Cancer Cells with RNA Sequencing Reveals Novel Targets for Polyphenol Nano Curcumin. *Molecules.* 2022;27(11).DOI:10.3390/molecules27113470

9. Babaei M, Abrishami A, Iranpour S, Saljooghi AS, Matin MM. Harnessing curcumin in a multifunctional biodegradable metal-organic framework (bio-MOF) for targeted colorectal cancer theranostics. *Drug Deliv Transl Res.* 2024.DOI:10.1007/s13346-024-01707-6

10. Bahman A, Abaza MS, Khoushaish S, Al-Attiyah RJ. Therapeutic efficacy of sorafenib and plant-derived phytochemicals in human colorectal cancer cells. *BMC Complement Med Ther.* 2023;23(1):210.DOI:10.1186/s12906-023-04032-6

11. Baptistella MM, Assunção RRS, Sales de Oliveira C, et al. A synthetic resveratrol-curcumin hybrid derivative exhibits chemopreventive effects on colon pre-neoplastic lesions by targeting Wnt/β-catenin signaling, anti-inflammatory and antioxidant pathways. *Journal of Pharmacy & Pharmacology.* 2024;76(5):479-488.DOI:10.1093/jpp/rgad077

12. Bardania H, Jafari F, Baneshi M, et al. Folic Acid-Functionalized Albumin/Graphene Oxide Nanocomposite to Simultaneously Deliver Curcumin and 5-Fluorouracil into Human Colorectal Cancer Cells: An In Vitro Study. *Biomed Res Int.* 2023;2023:8334102.DOI:10.1155/2023/8334102

13. Bolat ZB, Islek Z, Demir BN, Yilmaz EN, Sahin F, Ucisik MH. Curcumin- and Piperine-Loaded Emulsomes as Combinational Treatment Approach Enhance the Anticancer Activity of Curcumin on HCT116 Colorectal Cancer Model. *Front Bioeng Biotechnol.* 2020;8:50.DOI:10.3389/fbioe.2020.00050

14. Borah PK, Das AS, Mukhopadhyay R, Sarkar A, Duary RK. Macromolecular design of folic acid functionalized amylopectin-albumin core-shell nanogels for improved physiological stability and colon cancer cell targeted delivery of curcumin. *Journal of Colloid & Interface Science.* 2020;580:561-572.DOI:10.1016/j.jcis.2020.07.056

15. Briata IM, Paleari L, Rutigliani M, et al. A Presurgical Study of Curcumin Combined with Anthocyanin Supplements in Patients with Colorectal Adenomatous Polyps. *Int J Mol Sci.* 2021;22(20).DOI:10.3390/ijms222011024

16. Cerqueira R, Domingues C, Veiga F, Jarak I, Figueiras A. Development and Characterization of Curcumin-Loaded TPGS/F127/P123 Polymeric Micelles as a Potential Therapy for Colorectal Cancer. *Int J Mol Sci.* 2024;25(14).DOI:10.3390/ijms25147577

17. Chen J, Xue F, Du W, et al. An Endogenous H(2)S-Activated Nanoplatform for Triple Synergistic Therapy of Colorectal Cancer. *Nano Lett.* 2022;22(15):6156-6165.DOI:10.1021/acs.nanolett.2c01346

18. Chen L, Dai Z, Ge C, et al. Specific metabolic response of patient-derived organoids to curcumin of colorectal cancer. *Journal of Chromatography B: Analytical Technologies in the Biom.* 2022;1203:123260.DOI:10.1016/j.jchromb.2022.123260

19. Chen M, Tan AH, Li J. Curcumin Represses Colorectal Cancer Cell Proliferation by Triggering Ferroptosis via PI3K/Akt/mTOR Signaling. *Nutrition & Cancer.* 2023;75(2):726-733.DOI:10.1080/01635581.2022.2139398

20. Chen S, Gao W, Ge P, et al. Negatively Charged Thermosensitive Hydrogel Loaded with Pectin Microspheres to Recover the Mucosal Barrier for Ulcerative Colitis Therapy. *Biomacromolecules.* 2024;25(10):6801-6813.DOI:10.1021/acs.biomac.4c01019

21. Chen T, Yang C, Xi Z, Chen F, Li H. Reduced Caudal Type Homeobox 2 (CDX2) Promoter Methylation Is Associated with Curcumin's Suppressive Effects on Epithelial-Mesenchymal Transition in Colorectal Cancer Cells. *Med Sci Monit.* 2020;26:e926443.DOI:10.12659/msm.926443

22. Chou YT, Koh YC, Nagabhushanam K, Ho CT, Pan MH. A Natural Degradant of Curcumin, Feruloylacetone Inhibits Cell Proliferation via Inducing Cell Cycle Arrest and a Mitochondrial Apoptotic Pathway in HCT116 Colon Cancer Cells. *Molecules.* 2021;26(16).DOI:10.3390/molecules26164884

23. Dal Z, Aru B. The role of curcumin on apoptosis and NLRP3 inflammasome-dependent pyroptosis on colorectal cancer in vitro. *Turk J Med Sci.* 2023;53(4):883-893.DOI:10.55730/1300-0144.5652

24. Das T, Dvoretskiy S, Chen C, Luo M, Pereira SL. Fish Oil, Plant Polyphenols, and Their Combinations Have No Tumor Growth Promoting Effects on Human Lung and Colon Carcinoma Xenograft Mice. *J Diet Suppl.* 2023;20(3):459-474.DOI:10.1080/19390211.2021.2021344

25. de Freitas CF, Kimura E, Rubira AF, Muniz EC. Curcumin and silver nanoparticles carried out from polysaccharide-based hydrogels improved the photodynamic properties of curcumin through metal-enhanced singlet oxygen effect. *Mater Sci Eng C Mater Biol Appl.* 2020;112:110853.DOI:10.1016/j.msec.2020.110853

26. Deng W, Xiong X, Lu M, et al. Curcumin suppresses colorectal tumorigenesis through restoring the gut microbiota and metabolites. *BMC Cancer.* 2024;24(1):1141.DOI:10.1186/s12885-024-12898-z

27. Dent P, Booth L, Roberts JL, Poklepovic A, Hancock JF. (Curcumin+sildenafil) enhances the efficacy of 5FU and anti-PD1 therapies in vivo. *J Cell Physiol.* 2020;235(10):6862-6874.DOI:10.1002/jcp.29580

28. DiMarco-Crook C, Rakariyatham K, Li Z, et al. Synergistic anticancer effects of curcumin and 3',4'-didemethylnobiletin in combination on colon cancer cells. *J Food Sci.* 2020;85(4):1292-1301.DOI:10.1111/1750-3841.15073

29. Ebrahimi M, Babaei E, Neri F, Feizi MAH. Anti-proliferative and apoptotic effect of gemini curcumin in p53-wild type and p53-mutant colorectal cancer cell lines. *Int J Pharm.* 2021;601:120592.DOI:10.1016/j.ijpharm.2021.120592

30. Ejaz SA, Aziz M, Fawzy Ramadan M, Fayyaz A, Bilal MS. Pharmacophore-Based Virtual Screening and In-Silico Explorations of Biomolecules (Curcumin Derivatives) of Curcuma longa as Potential Lead Inhibitors of ERBB and VEGFR-2 for the Treatment of Colorectal Cancer. *Molecules.* 2023;28(10).DOI:10.3390/molecules28104044

31. Elbadawy M, Hayashi K, Ayame H, et al. Anti-cancer activity of amorphous curcumin preparation in patient-derived colorectal cancer organoids. *Biomedicine & Pharmacotherapy.* 2021;142:112043.DOI:10.1016/j.biopha.2021.112043

32. Elbassiouni FE, El-Kholy WM, Elhabibi EM, Albogami S, Fayad E. Comparative Study between Curcumin and Nanocurcumin Loaded PLGA on Colon Carcinogenesis Induced Mice. *Nanomaterials (Basel).* 2022;12(3).DOI:10.3390/nano12030324

33. El-Far AH, Darwish NHE, Mousa SA. Senescent Colon and Breast Cancer Cells Induced by Doxorubicin Exhibit Enhanced Sensitivity to Curcumin, Caffeine, and Thymoquinone. *Integr Cancer Ther.* 2020;19:1534735419901160.DOI:10.1177/1534735419901160

34. El-Sherbiny MM, Elekhtiar RS, El-Hefnawy ME, et al. Fabrication and assessment of potent anticancer nanoconjugates from chitosan nanoparticles, curcumin, and eugenol. *Front Bioeng Biotechnol.* 2022;10:1030936.DOI:10.3389/fbioe.2022.1030936

35. Fan WH, Wang FC, Jin Z, Zhu L, Zhang JX. Curcumin Synergizes with Cisplatin to Inhibit Colon Cancer through Targeting the MicroRNA-137-Glutaminase Axis. *Curr Med Sci.* 2022;42(1):108-117.DOI:10.1007/s11596-021-2469-0

36. Fan X, Zhu M, Qiu F, et al. Curcumin may be a potential adjuvant treatment drug for colon cancer by targeting CD44. *Int Immunopharmacol.* 2020;88:106991.DOI:10.1016/j.intimp.2020.106991

37. Farhana L, Sarkar S, Nangia-Makker P, et al. Natural agents inhibit colon cancer cell proliferation and alter microbial diversity in mice. *PLoS One.* 2020;15(3):e0229823.DOI:10.1371/journal.pone.0229823

38. Femia AP, Soares PV, Luceri C, Lodovici M, Giannini A, Caderni G. Sulindac, 3,3'-diindolylmethane and curcumin reduce carcinogenesis in the Pirc rat, an Apc-driven model of colon carcinogenesis. *BMC Cancer.* 2015;15:611.DOI:10.1186/s12885-015-1627-9

39. Fenton JI, McCaskey SJ. Curcumin and docosahexaenoic acid block insulin-induced colon carcinoma cell proliferation. *Prostaglandins, Leukotrienes, & Essential Fatty Acids.* 2013;88(3):219-226.DOI:10.1016/j.plefa.2012.11.010

40. Firouzi Amandi A, Jokar E, Eslami M, et al. Enhanced anti-cancer effect of artemisinin- and curcumin-loaded niosomal nanoparticles against human colon cancer cells. *Med Oncol.* 2023;40(6):170.DOI:10.1007/s12032-023-02032-7

41. Firouzjaei AA, Aghaee-Bakhtiari SH, Tafti A, et al. Impact of curcumin on ferroptosis-related genes in colorectal cancer: Insights from in-silico and in-vitro studies. *Cell Biochemistry & Function.* 2023;41(8):1488-1502.DOI:10.1002/cbf.3889

42. Fulgheri F, Aroffu M, Ramírez M, et al. Curcumin or quercetin loaded nutriosomes as oral adjuvants for malaria infections. *Int J Pharm.* 2023;643:123195.DOI:10.1016/j.ijpharm.2023.123195

43. Gavrilas LI, Cruceriu D, Ionescu C, Miere D, Balacescu O. Pro-apoptotic genes as new targets for single and combinatorial treatments with resveratrol and curcumin in colorectal cancer. *Food Funct.* 2019;10(6):3717-3726.DOI:10.1039/c9fo01014a

44. Ge S, Sun X, Sang L, et al. Curcumin inhibits malignant behavior of colorectal cancer cells by regulating M2 polarization of tumor-associated macrophages and metastasis associated in colon cancer 1 (MACC1) expression. *Chem Biol Drug Des.* 2023;102(5):1202-1212.DOI:10.1111/cbdd.14330

45. Gholipour F, Amini M, Baradaran B, Mokhtarzadeh A, Eskandani M. Anticancer properties of curcumin-treated Lactobacillus plantarum against the HT-29 colorectal adenocarcinoma cells. *Sci Rep.* 2023;13(1):2860.DOI:10.1038/s41598-023-29462-7

46. Ghorbani Z, Heidari M, Jafarinia M, Rohani M, Akbari A. Transcriptional Regulation of the Colorectal Cancer Stem Cell Markers, Nanog and Oct4, Induced by a Thermodynamic-Based Therapy Approach. *Iran J Public Health.* 2023;52(4):848-856.DOI:10.18502/ijph.v52i4.12458

47. Gong F, Ma JC, Jia J, et al. Synergistic effect of the anti-PD-1 antibody with blood stable and reduction sensitive curcumin micelles on colon cancer. *Drug Deliv.* 2021;28(1):930-942.DOI:10.1080/10717544.2021.1921077

48. Guéguinou M, Ibrahim S, Bourgeais J, et al. Curcumin and NCLX inhibitors share anti-tumoral mechanisms in microsatellite-instability-driven colorectal cancer. *Cellular & Molecular Life Sciences.* 2022;79(6):284.DOI:10.1007/s00018-022-04311-4

49. Güllü N, Smith J, Herrmann P, Stein U. MACC1-Dependent Antitumor Effect of Curcumin in Colorectal Cancer. *Nutrients.* 2022;14(22).DOI:10.3390/nu14224792

50. Gunther JR, Chadha AS, Guha S, et al. A phase II randomized double blinded trial evaluating the efficacy of curcumin with pre-operative chemoradiation for rectal cancer. *J Gastrointest Oncol.* 2022;13(6):2938-2950.DOI:10.21037/jgo-22-259

51. Han W, Yin H, Ma H, Wang Y, Kong D, Fan Z. Curcumin Regulates ERCC1 Expression and Enhances Oxaliplatin Sensitivity in Resistant Colorectal Cancer Cells through Its Effects on miR-409-3p. *Evidence-Based Complementary & Alternative Medicine.* 2020;2020:8394574.DOI:10.1155/2020/8394574

52. Han X, Yang C, Guo C, et al. Bioinformatics Analysis to Screen Key Targets of Curcumin against Colorectal Cancer and the Correlation with Tumor-Infiltrating Immune Cells. *Evidence-Based Complementary & Alternative Medicine.* 2021;2021:9132608.DOI:10.1155/2021/9132608

53. Han Z, Song B, Yang J, et al. Curcumin-Encapsulated Fusion Protein-Based Nanocarrier Demonstrated Highly Efficient Epidermal Growth Factor Receptor-Targeted Treatment of Colorectal Cancer. *Journal of Agricultural & Food Chemistry.* 2022;70(49):15464-15473.DOI:10.1021/acs.jafc.2c04668

54. Hao J, Dai X, Gao J, et al. Curcumin suppresses colorectal tumorigenesis via the Wnt/β-catenin signaling pathway by downregulating Axin2. *Oncol Lett.* 2021;21(3):186.DOI:10.3892/ol.2021.12447

55. Hernández C, Moreno G, Herrera RA, Cardona GW. New Hybrids Based on Curcumin and Resveratrol: Synthesis, Cytotoxicity and Antiproliferative Activity against Colorectal Cancer Cells. *Molecules.* 2021;26(9).DOI:10.3390/molecules26092661

56. Herrero de la Parte B, Rodeño-Casado M, Iturrizaga Correcher S, Mar Medina C, García-Alonso I. Curcumin Reduces Colorectal Cancer Cell Proliferation and Migration and Slows In Vivo Growth of Liver Metastases in Rats. *Biomedicines.* 2021;9(9).DOI:10.3390/biomedicines9091183

57. Hon KW, Zainal Abidin SA, Abas F, Othman I, Naidu R. Anti-Cancer Mechanisms of Diarylpentanoid MS17 (1,5-Bis(2-hydroxyphenyl)-1,4-pentadiene-3-one) in Human Colon Cancer Cells: A Proteomics Approach. *Int J Mol Sci.* 2024;25(6).DOI:10.3390/ijms25063503

58. Hosokawa M, Seiki R, Iwakawa S, Ogawara KI. Combination of azacytidine and curcumin is a potential alternative in decitabine-resistant colorectal cancer cells with attenuated deoxycytidine kinase. *Biochemical & Biophysical Research Communications.* 2021;578:157-162.DOI:10.1016/j.bbrc.2021.09.041

59. Hosseini SS, Reihani RZ, Doustvandi MA, et al. Synergistic anticancer effects of curcumin and crocin on human colorectal cancer cells. *Mol Biol Rep.* 2022;49(9):8741-8752.DOI:10.1007/s11033-022-07719-0

60. Hou C, Hu Y, Zhang T. Research on curcumin mediating immunotherapy of colorectal cancer by regulating cancer associated fibroblasts. *Anticancer Drugs.* 2024.DOI:10.1097/cad.0000000000001659

61. Hu D, Meng RY, Nguyen TV, et al. Inhibition of colorectal cancer tumorigenesis by ursolic acid and doxorubicin is mediated by targeting the Akt signaling pathway and activating the Hippo signaling pathway. *Mol Med Rep.* 2023;27(1).DOI:10.3892/mmr.2022.12898

62. Hu S, Xia K, Huang X, et al. Multifunctional CaCO(3)@Cur@QTX125@HA nanoparticles for effectively inhibiting growth of colorectal cancer cells. *J Nanobiotechnology.* 2023;21(1):353.DOI:10.1186/s12951-023-02104-w

63. Hu Y, He Y, Ji J, Zheng S, Cheng Y. Tumor Targeted Curcumin Delivery by Folate-Modified MPEG-PCL Self-Assembly Micelles for Colorectal Cancer Therapy. *Int J Nanomedicine.* 2020;15:1239-1252.DOI:10.2147/ijn.s232777

64. Idoudi S, Bedhiafi T, Sahir F, et al. Studies on anti-colon cancer potential of nanoformulations of curcumin and succinylated curcumin in mannosylated chitosan. *Int J Biol Macromol.* 2023;235:123827.DOI:10.1016/j.ijbiomac.2023.123827

65. Inphonlek S, Sunintaboon P, Léonard M, Durand A. Chitosan/carboxymethylcellulose-stabilized poly(lactide-co-glycolide) particles as bio-based drug delivery carriers. *Carbohydr Polym.* 2020;242:116417.DOI:10.1016/j.carbpol.2020.116417

66. Izadi Z, Rashidi M, Derakhshankhah H, et al. Curcumin-loaded porous particles functionalized with pH-responsive cell-penetrating peptide for colorectal cancer targeted drug delivery. *RSC Adv.* 2023;13(49):34587-34597.DOI:10.1039/d3ra06270h

67. Jain S, Lenaghan S, Dia V, Zhong Q. Co-delivery of curcumin and quercetin in shellac nanocapsules for the synergistic antioxidant properties and cytotoxicity against colon cancer cells. *Food Chem.* 2023;428:136744.DOI:10.1016/j.foodchem.2023.136744

68. Jamal Moideen MM, Alqahtani A, Venkatesan K, et al. Application of the Box-Behnken design for the production of soluble curcumin: Skimmed milk powder inclusion complex for improving the treatment of colorectal cancer. *Food Sci Nutr.* 2020;8(12):6643-6659.DOI:10.1002/fsn3.1957

69. Jamialahmadi T, Guest PC, Afshari AR, Majeed M, Sahebkar A. Testing the Effect of Curcumin on Proliferative Capacity of Colorectal Cancer Cells. *Methods Mol Biol.* 2022;2343:287-298.DOI:10.1007/978-1-0716-1558-4_20

70. Jayarajan J, Angandoor S, Vedulla SH, et al. Curcumin induces chemosensitization to doxorubicin in Duke's type B coloadenocarcinoma cell line. *Mol Biol Rep.* 2020;47(10):7883-7892.DOI:10.1007/s11033-020-05866-w

71. Jeon Y, Sym SJ, Yoo BK, Baek JH. Long-term Survival, Tolerability, and Safety of First-Line Bevacizumab and FOLFIRI in Combination With Ginsenoside-Modified Nanostructured Lipid Carrier Containing Curcumin in Patients With Unresectable Metastatic Colorectal Cancer. *Integr Cancer Ther.* 2022;21:15347354221105498.DOI:10.1177/15347354221105498

72. Jia F, Li Y, Deng X, et al. Self-assembled fluorescent hybrid nanoparticles-mediated collaborative lncRNA CCAT1 silencing and curcumin delivery for synchronous colorectal cancer theranostics. *J Nanobiotechnology.* 2021;19(1):238.DOI:10.1186/s12951-021-00981-7

73. Kabagwira J, Fuller RN, Vallejos PA, et al. Amplifying Curcumin's Antitumor Potential: A Heat-Driven Approach for Colorectal Cancer Treatment. *OncoTargets & Therapy.* 2024;17:63-78.DOI:10.2147/ott.s448024

74. Kane AM, Liu C, Akhter DT, et al. Curcumin Chemoprevention Reduces the Incidence of Braf Mutant Colorectal Cancer in a Preclinical Study. *Digestive Diseases & Sciences.* 2021;66(12):4326-4332.DOI:10.1007/s10620-020-06752-y

75. Karthika C, Rahman MH, Sureshkumar R, et al. 5-Fluorouracil and Curcumin Combination Coated with Pectin and Its Strategy towards Titanium Dioxide, Dimethylhydrazine Colorectal Cancer Model with the Evaluation of the Blood Parameters. *Polymers (Basel).* 2022;14(14).DOI:10.3390/polym14142868

76. Karthika C, Sureshkumar R, Sajini DV, Ashraf GM, Rahman MH. 5-fluorouracil and curcumin with pectin coating as a treatment regimen for titanium dioxide with dimethylhydrazine-induced colon cancer model. *Environmental Science & Pollution Research International.* 2022;29(42):63202-63215.DOI:10.1007/s11356-022-20208-y

77. Khaket TP, Singh MP, Khan I, Kang SC. In vitro and in vivo studies on potentiation of curcumin-induced lysosomal-dependent apoptosis upon silencing of cathepsin C in colorectal cancer cells. *Pharmacol Res.* 2020;161:105156.DOI:10.1016/j.phrs.2020.105156

78. Khan FA, Lammari N, Muhammad Siar AS, et al. Quantum dots encapsulated with curcumin inhibit the growth of colon cancer, breast cancer and bacterial cells. *Nanomedicine (Lond).* 2020;15(10):969-980.DOI:10.2217/nnm-2019-0429

79. Khan S, Miles GJ, Demetriou C, et al. Ex vivo explant model of adenoma and colorectal cancer to explore mechanisms of action and patient response to cancer prevention therapies. *Mutagenesis.* 2022;37(5-6):227-237.DOI:10.1093/mutage/geac020

80. Kucukkaraduman B, Cicek EG, Akbar MW, Demirkol Canli S, Vural B, Gure AO. Epithelial-to-Mesenchymal Transition Is Not a Major Modulating Factor in the Cytotoxic Response to Natural Products in Cancer Cell Lines. *Molecules.* 2021;26(19).DOI:10.3390/molecules26195858

81. Kulbacka J, Wilk KA, Bazylińska U, Dubińska-Magiera M, Potoczek S, Saczko J. Curcumin Loaded Nanocarriers with Varying Charges Augmented with Electroporation Designed for Colon Cancer Therapy. *Int J Mol Sci.* 2022;23(3).DOI:10.3390/ijms23031377

82. Kumar A, Singam A, Swaminathan G, et al. Combinatorial therapy using RNAi and curcumin nano-architectures regresses tumors in breast and colon cancer models. *Nanoscale.* 2022;14(2):492-505.DOI:10.1039/d1nr04411g

83. Kuo IM, Lee JJ, Wang YS, et al. Potential enhancement of host immunity and anti-tumor efficacy of nanoscale curcumin and resveratrol in colorectal cancers by modulated electro- hyperthermia. *BMC Cancer.* 2020;20(1):603.DOI:10.1186/s12885-020-07072-0

84. Kwaśnik P, Lemieszek MK, Rzeski W. Impact of phytochemicals and plant extracts on viability and proliferation of NK cell line NK-92 - a closer look at immunomodulatory properties of goji berries extract in human colon cancer cells. *Annals of Agricultural & Environmental Medicine.* 2021;28(2):291-299.DOI:10.26444/aaem/133801

85. La Rocca A, De Gregorio V, Lagreca E, Vecchione R, Netti PA, Imparato G. Colorectal Cancer Bioengineered Microtissues as a Model to Replicate Tumor-ECM Crosstalk and Assess Drug Delivery Systems In Vitro. *Int J Mol Sci.* 2023;24(6).DOI:10.3390/ijms24065678

86. Laali KK, Zwarycz AT, Beck N, Borosky GL, Nukaya M, Kennedy GD. Curcumin Conjugates of Non-steroidal Anti-Inflammatory Drugs: Synthesis, Structures, Anti-proliferative Assays, Computational Docking, and Inflammatory Response. *ChemistryOpen.* 2020;9(8):822-834.DOI:10.1002/open.202000173

87. Laka K, Mapheto KBF, Mbita Z. Selective in vitro cytotoxicity effect of Drimia calcarata bulb extracts against p53 mutant HT-29 and p53 wild-type Caco-2 colorectal cancer cells through STAT5B regulation. *Toxicol Rep.* 2021;8:1265-1279.DOI:10.1016/j.toxrep.2021.06.015

88. Lambring C, Varga K, Livingston K, Lorusso N, Dudhia A, Basha R. Therapeutic Applications of Curcumin and Derivatives in Colorectal Cancer. *Onco Ther.* 2022;9(1):51-62.DOI:10.1615/OncoTherap.2022044575

89. Li G, Fang S, Shao X, et al. Curcumin Reverses NNMT-Induced 5-Fluorouracil Resistance via Increasing ROS and Cell Cycle Arrest in Colorectal Cancer Cells. *Biomolecules.* 2021;11(9).DOI:10.3390/biom11091295

90. Li M, Liu Y, Liu Y, et al. Fabrication of targeted and pH responsive lysozyme-hyaluronan nanoparticles for 5-fluorouracil and curcumin co-delivery in colorectal cancer therapy. *Int J Biol Macromol.* 2024;254(Pt 2):127836.DOI:10.1016/j.ijbiomac.2023.127836

91. Li S, Fu H, Wang Y, Wang L, Jia B, Bian Y. Curcumin inhibits CT26 cells metastasis by decreasing heparanase expression. *J Leukoc Biol.* 2020;108(6):1727-1733.DOI:10.1002/jlb.1ma0620-357r

92. Li X, He Y, Zhang S, et al. Lactoferrin-Based Ternary Composite Nanoparticles with Enhanced Dispersibility and Stability for Curcumin Delivery. *ACS Appl Mater Interfaces.* 2023;15(14):18166-18181.DOI:10.1021/acsami.2c20816

93. Li Y, Su Y, Pan H, et al. Nanodiamond-based multifunctional platform for oral chemo-photothermal combinational therapy of orthotopic colon cancer. *Pharmacol Res.* 2022;176:106080.DOI:10.1016/j.phrs.2022.106080

94. Liu C, Rokavec M, Huang Z, Hermeking H. Curcumin activates a ROS/KEAP1/NRF2/miR-34a/b/c cascade to suppress colorectal cancer metastasis. *Cell Death & Differentiation.* 2023;30(7):1771-1785.DOI:10.1038/s41418-023-01178-1

95. Liu F, Zhu C, Ma H, Yang Q. Curcumin targets miR-134-5p to suppress the progression of colorectal cancer through regulating the CDCA3/CDK1 pathway. *Naunyn Schmiedebergs Arch Pharmacol.* 2024;397(1):109-122.DOI:10.1007/s00210-023-02584-5

96. Liu G, Chen J, Bao Z. Promising antitumor effects of the curcumin analog DMC-BH on colorectal cancer cells. *Aging (Albany NY).* 2023;15(6):2221-2236.DOI:10.18632/aging.204610

97. Liu J, Li L, Zhang B, Xu ZP. MnO(2)-shelled Doxorubicin/Curcumin nanoformulation for enhanced colorectal cancer chemo-immunotherapy. *Journal of Colloid & Interface Science.* 2022;617:315-325.DOI:10.1016/j.jcis.2022.02.132

98. Liu L, Yang S, Chen F, Cheng KW. Polysaccharide-Zein Composite Nanoparticles for Enhancing Cellular Uptake and Oral Bioavailability of Curcumin: Characterization, Anti-colorectal Cancer Effect, and Pharmacokinetics. *Front Nutr.* 2022;9:846282.DOI:10.3389/fnut.2022.846282

99. Liu L, Yang S, Chen F, Cheng KW. Hyaluronic Acid-Zein Core-Shell Nanoparticles Improve the Anticancer Effect of Curcumin Alone or in Combination with Oxaliplatin against Colorectal Cancer via CD44-Mediated Cellular Uptake. *Molecules.* 2022;27(5).DOI:10.3390/molecules27051498

100. Low ZX, Teo MYM, Juliana Nordin F, et al. Enhancing the solubility and potency of tetrahydrocurcumin as an anti-cancer agent using a β-cyclodextrin inclusion complex approach. *PLoS One.* 2024;19(7):e0305171.DOI:10.1371/journal.pone.0305171

101. Low ZX, Teo MYM, Nordin FJ, Dewi FRP, Palanirajan VK, In LLA. Biophysical Evaluation of Water-Soluble Curcumin Encapsulated in β-Cyclodextrins on Colorectal Cancer Cells. *Int J Mol Sci.* 2022;23(21).DOI:10.3390/ijms232112866

102. Lu L, Przybylla R, Shang Y, et al. Microsatellite Status and IκBα Expression Levels Predict Sensitivity to Pharmaceutical Curcumin in Colorectal Cancer Cells. *Cancers (Basel).* 2022;14(4).DOI:10.3390/cancers14041032

103. Lu Y, Zhang R, Zhang X, Zhang B, Yao Q. Curcumin may reverse 5-fluorouracil resistance on colonic cancer cells by regulating TET1-NKD-Wnt signal pathway to inhibit the EMT progress. *Biomedicine & Pharmacotherapy.* 2020;129:110381.DOI:10.1016/j.biopha.2020.110381

104. Ma Y, Thurecht KJ, Coombes AGA. Development of enteric-coated, biphasic chitosan/HPMC microcapsules for colon-targeted delivery of anticancer drug-loaded nanoparticles. *Int J Pharm.* 2021;607:121026.DOI:10.1016/j.ijpharm.2021.121026

105. Macis D, Briata IM, D'Ecclesiis O, et al. Inflammatory and Metabolic Biomarker Assessment in a Randomized Presurgical Trial of Curcumin and Anthocyanin Supplements in Patients with Colorectal Adenomas. *Nutrients.* 2023;15(18).DOI:10.3390/nu15183894

106. Mahmoudi A, Jamialahmadi T, Kesharwani P, Sahebkar A. Bioinformatic analysis of the molecular targets of curcumin in colorectal cancer. *Pathology, Research & Practice.* 2024;262:155533.DOI:10.1016/j.prp.2024.155533

107. Mao X, Zhang X, Zheng X, Chen Y, Xuan Z, Huang P. Curcumin suppresses LGR5(+) colorectal cancer stem cells by inducing autophagy and via repressing TFAP2A-mediated ECM pathway. *J Nat Med.* 2021;75(3):590-601.DOI:10.1007/s11418-021-01505-1

108. Mari M, Boniburini M, Tosato M, et al. Development of Stable Amino-Pyrimidine-Curcumin Analogs: Synthesis, Equilibria in Solution, and Potential Anti-Proliferative Activity. *Int J Mol Sci.* 2023;24(18).DOI:10.3390/ijms241813963

109. Mari M, Boniburini M, Tosato M, et al. Bridging pyrimidine hemicurcumin and Cisplatin: Synthesis, coordination chemistry, and in vitro activity assessment of a novel Pt(II) complex. *J Inorg Biochem.* 2024;260:112702.DOI:10.1016/j.jinorgbio.2024.112702

110. Min Z, Zhu Y, Hong X, et al. Synthesis and Biological Evaluations of Monocarbonyl Curcumin Inspired Pyrazole Analogues as Potential Anti-Colon Cancer Agent. *Drug Design, Development & Therapy.* 2020;14:2517-2534.DOI:10.2147/dddt.s244865

111. Ming T, Lei J, Peng Y, et al. Curcumin suppresses colorectal cancer by induction of ferroptosis via regulation of p53 and solute carrier family 7 member 11/glutathione/glutathione peroxidase 4 signaling axis. *Phytother Res.* 2024;38(8):3954-3972.DOI:10.1002/ptr.8258

112. Miyazaki K, Xu C, Shimada M, Goel A. Curcumin and Andrographis Exhibit Anti-Tumor Effects in Colorectal Cancer via Activation of Ferroptosis and Dual Suppression of Glutathione Peroxidase-4 and Ferroptosis Suppressor Protein-1. *Pharmaceuticals (Basel).* 2023;16(3).DOI:10.3390/ph16030383

113. Mohamed JM, Alqahtani A, Ahmad F, Krishnaraju V, Kalpana K. Pectin co-functionalized dual layered solid lipid nanoparticle made by soluble curcumin for the targeted potential treatment of colorectal cancer. *Carbohydr Polym.* 2021;252:117180.DOI:10.1016/j.carbpol.2020.117180

114. Mohamed JMM, Alqahtani A, Ahmad F, Krishnaraju V, Kalpana K. Stoichiometrically Governed Curcumin Solid Dispersion and Its Cytotoxic Evaluation on Colorectal Adenocarcinoma Cells. *Drug Design, Development & Therapy.* 2020;14:4639-4658.DOI:10.2147/dddt.s273322

115. Mohamed JMM, Alqahtani A, Khan BA, et al. Preparation of Soluble Complex of Curcumin for the Potential Antagonistic Effects on Human Colorectal Adenocarcinoma Cells. *Pharmaceuticals (Basel).* 2021;14(9).DOI:10.3390/ph14090939

116. Moreno QG, Herrera RA, Yepes AF, Naranjo TW, Cardona GW. Proapoptotic Effect and Molecular Docking Analysis of Curcumin-Resveratrol Hybrids in Colorectal Cancer Chemoprevention. *Molecules.* 2022;27(11).DOI:10.3390/molecules27113486

117. Moreno-Marín JP, Estrada V, Castro C, et al. Encapsulation of a 5FU-curcumin hybrid on bacterial nanocellulose for colorectal cancer treatment. *Int J Biol Macromol.* 2024;281(Pt 4):136650.DOI:10.1016/j.ijbiomac.2024.136650

118. Moreno-Quintero G, Betancur-Zapata E, Herrera-Ramírez A, Cardona-Galeano W. New Hybrid Scaffolds Based on 5-FU/Curcumin: Synthesis, Cytotoxic, Antiproliferative and Pro-Apoptotic Effect. *Pharmaceutics.* 2023;15(4).DOI:10.3390/pharmaceutics15041221

119. Mortezapour M, Tapak L, Bahreini F, Najafi R, Afshar S. Identification of key genes in colorectal cancer diagnosis by co-expression analysis weighted gene co-expression network analysis. *Computers in Biology & Medicine.* 2023;157:106779.DOI:10.1016/j.compbiomed.2023.106779

120. Mousazadeh N, Gharbavi M, Rashidzadeh H, Nosrati H, Danafar H, Johari B. Anticancer evaluation of methotrexate and curcumin-coencapsulated niosomes against colorectal cancer cell lines. *Nanomedicine (Lond).* 2022;17(4):201-217.DOI:10.2217/nnm-2021-0334

121. Muthu Mohamed JM, Kavitha K, Ahmad F, et al. Curcumin Plant for Colorectal Cancer Prediction and Prevention Using In Silico Molecular Analysis; HOT-MELT Extrusion. *Evidence-Based Complementary & Alternative Medicine.* 2022;2022:4376960.DOI:10.1155/2022/4376960

122. Omar AM, El-Araby ME, Abdelghany TM, et al. Introducing of potent cytotoxic novel 2-(aroylamino)cinnamamide derivatives against colon cancer mediated by dual apoptotic signal activation and oxidative stress. *Bioorg Chem.* 2020;101:103953.DOI:10.1016/j.bioorg.2020.103953

123. Pooresmaeil M, Namazi H. Development of the new pH-driven carrier from alginate/carboxymethyl starch bio-coated co-drugs@COF-OH for controlled and concomitant colon cancer treatment. *Int J Biol Macromol.* 2023;239:124322.DOI:10.1016/j.ijbiomac.2023.124322

124. Qin S, Su Q, Li X, et al. Curcumin suppresses cell proliferation and reduces cholesterol absorption in Caco-2 cells by activating the TRPA1 channel. *Lipids in Health & Disease.* 2023;22(1):6.DOI:10.1186/s12944-022-01750-7

125. Rezaeian A, Amini SM, Najafabadi MRH, Farsangi ZJ, Samadian H. Plasmonic hyperthermia or radiofrequency electric field hyperthermia of cancerous cells through green-synthesized curcumin-coated gold nanoparticles. *Lasers Med Sci.* 2022;37(2):1333-1341.DOI:10.1007/s10103-021-03399-7

126. Rodrigues FC, Hari G, Pai KSR, et al. Molecular modeling piloted analysis for semicarbazone derivative of curcumin as a potent Abl-kinase inhibitor targeting colon cancer. *3 Biotech.* 2021;11(12):506.DOI:10.1007/s13205-021-03051-9

127. Romanucci V, Giordano M, Pagano R, et al. Solid-phase synthesis of curcumin mimics and their anticancer activity against human pancreatic, prostate, and colorectal cancer cell lines. *Bioorganic & Medicinal Chemistry.* 2021;42:116249.DOI:10.1016/j.bmc.2021.116249

128. Santana-Gálvez J, Villela-Castrejón J, Serna-Saldívar SO, Cisneros-Zevallos L, Jacobo-Velázquez DA. Synergistic Combinations of Curcumin, Sulforaphane, and Dihydrocaffeic Acid against Human Colon Cancer Cells. *Int J Mol Sci.* 2020;21(9).DOI:10.3390/ijms21093108

129. Sayyed A, Heuertz R, Ezekiel UR. Curcumin, but not its degradation products, in combination with silibinin is primarily responsible for the inhibition of colon cancer cell proliferation. *MicroPubl Biol.* 2022;2022.DOI:10.17912/micropub.biology.000617

130. Seiwert N, Fahrer J, Nagel G, Frank J, Behnam D, Kaina B. Curcumin Administered as Micellar Solution Suppresses Intestinal Inflammation and Colorectal Carcinogenesis. *Nutrition & Cancer.* 2021;73(4):686-693.DOI:10.1080/01635581.2020.1771384

131. Sesarman A, Muntean D, Abrudan B, et al. Improved pharmacokinetics and reduced side effects of doxorubicin therapy by liposomal co-encapsulation with curcumin. *J Liposome Res.* 2021;31(1):1-10.DOI:10.1080/08982104.2019.1682604

132. Shih KC, Chan HW, Wu CY, Chuang HY. Curcumin Enhances the Abscopal Effect in Mice with Colorectal Cancer by Acting as an Immunomodulator. *Pharmaceutics.* 2023;15(5).DOI:10.3390/pharmaceutics15051519

133. Shnaikat SG, Shakya AK, Bardaweel SK. Formulation, development and evaluation of hyaluronic acid-conjugated liposomal nanoparticles loaded with regorafenib and curcumin and their in vitro evaluation on colorectal cancer cell lines. *Saudi Pharm J.* 2024;32(7):102099.DOI:10.1016/j.jsps.2024.102099

134. Slika L, Moubarak A, Borjac J, Baydoun E, Patra D. Preparation of curcumin-poly (allyl amine) hydrochloride based nanocapsules: Piperine in nanocapsules accelerates encapsulation and release of curcumin and effectiveness against colon cancer cells. *Mater Sci Eng C Mater Biol Appl.* 2020;109:110550.DOI:10.1016/j.msec.2019.110550

135. Sood A, Gupta A, Bharadwaj R, Ranganath P, Silverman N, Agrawal G. Biodegradable disulfide crosslinked chitosan/stearic acid nanoparticles for dual drug delivery for colorectal cancer. *Carbohydr Polym.* 2022;294:119833.DOI:10.1016/j.carbpol.2022.119833

136. Sorasitthiyanukarn FN, Muangnoi C, Ratnatilaka Na Bhuket P, Rojsitthisak P, Rojsitthisak P. Chitosan/alginate nanoparticles as a promising approach for oral delivery of curcumin diglutaric acid for cancer treatment. *Mater Sci Eng C Mater Biol Appl.* 2018;93:178-190.DOI:10.1016/j.msec.2018.07.069

137. Sripetthong S, Eze FN, Sajomsang W, Ovatlarnporn C. Development of pH-Responsive N-benzyl-N-O-succinyl Chitosan Micelles Loaded with a Curcumin Analog (Cyqualone) for Treatment of Colon Cancer. *Molecules.* 2023;28(6).DOI:10.3390/molecules28062693

138. Sritharan S, Sivalingam N. Curcumin induced apoptosis is mediated through oxidative stress in mutated p53 and wild type p53 colon adenocarcinoma cell lines. *Journal of Biochemical & Molecular Toxicology.* 2021;35(1):e22616.DOI:10.1002/jbt.22616

139. Sufi SA, Hoda M, Pajaniradje S, Mukherjee V, Coumar SM, Rajagopalan R. Enhanced drug retention, sustained release, and anti-cancer potential of curcumin and indole-curcumin analog-loaded polysorbate 80-stabilizied PLGA nanoparticles in colon cancer cell line SW480. *Int J Pharm.* 2020;588:119738.DOI:10.1016/j.ijpharm.2020.119738

140. Sun J, Zhao Z, Lu J, et al. The Tumor Microenvironment Mediates the HIF-1α/PD-L1 Pathway to Promote Immune Escape in Colorectal Cancer. *Int J Mol Sci.* 2024;25(7).DOI:10.3390/ijms25073735

141. Szafran RG, Gąsiorowski K, Wiatrak B. Colorectal Adenocarcinoma Cell Culture in a Microfluidically Controlled Environment with a Static Molecular Gradient of Polyphenol. *Molecules.* 2021;26(11).DOI:10.3390/molecules26113215

142. Tian Q, Wang X, Song S, An L, Yang S, Huang G. Engineering of an endogenous hydrogen sulfide responsive smart agent for photoacoustic imaging-guided combination of photothermal therapy and chemotherapy for colon cancer. *J Adv Res.* 2022;41:159-168.DOI:10.1016/j.jare.2022.01.018

143. Tiwari A, Bose D, Mishra P, Jain A, Jain SK. Determination of Oxaliplatin and Curcumin in Combination via Micellar HPLC and Its Method Validation. *J AOAC Int.* 2022;105(4):999-1007.DOI:10.1093/jaoacint/qsac042

144. Tong Q, Wu Z. Curcumin inhibits colon cancer malignant progression and promotes T cell killing by regulating miR-206 expression. *Clin Anat.* 2024;37(1):2-11.DOI:10.1002/ca.24057

145. Turgut Y, Yurdakok-Dikmen B, Uyar R, Birer M, Filazi A, Acarturk F. Effects of electrospun fiber curcumin on bisphenol A exposed Caco-2 cells. *Drug & Chemical Toxicology.* 2022;45(6):2613-2625.DOI:10.1080/01480545.2021.1979031

146. Valiveti CK, Kumar B, Singh AD, et al. Stable Dietary Ora-Curcumin Formulation Protects from Experimental Colitis and Colorectal Cancer. *Cells.* 2024;13(11).DOI:10.3390/cells13110957

147. Vukmirovic D, Vo NTK, Seymour C, Rollo D, Mothersill C. Influence of common dietary supplements (curcumin, andrographolide, and d-limonene) on the radiobiological responses of p53-competent colonic cancer epithelial cells. *Int J Radiat Biol.* 2021;97(3):341-347.DOI:10.1080/09553002.2021.1864499

148. Wan C, Ma Q, Anderson S, et al. Effects of Curcuminoids and Surfactant-Formulated Curcumin on Chemo-Resistant Colorectal Cancer. *Am J Chin Med.* 2023;51(6):1577-1594.DOI:10.1142/s0192415x23500714

149. Wang A, Jain S, Dia V, Lenaghan SC, Zhong Q. Shellac Micelles Loaded with Curcumin Using a pH Cycle to Improve Dispersibility, Bioaccessibility, and Potential for Colon Delivery. *Journal of Agricultural & Food Chemistry.* 2022;70(48):15166-15177.DOI:10.1021/acs.jafc.2c04428

150. Wang H, Cai X, Ma L. Curcumin Modifies Epithelial-Mesenchymal Transition in Colorectal Cancer Through Regulation of miR-200c/EPM5. *Cancer Management & Research.* 2020;12:9405-9415.DOI:10.2147/cmar.s260129

151. Wei H, Li X, Liu F, et al. Curcumin inhibits the development of colorectal cancer via regulating the USP4/LAMP3 pathway. *Naunyn Schmiedebergs Arch Pharmacol.* 2024;397(3):1749-1762.DOI:10.1007/s00210-023-02721-0

152. Wei SC, Lin YS, Tsao PN, Wu-Tsai JJ, Wu CH, Wong JM. Comparison of the anti-proliferation and apoptosis-induction activities of sulindac, celecoxib, curcumin, and nifedipine in mismatch repair-deficient cell lines. *J Formos Med Assoc.* 2004;103(8):599-606

153. Wezgowiec J, Tsirigotis-Maniecka M, Saczko J, Wieckiewicz M, Wilk KA. Microparticles vs. Macroparticles as Curcumin Delivery Vehicles: Structural Studies and Cytotoxic Effect in Human Adenocarcinoma Cell Line (LoVo). *Molecules.* 2021;26(19).DOI:10.3390/molecules26196056

154. Wu X, Ueland PM, Roper J, et al. Combined Supplementation with Vitamin B-6 and Curcumin is Superior to Either Agent Alone in Suppressing Obesity-Promoted Colorectal Tumorigenesis in Mice. *J Nutr.* 2021;151(12):3678-3688.DOI:10.1093/jn/nxab320

155. Wu Y, Han Y, Zhao NN, Zhao XF. Curcumin exerts therapeutic effects on colorectal cancer by blocking the cell cycle and regulating apoptosis. *Asian J Surg.* 2024.DOI:10.1016/j.asjsur.2024.08.089

156. Wu Z, Zhao X, Sun Y, Yu H. Curcumin suppresses colorectal cancer development with epithelial-mesenchymal transition via modulating circular RNA HN1/miR-302a-3p/PIK3R3 axis. *Journal of Physiology & Pharmacology.* 2022;73(2).DOI:10.26402/jpp.2022.2.05

157. Wulandari F, Ikawati M, Widyarini S, et al. Tumour-suppressive effects of curcumin analogs CCA-1.1 and Pentagamavunone-1 in colon cancer: In vivo and in vitro studies. *J Adv Pharm Technol Res.* 2023;14(4):317-324.DOI:10.4103/japtr.japtr_315_23

158. Xiang L, He B, Liu Q, et al. Antitumor effects of curcumin on the proliferation, migration and apoptosis of human colorectal carcinoma HCT‑116 cells. *Oncol Rep.* 2020;44(5):1997-2008.DOI:10.3892/or.2020.7765

159. Xiao Y, Guo G, Wang H, et al. Curcumin/L-OHP co-loaded HAP for cGAS-STING pathway activation to enhance the natural immune response in colorectal cancer. *Bioeng Transl Med.* 2024;9(1):e10610.DOI:10.1002/btm2.10610

160. Xin W, Zhang Y. Curcumin activates the JNK signaling pathway to promote ferroptosis in colon cancer cells. *Chem Biol Drug Des.* 2024;103(3):e14468.DOI:10.1111/cbdd.14468

161. Xu C, Liu C. Extracellular vesicles isolated from curcumin-medium weakened RKO cell proliferation and migration. *Transl Cancer Res.* 2024;13(6):2596-2604.DOI:10.21037/tcr-24-98

162. Xu D, Li L, Yu Z. Effect and mechanism of curcumin on colon cancer cell senescence through early growth response 1 (EGR1). *Transl Cancer Res.* 2024;13(7):3251-3261.DOI:10.21037/tcr-24-26

163. Xu L, Wang X, Wang XY, Yao QH, Chen YB. [Curcumin mediates IL-6/STAT3 signaling pathway to repair intestinal mucosal injury induced by 5-FU chemotherapy for colon cancer]. *Zhongguo Zhong Yao Za Zhi.* 2021;46(3):670-677.DOI:10.19540/j.cnki.cjcmm.20201106.401

164. Xu W, Shen Y. Curcumin affects apoptosis of colorectal cancer cells through ATF6-mediated endoplasmic reticulum stress. *Chem Biol Drug Des.* 2024;103(1):e14433.DOI:10.1111/cbdd.14433

165. Yang J, He C, Liu N. Proteomic analysis of the chemosensitizing effect of curcumin on CRC cells treated with 5-FU. *Front Med (Lausanne).* 2022;9:1032256.DOI:10.3389/fmed.2022.1032256

166. Yang Y, Liang S, Geng H, et al. Proteomics revealed the crosstalk between copper stress and cuproptosis, and explored the feasibility of curcumin as anticancer copper ionophore. *Free Radical Biology & Medicine.* 2022;193(Pt 2):638-647.DOI:10.1016/j.freeradbiomed.2022.11.023

167. Yavuz Türel G, Şahin Calapoğlu N, Bayram D, Özgöçmen M, Toğay VA, Evgen Tülüceoğlu E. Curcumin induces apoptosis through caspase dependent pathway in human colon carcinoma cells. *Mol Biol Rep.* 2022;49(2):1351-1360.DOI:10.1007/s11033-021-06965-y

168. Yu C, Jianying L, Han W, Xu W, Juan N. Effects of Curcumin and Soy Isoflavones on Genomic Instability of Human Colon Cells NCM460 and SW620. *Cellular & Molecular Biology (Noisy-Le-Grand, France).* 2023;69(1):36-43.DOI:10.14715/cmb/2022.69.1.7

169. Zhang B, Yan J, Jin Y, Yang Y, Zhao X. Curcumin-shellac nanoparticle-loaded GelMA/SilMA hydrogel for colorectal cancer therapy. *European Journal of Pharmaceutics & Biopharmaceutics.* 2024;202:114409.DOI:10.1016/j.ejpb.2024.114409

170. Zhang D, Jiang L, Liu C. A convergent synthetic platform for polymeric nanoparticle for the treatment of combination colorectal cancer therapy. *J Biomater Sci Polym Ed.* 2021;32(14):1835-1848.DOI:10.1080/09205063.2021.1941556

171. Zhang N, Gao M, Wang Z, et al. Curcumin reverses doxorubicin resistance in colon cancer cells at the metabolic level. *Journal of Pharmaceutical & Biomedical Analysis.* 2021;201:114129.DOI:10.1016/j.jpba.2021.114129

172. Zhang N, Hao Y, Liu H, Yu Q, Bo B, Liang J. Combined anti-cancer effects of curcumin and oxaliplatin on colon carcinoma colo205 cells using transplanted nude mice. *Pak J Pharm Sci.* 2021;34(5(Special)):2021-2025

173. Zhao R, Du S, Liu Y, et al. Mucoadhesive-to-penetrating controllable peptosomes-in-microspheres co-loaded with anti-miR-31 oligonucleotide and Curcumin for targeted colorectal cancer therapy. *Theranostics.* 2020;10(8):3594-3611.DOI:10.7150/thno.40318

174. Zheng X, Yang X, Lin J, Song F, Shao Y. Low curcumin concentration enhances the anticancer effect of 5-fluorouracil against colorectal cancer. *Phytomedicine.* 2021;85:153547.DOI:10.1016/j.phymed.2021.153547

175. Zheng ZH, You HY, Feng YJ, Zhang ZT. LncRNA KCNQ1OT1 is a key factor in the reversal effect of curcumin on cisplatin resistance in the colorectal cancer cells. *Molecular & Cellular Biochemistry.* 2021;476(7):2575-2585.DOI:10.1007/s11010-020-03856-x

176. Zhu C, Fang Z, Peng L, Gao F, Peng W, Song F. Curcumin Suppresses the Progression of Colorectal Cancer by Improving Immunogenic Cell Death Caused by Irinotecan. *Chemotherapy.* 2022;67(4):211-222.DOI:10.1159/000518121

177. Zhou M, Niu H, Cui D, et al. Resveratrol triggers autophagy-related apoptosis to inhibit the progression of colorectal cancer via inhibition of FOXQ1. *Phytother Res.* 2024;38(6):3218-3239.DOI:10.1002/ptr.8184

178. Zhang Z, Ji Y, Hu N, et al. Ferroptosis-induced anticancer effect of resveratrol with a biomimetic nano-delivery system in colorectal cancer treatment. *Asian J Pharm Sci.* 2022;17(5):751-766.DOI:10.1016/j.ajps.2022.07.006

179. Zhang Y, Li Y, Sun C, et al. Effect of Pterostilbene, a Natural Derivative of Resveratrol, in the Treatment of Colorectal Cancer through Top1/Tdp1-Mediated DNA Repair Pathway. *Cancers (Basel).* 2021;13(16).DOI:10.3390/cancers13164002

180. Yu Z, Zhu W, Lu F, et al. Inhibitory effects of resveratrol on platelet activation and thrombosis in colon cancer through regulation of the MAPK and cGMP/VASP pathways. *Thromb Res.* 2024;241:109111.DOI:10.1016/j.thromres.2024.109111

181. Wu KL, Lee KC, Yen CK, Chen CN, Chang SF, Huang WS. Visfatin and Resveratrol Differentially Regulate the Expression of Thymidylate Synthase to Control the Sensitivity of Human Colorectal Cancer Cells to Capecitabine Cytotoxicity. *Life (Basel).* 2021;11(12).DOI:10.3390/life11121371

182. Wang Y, Wang W, Wu X, et al. Resveratrol Sensitizes Colorectal Cancer Cells to Cetuximab by Connexin 43 Upregulation-Induced Akt Inhibition. *Front Oncol.* 2020;10:383.DOI:10.3389/fonc.2020.00383

183. Wang N, Gao E, Cui C, et al. The combined anticancer of peanut skin procyanidins and resveratrol to CACO-2 colorectal cancer cells. *Food Sci Nutr.* 2023;11(10):6483-6497.DOI:10.1002/fsn3.3590

184. Wang C, Wang N, Li N, Yu Q, Wang F. Combined Effects of Resveratrol and Vitamin E From Peanut Seeds and Sprouts on Colorectal Cancer Cells. *Front Pharmacol.* 2021;12:760919.DOI:10.3389/fphar.2021.760919

185. Wada H, Sato Y, Fujimoto S, et al. Resveratrol inhibits development of colorectal adenoma via suppression of LEF1; comprehensive analysis with connectivity map. *Cancer Sci.* 2022;113(12):4374-4384.DOI:10.1111/cas.15576

186. Sun X, Li F, Yuan L, Bing Z, Li X, Yang K. pH-responsive resveratrol-loaded ZIF-8 nanoparticles modified with tannic acid for promoting colon cancer cell apoptosis. *J Biomed Mater Res B Appl Biomater.* 2024;112(1):e35320.DOI:10.1002/jbm.b.35320

187. Sudha T, El-Far AH, Mousa DS, Mousa SA. Resveratrol and Its Nanoformulation Attenuate Growth and the Angiogenesis of Xenograft and Orthotopic Colon Cancer Models. *Molecules.* 2020;25(6).DOI:10.3390/molecules25061412

188. Senthil Kumar C, Thangam R, Mary SA, Kannan PR, Arun G, Madhan B. Targeted delivery and apoptosis induction of trans-resveratrol-ferulic acid loaded chitosan coated folic acid conjugate solid lipid nanoparticles in colon cancer cells. *Carbohydr Polym.* 2020;231:115682.DOI:10.1016/j.carbpol.2019.115682

189. Samprasit W, Opanasopit P, Chamsai B. Alpha-mangostin and resveratrol, dual-drugs-loaded mucoadhesive thiolated chitosan-based nanoparticles for synergistic activity against colon cancer cells. *J Biomed Mater Res B Appl Biomater.* 2022;110(6):1221-1233.DOI:10.1002/jbm.b.34992

190. Ren CP, Zhang YN, Wu YL, Du XX, Cui XL. [Effects of resveratrol on inhibiting pyroptosis of intestinal cancer cells]. *Zhongguo Ying Yong Sheng Li Xue Za Zhi.* 2022;38(4):326-331.DOI:10.12047/j.cjap.6303.2022.062

191. Qin X, Luo H, Deng Y, Yao X, Zhang J, He B. Resveratrol inhibits proliferation and induces apoptosis via the Hippo/YAP pathway in human colon cancer cells. *Biochemical & Biophysical Research Communications.* 2022;636(Pt 1):197-204.DOI:10.1016/j.bbrc.2022.10.077

192. Qian Y, Wang R, Wei W, Wang M, Wang S. Resveratrol reverses the cadmium-promoted migration, invasion, and epithelial-mesenchymal transition procession by regulating the expression of ZEB1. *Human & Experimental Toxicology.* 2021;40(12_suppl):S331-S338.DOI:10.1177/09603271211041678

193. Ochoa-Sanchez A, Sahare P, Pathak S, et al. Evaluation of the synergistic effects of curcumin-resveratrol co-loaded biogenic silica on colorectal cancer cells. *Front Pharmacol.* 2024;15:1341773.DOI:10.3389/fphar.2024.1341773

194. Navarro-Orcajada S, Vidal-Sánchez FJ, Conesa I, Matencio A, López-Nicolás JM. Improvement of the Physicochemical Limitations of Rhapontigenin, a Cytotoxic Analogue of Resveratrol against Colon Cancer. *Biomolecules.* 2023;13(8).DOI:10.3390/biom13081270

195. Muthyalaiah YS, Arockiasamy S, P AA. Exploring the molecular interactions and binding affinity of resveratrol and calcitriol with RAGE and its intracellular proteins and kinases involved in colorectal cancer. *Journal of Biomolecular Structure & Dynamics.* 2023:1-24.DOI:10.1080/07391102.2023.2258993

196. Moreira H, Szyjka A, Grzesik J, et al. Celastrol and Resveratrol Modulate SIRT Genes Expression and Exert Anticancer Activity in Colon Cancer Cells and Cancer Stem-like Cells. *Cancers (Basel).* 2022;14(6).DOI:10.3390/cancers14061372

197. Moreira H, Szyjka A, Bęben D, et al. Genotoxic and Anti-Migratory Effects of Camptothecin Combined with Celastrol or Resveratrol in Metastatic and Stem-like Cells of Colon Cancer. *Cancers (Basel).* 2024;16(19).DOI:10.3390/cancers16193279

198. Md S, Abdullah S, Alhakamy NA, et al. Development, Optimization, and In Vitro Evaluation of Novel Oral Long-Acting Resveratrol Nanocomposite In-Situ Gelling Film in the Treatment of Colorectal Cancer. *Gels.* 2021;7(4).DOI:10.3390/gels7040276

199. Liu H, Zhang L, Hao L, Fan D. Resveratrol Inhibits Colorectal Cancer Cell Tumor Property by Activating the miR-769-5p/MSI1 Pathway. *Mol Biotechnol.* 2024.DOI:10.1007/s12033-024-01167-w

200. Li F, Yan RY, Li KY, et al. [Molecular mechanism of resveratrol combined with irinotecan in treatment of colorectal cancer]. *Zhongguo Zhong Yao Za Zhi.* 2023;48(8):2212-2221.DOI:10.19540/j.cnki.cjcmm.20221201.704

201. Kim N, Kwon J, Shin US, Jung J. Stimulatory Anticancer Effect of Resveratrol Mediated by G Protein-Coupled Estrogen Receptor in Colorectal Cancer. *Biomol Ther (Seoul).* 2023;31(6):655-660.DOI:10.4062/biomolther.2023.072

202. Khayat MT, Zarka MA, El-Telbany DFA, et al. Intensification of resveratrol cytotoxicity, pro-apoptosis, oxidant potentials in human colorectal carcinoma HCT-116 cells using zein nanoparticles. *Sci Rep.* 2022;12(1):15235.DOI:10.1038/s41598-022-18557-2

203. Jozkowiak M, Skupin-Mrugalska P, Nowicki A, et al. The Effect of 4'-hydroxy-3,4,5-trimetoxystilbene, the Metabolite of Resveratrol Analogue DMU-212, on Growth, Cell Cycle and Apoptosis in DLD-1 and LOVO Colon Cancer Cell Lines. *Nutrients.* 2020;12(5).DOI:10.3390/nu12051327

204. Jia M, Tan X, Yuan Z, Zhu W, Yan P. Nanoliposomes Encapsulated Rapamycin/Resveratrol to Induce Apoptosis and Ferroptosis for Enhanced Colorectal Cancer Therapy. *J Pharm Sci.* 2024;113(8):2565-2574.DOI:10.1016/j.xphs.2024.05.015

205. Gündoğdu A, Özyurt R. Resveratrol downregulates ENaCs through the activation of AMPK in human colon cancer cells. *Tissue & Cell.* 2023;82:102071.DOI:10.1016/j.tice.2023.102071

206. Gao X, Zhu Y, Lv T, et al. Resveratrol restrains colorectal cancer metastasis by regulating miR-125b-5p/TRAF6 signaling axis. *Am J Cancer Res.* 2024;14(5):2390-2407.DOI:10.62347/zbvg9125

207. Fu Y, Ye Y, Zhu G, et al. Resveratrol induces human colorectal cancer cell apoptosis by activating the mitochondrial pathway via increasing reactive oxygen species. *Mol Med Rep.* 2021;23(3).DOI:10.3892/mmr.2020.11809

208. Elgizawy HA, Ali AA, Hussein MA. Resveratrol: Isolation, and Its Nanostructured Lipid Carriers, Inhibits Cell Proliferation, Induces Cell Apoptosis in Certain Human Cell Lines Carcinoma and Exerts Protective Effect Against Paraquat-Induced Hepatotoxicity. *J Med Food.* 2021;24(1):89-100.DOI:10.1089/jmf.2019.0286

209. Delmas D, Passilly-Degrace P, Jannin B, Cherkaoui Malki M, Latruffe N. Resveratrol, a chemopreventive agent, disrupts the cell cycle control of human SW480 colorectal tumor cells. *Int J Mol Med.* 2002;10(2):193-199

210. Dariya B, Behera SK, Srivani G, Farran B, Alam A, Nagaraju GP. Computational analysis of nuclear factor-κB and resveratrol in colorectal cancer. *Journal of Biomolecular Structure & Dynamics.* 2021;39(8):2914-2922.DOI:10.1080/07391102.2020.1757511

211. Dariya B, Behera SK, Srivani G, Aliya S, Alam A, Nagaraju GP. Resveratrol binds and activates RKIP protein in colorectal cancer. *Amino Acids.* 2020;52(9):1299-1306.DOI:10.1007/s00726-020-02889-2

212. Dana P, Thumrongsiri N, Tanyapanyachon P, Chonniyom W, Punnakitikashem P, Saengkrit N. Resveratrol Loaded Liposomes Disrupt Cancer Associated Fibroblast Communications within the Tumor Microenvironment to Inhibit Colorectal Cancer Aggressiveness. *Nanomaterials (Basel).* 2022;13(1).DOI:10.3390/nano13010107

213. Czapla J, Drzyzga A, Matuszczak S, et al. The Complex Composition of Trans-resveratrol, Quercetin, Vitamin E and Selenium Inhibits the Growth of Colorectal Carcinoma. *Anticancer Res.* 2022;42(10):4763-4772.DOI:10.21873/anticanres.15981

214. Chang WL, Yang KC, Peng JY, et al. Parecoxib Enhances Resveratrol against Human Colorectal Cancer Cells through Akt and TXNDC5 Inhibition and MAPK Regulation. *Nutrients.* 2024;16(17).DOI:10.3390/nu16173020

215. Cesmeli S, Goker Bagca B, Caglar HO, Ozates NP, Gunduz C, Biray Avci C. Combination of resveratrol and BIBR1532 inhibits proliferation of colon cancer cells by repressing expression of LncRNAs. *Med Oncol.* 2021;39(1):12.DOI:10.1007/s12032-021-01611-w

216. Castrillón-López W, Herrera-Ramírez A, Moreno-Quintero G, Coa JC, Naranjo TW, Cardona-Galeano W. Resveratrol/Hydrazone Hybrids: Synthesis and Chemopreventive Activity against Colorectal Cancer Cells. *Pharmaceutics.* 2022;14(11).DOI:10.3390/pharmaceutics14112278

217. Buhrmann C, Shayan P, Brockmueller A, Shakibaei M. Resveratrol Suppresses Cross-Talk between Colorectal Cancer Cells and Stromal Cells in Multicellular Tumor Microenvironment: A Bridge between In Vitro and In Vivo Tumor Microenvironment Study. *Molecules.* 2020;25(18).DOI:10.3390/molecules25184292

218. Brockmueller A, Shayan P, Shakibaei M. Evidence That β1-Integrin Is Required for the Anti-Viability and Anti-Proliferative Effect of Resveratrol in CRC Cells. *Int J Mol Sci.* 2022;23(9).DOI:10.3390/ijms23094714

219. Brockmueller A, Mueller AL, Shayan P, Shakibaei M. β1-Integrin plays a major role in resveratrol-mediated anti-invasion effects in the CRC microenvironment. *Front Pharmacol.* 2022;13:978625.DOI:10.3389/fphar.2022.978625

220. Brockmueller A, Girisa S, Kunnumakkara AB, Shakibaei M. Resveratrol Modulates Chemosensitisation to 5-FU via β1-Integrin/HIF-1α Axis in CRC Tumor Microenvironment. *Int J Mol Sci.* 2023;24(5).DOI:10.3390/ijms24054988

221. Brockmueller A, Buhrmann C, Shayan P, Shakibaei M. Resveratrol induces apoptosis by modulating the reciprocal crosstalk between p53 and Sirt-1 in the CRC tumor microenvironment. *Front Immunol.* 2023;14:1225530.DOI:10.3389/fimmu.2023.1225530

222. Alrafas HR, Busbee PB, Chitrala KN, Nagarkatti M, Nagarkatti P. Alterations in the Gut Microbiome and Suppression of Histone Deacetylases by Resveratrol Are Associated with Attenuation of Colonic Inflammation and Protection Against Colorectal Cancer. *J Clin Med.* 2020;9(6).DOI:10.3390/jcm9061796

223. Akash S, Islam MR, Bhuiyan AA, et al. In silico evaluation of anti-colorectal cancer inhibitors by Resveratrol derivatives targeting Armadillo repeats domain of APC: molecular docking and molecular dynamics simulation. *Front Oncol.* 2024;14:1360745.DOI:10.3389/fonc.2024.1360745

224. Chen H, Ye C, Cai B, et al. Berberine inhibits intestinal carcinogenesis by suppressing intestinal pro-inflammatory genes and oncogenic factors through modulating gut microbiota. *BMC Cancer.* 2022;22(1):566.DOI:10.1186/s12885-022-09635-9

225. Chen H, Ye C, Wu C, et al. Berberine inhibits high fat diet-associated colorectal cancer through modulation of the gut microbiota-mediated lysophosphatidylcholine. *Int J Biol Sci.* 2023;19(7):2097-2113.DOI:10.7150/ijbs.81824

226. Chen YX, Gao QY, Zou TH, et al. Berberine versus placebo for the prevention of recurrence of colorectal adenoma: a multicentre, double-blinded, randomised controlled study. *Lancet Gastroenterol Hepatol.* 2020;5(3):267-275.DOI:10.1016/s2468-1253(19)30409-1

227. Deng J, Zhao L, Yuan X, et al. Pre-Administration of Berberine Exerts Chemopreventive Effects in AOM/DSS-Induced Colitis-Associated Carcinogenesis Mice via Modulating Inflammation and Intestinal Microbiota. *Nutrients.* 2022;14(4).DOI:10.3390/nu14040726

228. Gong C, Hu X, Xu Y, et al. Berberine inhibits proliferation and migration of colorectal cancer cells by downregulation of GRP78. *Anticancer Drugs.* 2020;31(2):141-149.DOI:10.1097/cad.0000000000000835

229. Guan X, Zheng X, Vong CT, et al. Combined effects of berberine and evodiamine on colorectal cancer cells and cardiomyocytes in vitro. *Eur J Pharmacol.* 2020;875:173031.DOI:10.1016/j.ejphar.2020.173031

230. Gui Z, Li J, Li J, et al. Berberine promotes IGF2BP3 ubiquitination by TRIM21 to induce G1/S phase arrest in colorectal cancer cells. *Chem Biol Interact.* 2023;374:110408.DOI:10.1016/j.cbi.2023.110408

231. Ibrahim D, Khater SI, Abdelfattah-Hassan A, et al. Prospects of new targeted nanotherapy combining liponiosomes with berberine to combat colorectal cancer development: An in vivo experimental model. *Int J Pharm.* 2023;647:123511.DOI:10.1016/j.ijpharm.2023.123511

232. Kwon S, Chan AT. Extracting the benefits of berberine for colorectal cancer. *Lancet Gastroenterol Hepatol.* 2020;5(3):231-233.DOI:10.1016/s2468-1253(19)30430-3

233. Li G, Zhang C, Liang W, Zhang Y, Shen Y, Tian X. Berberine regulates the Notch1/PTEN/PI3K/AKT/mTOR pathway and acts synergistically with 17-AAG and SAHA in SW480 colon cancer cells. *Pharm Biol.* 2021;59(1):21-30.DOI:10.1080/13880209.2020.1865407

234. Li SY, Li Y, Wu ZH, et al. Study on the mechanism of action of effective monomeric, berberine of Xianglian Pill in inhibiting human colon cancer cells based on fatty acid synthase target. *J Tradit Complement Med.* 2023;13(6):538-549.DOI:10.1016/j.jtcme.2023.05.008

235. Li SY, Shi CJ, Fu WM, Zhang JF. Berberine inhibits tumour growth in vivo and in vitro through suppressing the lincROR-Wnt/β-catenin regulatory axis in colorectal cancer. *Journal of Pharmacy & Pharmacology.* 2023;75(1):129-138.DOI:10.1093/jpp/rgac067

236. Ling Q, Fang J, Zhai C, et al. Berberine induces SOCS1 pathway to reprogram the M1 polarization of macrophages via miR-155-5p in colitis-associated colorectal cancer. *Eur J Pharmacol.* 2023;949:175724.DOI:10.1016/j.ejphar.2023.175724

237. Liu Y, Fang X, Li Y, et al. Berberine suppresses the migration and invasion of colon cancer cells by inhibition of lipogenesis through modulation of promyelocytic leukemia zinc finger-mediated sterol-regulatory element binding proteins cleavage-activating protein ubiquitination. *Journal of Pharmacy & Pharmacology.* 2022;74(9):1353-1363.DOI:10.1093/jpp/rgac026

238. Liu Y, Hua W, Li Y, et al. Berberine suppresses colon cancer cell proliferation by inhibiting the SCAP/SREBP-1 signaling pathway-mediated lipogenesis. *Biochem Pharmacol.* 2020;174:113776.DOI:10.1016/j.bcp.2019.113776

239. Nathani S, Mishra R, Katiyar P, Sircar D, Roy P. Zinc Acts Synergistically with Berberine for Enhancing Its Efficacy as an Anti-cancer Agent by Inducing Clusterin-Dependent Apoptosis in HT-29 Colorectal Cancer Cells. *Biol Trace Elem Res.* 2023;201(8):3755-3773.DOI:10.1007/s12011-022-03460-8

240. Ni L, Sun P, Ai M, Kong L, Xu R, Li J. Berberine inhibited the formation of metastasis by intervening the secondary homing of colorectal cancer cells in the blood circulation to the lung and liver through HEY2. *Phytomedicine.* 2022;104:154303.DOI:10.1016/j.phymed.2022.154303

241. Nie Q, Peng WW, Wang Y, Zhong L, Zhang X, Zeng L. β-catenin correlates with the progression of colon cancers and berberine inhibits the proliferation of colon cancer cells by regulating the β-catenin signaling pathway. *Gene.* 2022;818:146207.DOI:10.1016/j.gene.2022.146207

242. Ning H, Lu W, Jia Q, et al. Discovery of oxyepiberberine as a novel tubulin polymerization inhibitor and an anti-colon cancer agent against LS-1034 cells. *Invest New Drugs.* 2021;39(2):386-393.DOI:10.1007/s10637-020-01006-0

243. Okuno K, Garg R, Yuan YC, Tokunaga M, Kinugasa Y, Goel A. Berberine and Oligomeric Proanthocyanidins Exhibit Synergistic Efficacy Through Regulation of PI3K-Akt Signaling Pathway in Colorectal Cancer. *Front Oncol.* 2022;12:855860.DOI:10.3389/fonc.2022.855860

244. Othman MS, Al-Bagawi AH, Obeidat ST, Fareid MA, Habotta OA, Moneim AEA. Antitumor Activity of Zinc Nanoparticles Synthesized with Berberine on Human Epithelial Colorectal Adenocarcinoma (Caco-2) Cells through Acting on Cox-2/NF-kB and p53 Pathways. *Anticancer Agents Med Chem.* 2022;22(10):2002-2010.DOI:10.2174/1871520621666211004115839

245. Qian Y, Kang Z, Zhao L, et al. Berberine might block colorectal carcinogenesis by inhibiting the regulation of B-cell function by Veillonella parvula. *Chin Med J (Engl).* 2023;136(22):2722-2731.DOI:10.1097/cm9.0000000000002752

246. Samad MA, Saiman MZ, Abdul Majid N, Karsani SA, Yaacob JS. Berberine Inhibits Telomerase Activity and Induces Cell Cycle Arrest and Telomere Erosion in Colorectal Cancer Cell Line, HCT 116. *Molecules.* 2021;26(2).DOI:10.3390/molecules26020376

247. Samad MA, Saiman MZ, Abdul Majid N, Karsani SA, Yaacob JS. Berberine and RNAi-Targeting Telomerase Reverse Transcriptase (TERT) and/or Telomerase RNA Component (TERC) Caused Oxidation in Colorectal Cancer Cell Line, HCT 116: An Integrative Approach using Molecular and Metabolomic Studies. *Cell Biochemistry & Biophysics.* 2024;82(1):153-173.DOI:10.1007/s12013-023-01210-8

248. Sun Q, Shan R, Qi T, Yang P. Berberine Reverses the Tumorigenic Function of Colon Cancer Cell-Derived Exosomes. *Tohoku J Exp Med.* 2023;260(1):75-85.DOI:10.1620/tjem.2022.J119

249. Sun Q, Tao Q, Ming T, et al. Berberine is a suppressor of Hedgehog signaling cascade in colorectal cancer. *Phytomedicine.* 2023;114:154792.DOI:10.1016/j.phymed.2023.154792

250. Sun Q, Yang H, Liu M, et al. Berberine suppresses colorectal cancer by regulation of Hedgehog signaling pathway activity and gut microbiota. *Phytomedicine.* 2022;103:154227.DOI:10.1016/j.phymed.2022.154227

251. Tarawneh N, Hamadneh L, Abu-Irmaileh B, Shraideh Z, Bustanji Y, Abdalla S. Berberine Inhibited Growth and Migration of Human Colon Cancer Cell Lines by Increasing Phosphatase and Tensin and Inhibiting Aquaporins 1, 3 and 5 Expressions. *Molecules.* 2023;28(9).DOI:10.3390/molecules28093823

252. Tong M, Liu H, Hao J, Fan D. Comparative pharmacoproteomics reveals potential targets for berberine, a promising therapy for colorectal cancer. *Biochemical & Biophysical Research Communications.* 2020.DOI:10.1016/j.bbrc.2020.02.052

253. Wang M, Ma Y, Yu G, et al. Integration of microbiome, metabolomics and transcriptome for in-depth understanding of berberine attenuates AOM/DSS-induced colitis-associated colorectal cancer. *Biomedicine & Pharmacotherapy.* 2024;179:117292.DOI:10.1016/j.biopha.2024.117292

254. Wang X, Peng A, Huang C. Suppression of colon cancer growth by berberine mediated by the intestinal microbiota and the suppression of DNA methyltransferases (DNMTs). *Molecular & Cellular Biochemistry.* 2024;479(8):2131-2141.DOI:10.1007/s11010-023-04836-7

255. Wu C, Liu Y, Liu W, et al. NNMT-DNMT1 Axis is Essential for Maintaining Cancer Cell Sensitivity to Oxidative Phosphorylation Inhibition. *Adv Sci (Weinh).* 2022;10(1):e2202642.DOI:10.1002/advs.202202642

256. Xu B, Jiang X, Xiong J, et al. Structure-Activity Relationship Study Enables the Discovery of a Novel Berberine Analogue as the RXRα Activator to Inhibit Colon Cancer. *J Med Chem.* 2020;63(11):5841-5855.DOI:10.1021/acs.jmedchem.0c00088

257. Yan S, Chang J, Hao X, et al. Berberine regulates short-chain fatty acid metabolism and alleviates the colitis-associated colorectal tumorigenesis through remodeling intestinal flora. *Phytomedicine.* 2022;102:154217.DOI:10.1016/j.phymed.2022.154217

258. Yan SH, Hu LM, Hao XH, et al. Chemoproteomics reveals berberine directly binds to PKM2 to inhibit the progression of colorectal cancer. *iScience.* 2022;25(8):104773.DOI:10.1016/j.isci.2022.104773

259. Yang W, Yang T, Huang B, Chen Z, Liu H, Huang C. Berberine improved the microbiota in lung tissue of colon cancer and reversed the bronchial epithelial cell changes caused by cancer cells. *Heliyon.* 2024;10(2):e24405.DOI:10.1016/j.heliyon.2024.e24405

260. Yuan S, Zhang T, Wu Y, Lu Y, Chang F, Zhu Y. Cost-Utility Analysis of Berberine Chemoprevention for Colorectal Cancer After Polypectomy. *Cureus.* 2024;16(5):e61030.DOI:10.7759/cureus.61030

261. Yue B, Gao R, Lv C, et al. Berberine Improves Irinotecan-Induced Intestinal Mucositis Without Impairing the Anti-colorectal Cancer Efficacy of Irinotecan by Inhibiting Bacterial β-glucuronidase. *Front Pharmacol.* 2021;12:774560.DOI:10.3389/fphar.2021.774560

262. Zhang Y, Liu X, Yu M, et al. Berberine inhibits proliferation and induces G0/G1 phase arrest in colorectal cancer cells by downregulating IGF2BP3. *Life Sci.* 2020;260:118413.DOI:10.1016/j.lfs.2020.118413

263. Zhao Y, Roy S, Wang C, Goel A. A Combined Treatment with Berberine and Andrographis Exhibits Enhanced Anti-Cancer Activity through Suppression of DNA Replication in Colorectal Cancer. *Pharmaceuticals (Basel).* 2022;15(3).DOI:10.3390/ph15030262

264. Long L, Xiong W, Lin F, et al. Regulating lactate-related immunometabolism and EMT reversal for colorectal cancer liver metastases using shikonin targeted delivery. *Journal of Experimental & Clinical Cancer Research.* 2023;42(1):117.DOI:10.1186/s13046-023-02688-z

265. Zhou J, Jiang Z, Sun R, et al. Comparison of cell delivery and cell membrane camouflaged PLGA nanoparticles in the delivery of shikonin for colorectal cancer treatment. *Colloids Surf B Biointerfaces.* 2024;241:114017.DOI:10.1016/j.colsurfb.2024.114017

266. Zhang Z, Shen C, Zhou F, Zhang Y. Shikonin potentiates therapeutic efficacy of oxaliplatin through reactive oxygen species-mediated intrinsic apoptosis and endoplasmic reticulum stress in oxaliplatin-resistant colorectal cancer cells. *Drug Dev Res.* 2023;84(3):542-555.DOI:10.1002/ddr.22044

267. Zhang N, Peng F, Wang Y, et al. Shikonin induces colorectal carcinoma cells apoptosis and autophagy by targeting galectin-1/JNK signaling axis. *Int J Biol Sci.* 2020;16(1):147-161.DOI:10.7150/ijbs.36955

268. Zeng X, Sun L, Ling X, et al. Comprehensive analysis identifies novel targets of gemcitabine to improve chemotherapy treatment strategies for colorectal cancer. *Front Endocrinol (Lausanne).* 2023;14:1170526.DOI:10.3389/fendo.2023.1170526

269. Wang Z, Cui Q, Shi L, et al. Network Pharmacology-based Prediction and Verification of Shikonin for Treating Colorectal Cancer. *Recent Pat Anticancer Drug Discov.* 2022;17(3):297-311.DOI:10.2174/1574892817666211224142100

270. Shilnikova K, Piao MJ, Kang KA, et al. Natural Compound Shikonin Induces Apoptosis and Attenuates Epithelial to Mesenchymal Transition in Radiation-Resistant Human Colon Cancer Cells. *Biomol Ther (Seoul).* 2022;30(2):137-144.DOI:10.4062/biomolther.2021.088

271. Shi W, Men L, Pi X, et al. Shikonin suppresses colon cancer cell growth and exerts synergistic effects by regulating ADAM17 and the IL‑6/STAT3 signaling pathway. *Int J Oncol.* 2021;59(6).DOI:10.3892/ijo.2021.5279

272. Qi H, Zhang X, Liu H, et al. Shikonin induced Apoptosis Mediated by Endoplasmic Reticulum Stress in Colorectal Cancer Cells. *J Cancer.* 2022;13(1):243-252.DOI:10.7150/jca.65297

273. Piao MJ, Han X, Kang KA, Fernando P, Herath H, Hyun JW. The Endoplasmic Reticulum Stress Response Mediates Shikonin-Induced Apoptosis of 5-Fluorouracil-Resistant Colorectal Cancer Cells. *Biomol Ther (Seoul).* 2022;30(3):265-273.DOI:10.4062/biomolther.2021.118

274. Ma Y, Lai X, Wen Z, et al. Design, synthesis and biological evaluation of novel modified dual-target shikonin derivatives for colorectal cancer treatment. *Bioorg Chem.* 2023;139:106703.DOI:10.1016/j.bioorg.2023.106703

275. Lu Y, Zhou H, Han C, et al. Enhanced therapeutic impact of Shikonin-encapsulated exosomes in the inhibition of colorectal cancer progression. *Nanotechnology.* 2024;35(41).DOI:10.1088/1361-6528/ad61f2

276. Lin H, Ma X, Yang X, et al. Natural shikonin and acetyl-shikonin improve intestinal microbial and protein composition to alleviate colitis-associated colorectal cancer. *Int Immunopharmacol.* 2022;111:109097.DOI:10.1016/j.intimp.2022.109097

277. Li J, Cai W, Yu J, et al. Autophagy inhibition recovers deficient ICD-based cancer immunotherapy. *Biomaterials.* 2022;287:121651.DOI:10.1016/j.biomaterials.2022.121651

278. Hu Z, Zhou X, Zeng D, Lai J. Shikonin induces cell autophagy via modulating the microRNA -545-3p/guanine nucleotide binding protein beta polypeptide 1 axis, thereby disrupting cellular carcinogenesis in colon cancer. *Bioengineered.* 2022;13(3):5928-5941.DOI:10.1080/21655979.2021.2024638

279. Daneshmehr M, Pazhang M, Mollaei S, Ebadi M, Pazhang Y. Targeted delivery of 5-fluorouracil and shikonin by blended and coated chitosan/pectin nanoparticles for treatment of colon cancer. *Int J Biol Macromol.* 2024;270(Pt 2):132413.DOI:10.1016/j.ijbiomac.2024.132413

280. Chen Y, Si L, Zhang J, et al. Uncovering the antitumor effects and mechanisms of Shikonin against colon cancer on comprehensive analysis. *Phytomedicine.* 2021;82:153460.DOI:10.1016/j.phymed.2021.153460

281. Chen Y, Ni J, Gao Y, et al. Integrated proteomics and metabolomics reveals the comprehensive characterization of antitumor mechanism underlying Shikonin on colon cancer patient-derived xenograft model. *Sci Rep.* 2020;10(1):14092.DOI:10.1038/s41598-020-71116-5

282. Chen Y, Gao Y, Yi X, Zhang J, Chen Z, Wu Y. Integration of Transcriptomics and Metabolomics Reveals the Antitumor Mechanism Underlying Shikonin in Colon Cancer. *Front Pharmacol.* 2020;11:544647.DOI:10.3389/fphar.2020.544647

283. Chen J, Liu J, Liu X, et al. Shikonin improves the effectiveness of PD-1 blockade in colorectal cancer by enhancing immunogenicity via Hsp70 upregulation. *Mol Biol Rep.* 2024;51(1):86.DOI:10.1007/s11033-023-09056-2

284. Yu Y, Chen D, Wu T, et al. Dihydroartemisinin enhances the anti-tumor activity of oxaliplatin in colorectal cancer cells by altering PRDX2-reactive oxygen species-mediated multiple signaling pathways. *Phytomedicine.* 2022;98:153932.DOI:10.1016/j.phymed.2022.153932

285. Chellan P, Avery VM, Duffy S, et al. Bioactive half-sandwich Rh and Ir bipyridyl complexes containing artemisinin. *J Inorg Biochem.* 2021;219:111408.DOI:10.1016/j.jinorgbio.2021.111408

286. Yi YC, Liang R, Chen XY, et al. Dihydroartemisinin Suppresses the Tumorigenesis and Cycle Progression of Colorectal Cancer by Targeting CDK1/CCNB1/PLK1 Signaling. *Front Oncol.* 2021;11:768879.DOI:10.3389/fonc.2021.768879

287. Wang Y, Yang Z, Zhu W, et al. Dihydroartemisinin inhibited stem cell-like properties and enhanced oxaliplatin sensitivity of colorectal cancer via AKT/mTOR signaling. *Drug Dev Res.* 2023;84(5):988-998.DOI:10.1002/ddr.22067

288. Elhassanny AEM, Soliman E, Marie M, et al. Heme-Dependent ER Stress Apoptosis: A Mechanism for the Selective Toxicity of the Dihydroartemisinin, NSC735847, in Colorectal Cancer Cells. *Front Oncol.* 2020;10:965.DOI:10.3389/fonc.2020.00965

289. Wang X, Zheng Y, Chai Z, et al. Dihydroartemisinin synergistically enhances the cytotoxic effects of oxaliplatin in colon cancer by targeting the PHB2-RCHY1 mediated signaling pathway. *Mol Carcinog.* 2023;62(3):293-302.DOI:10.1002/mc.23486

290. Wang CZ, Wan C, Luo Y, et al. Effects of dihydroartemisinin, a metabolite of artemisinin, on colon cancer chemoprevention and adaptive immune regulation. *Mol Biol Rep.* 2022;49(4):2695-2709.DOI:10.1007/s11033-021-07079-1

291. Bai B, Wu F, Ying K, et al. Therapeutic effects of dihydroartemisinin in multiple stages of colitis-associated colorectal cancer. *Theranostics.* 2021;11(13):6225-6239.DOI:10.7150/thno.55939

292. Dai X, Chen W, Qiao Y, et al. Dihydroartemisinin inhibits the development of colorectal cancer by GSK-3β/TCF7/MMP9 pathway and synergies with capecitabine. *Cancer Lett.* 2024;582:216596.DOI:10.1016/j.canlet.2023.216596

293. Hu X, Fatima S, Chen M, et al. Dihydroartemisinin is potential therapeutics for treating late-stage CRC by targeting the elevated c-Myc level. *Cell Death Dis.* 2021;12(11):1053.DOI:10.1038/s41419-021-04247-w

294. Bader S, Wilmers J, Ontikatze T, Ritter V, Jendrossek V, Rudner J. Loss of pro-apoptotic Bax and Bak increases resistance to dihydroartemisinin-mediated cytotoxicity in normoxia but not in hypoxia in HCT116 colorectal cancer cells. *Free Radical Biology & Medicine.* 2021;174:157-170.DOI:10.1016/j.freeradbiomed.2021.08.012

295. Bader S, Wilmers J, Pelzer M, Jendrossek V, Rudner J. Activation of anti-oxidant Keap1/Nrf2 pathway modulates efficacy of dihydroartemisinin-based monotherapy and combinatory therapy with ionizing radiation. *Free Radical Biology & Medicine.* 2021;168:44-54.DOI:10.1016/j.freeradbiomed.2021.03.024

296. Wang CZ, Wan C, Li CH, et al. Ruthenium-dihydroartemisinin complex: a promising new compound for colon cancer prevention via G1 cell cycle arrest, apoptotic induction, and adaptive immune regulation. *Cancer Chemotherapy & Pharmacology.* 2024;93(5):411-425.DOI:10.1007/s00280-023-04623-7

297. Su Q, Wang Z, Li P, Wei X, Xiao J, Duan X. pH and ROS Dual-Responsive Autocatalytic Release System Potentiates Immunotherapy of Colorectal Cancer. *Adv Healthc Mater.* 2024:e2401126.DOI:10.1002/adhm.202401126

298. Phung CD, Le TG, Nguyen VH, et al. PEGylated-Paclitaxel and Dihydroartemisinin Nanoparticles for Simultaneously Delivering Paclitaxel and Dihydroartemisinin to Colorectal Cancer. *Pharm Res.* 2020;37(7):129.DOI:10.1007/s11095-020-02819-7

299. Peng J, Wang Q, Zhou J, et al. Targeted Lipid Nanoparticles Encapsulating Dihydroartemisinin and Chloroquine Phosphate for Suppressing the Proliferation and Liver Metastasis of Colorectal Cancer. *Front Pharmacol.* 2021;12:720777.DOI:10.3389/fphar.2021.720777

300. Otto-Ślusarczyk D, Mielczarek-Puta M, Graboń W. The Real Cytotoxic Effect of Artemisinins on Colon Cancer Cells in a Physiological Cell Culture Setting. How Composition of the Culture Medium Biases Experimental Findings. *Pharmaceuticals (Basel).* 2021;14(10).DOI:10.3390/ph14100976

301. Han W, Duan X, Ni K, Li Y, Chan C, Lin W. Co-delivery of dihydroartemisinin and pyropheophorbide-iron elicits ferroptosis to potentiate cancer immunotherapy. *Biomaterials.* 2022;280:121315.DOI:10.1016/j.biomaterials.2021.121315

302. Zhou Y, Liu J, Ma S, et al. Fabrication of polymeric sorafenib coated chitosan and fucoidan nanoparticles: Investigation of anticancer activity and apoptosis in colorectal cancer cells. *Heliyon.* 2024;10(14):e34316.DOI:10.1016/j.heliyon.2024.e34316

303. Zhang Y, Liu J, Mao G, et al. Sargassum fusiforme fucoidan alleviates diet-induced insulin resistance by inhibiting colon-derived ceramide biosynthesis. *Food Funct.* 2021;12(18):8440-8453.DOI:10.1039/d1fo01272j

304. Zhang W, An EK, Park HB, et al. Ecklonia cava fucoidan has potential to stimulate natural killer cells in vivo. *Int J Biol Macromol.* 2021;185:111-121.DOI:10.1016/j.ijbiomac.2021.06.045

305. Xue M, Liang H, Ji X, et al. Effects of fucoidan on gut flora and tumor prevention in 1,2-dimethylhydrazine-induced colorectal carcinogenesis. *J Nutr Biochem.* 2020;82:108396.DOI:10.1016/j.jnutbio.2020.108396

306. Tsai HL, Yeh YS, Chen PJ, et al. The Auxiliary Effects of Low-Molecular-Weight Fucoidan in Locally Advanced Rectal Cancer Patients Receiving Neoadjuvant Concurrent Chemoradiotherapy Before Surgery: A Double-Blind, Randomized, Placebo-Controlled Study. *Integr Cancer Ther.* 2023;22:15347354231187153.DOI:10.1177/15347354231187153

307. Shin YK, Park YR, Lee H, Choi Y, Eom JB. Real-Time Monitoring of Colorectal Cancer Location and Lymph Node Metastasis and Photodynamic Therapy Using Fucoidan-Based Therapeutic Nanogel and Near-Infrared Fluorescence Diagnostic-Therapy System. *Pharmaceutics.* 2023;15(3).DOI:10.3390/pharmaceutics15030930

308. Mabate B, Daub CD, Pletschke BI, Edkins AL. Comparative Analyses of Fucoidans from South African Brown Seaweeds That Inhibit Adhesion, Migration, and Long-Term Survival of Colorectal Cancer Cells. *Mar Drugs.* 2023;21(4).DOI:10.3390/md21040203

309. Liu J, Meng Y, Li B, et al. Ferroptosis-related biotargets and network mechanisms of fucoidan against colorectal cancer: An integrated bioinformatic and experimental approach. *Int J Biol Macromol.* 2022;222(Pt A):1522-1530.DOI:10.1016/j.ijbiomac.2022.09.255

310. Li X, Xin S, Zheng X, et al. Inhibition of the Occurrence and Development of Inflammation-Related Colorectal Cancer by Fucoidan Extracted from Sargassum fusiforme. *Journal of Agricultural & Food Chemistry.* 2022;70(30):9463-9476.DOI:10.1021/acs.jafc.2c02357

311. Khan SH, Anees M, Zofair SFF, et al. Fucoidan based polymeric nanoparticles encapsulating epirubicin: A novel and effective chemotherapeutic formulation against colorectal cancer. *Int J Pharm.* 2024;664:124622.DOI:10.1016/j.ijpharm.2024.124622

312. Huang CW, Chen YC, Yin TC, et al. Low-Molecular-Weight Fucoidan as Complementary Therapy of Fluoropyrimidine-Based Chemotherapy in Colorectal Cancer. *Int J Mol Sci.* 2021;22(15).DOI:10.3390/ijms22158041

313. DuRoss AN, Landry MR, Thomas CR, Jr., Neufeld MJ, Sun C. Fucoidan-coated nanoparticles target radiation-induced P-selectin to enhance chemoradiotherapy in murine colorectal cancer. *Cancer Lett.* 2021;500:208-219.DOI:10.1016/j.canlet.2020.11.021

314. Deng Z, Wu N, Suo Q, et al. Fucoidan, as an immunostimulator promotes M1 macrophage differentiation and enhances the chemotherapeutic sensitivity of capecitabine in colon cancer. *Int J Biol Macromol.* 2022;222(Pt A):562-572.DOI:10.1016/j.ijbiomac.2022.09.201

315. Chan CH, Deng YH, Peng BY, et al. Anti-Colorectal Cancer Effects of Fucoidan Complex-Based Functional Beverage Through Retarding Proliferation, Cell Cycle and Epithelial-Mesenchymal Transition Signaling Pathways. *Integr Cancer Ther.* 2023;22:15347354231213613.DOI:10.1177/15347354231213613

316. Bai X, Wang Y, Hu B, et al. Fucoidan Induces Apoptosis of HT-29 Cells via the Activation of DR4 and Mitochondrial Pathway. *Mar Drugs.* 2020;18(4).DOI:10.3390/md18040220

317. Al Monla R, Dassouki Z, Sari-Chmayssem N, Mawlawi H, Gali-Muhtasib H. Fucoidan and Alginate from the Brown Algae Colpomenia sinuosa and Their Combination with Vitamin C Trigger Apoptosis in Colon Cancer. *Molecules.* 2022;27(2).DOI:10.3390/molecules27020358

318. Zheng Y, Li L, Chen H, et al. Luteolin exhibits synergistic therapeutic efficacy with erastin to induce ferroptosis in colon cancer cells through the HIC1-mediated inhibition of GPX4 expression. *Free Radical Biology & Medicine.* 2023;208:530-544.DOI:10.1016/j.freeradbiomed.2023.09.014

319. Zhang W, Zhang F. Exploration of the mechanism of luteolin against colorectal cancer based on network pharmacology and experimental validation. *Asian J Surg.* 2024.DOI:10.1016/j.asjsur.2024.08.261

320. Yoo HS, Won SB, Kwon YH. Luteolin Induces Apoptosis and Autophagy in HCT116 Colon Cancer Cells via p53-Dependent Pathway. *Nutrition & Cancer.* 2022;74(2):677-686.DOI:10.1080/01635581.2021.1903947

321. Yang C, Wu L, Jin X, et al. Decrease in GPSM2 mediated by the natural product luteolin contributes to colon adenocarcinoma treatment and increases the sensitivity to fluorouracil. *Biomedicine & Pharmacotherapy.* 2024;176:116847.DOI:10.1016/j.biopha.2024.116847

322. Song Y, Yu J, Li L, et al. Luteolin impacts deoxyribonucleic acid repair by modulating the mitogen-activated protein kinase pathway in colorectal cancer. *Bioengineered.* 2022;13(4):10998-11011.DOI:10.1080/21655979.2022.2066926

323. Potočnjak I, Šimić L, Gobin I, Vukelić I, Domitrović R. Antitumor activity of luteolin in human colon cancer SW620 cells is mediated by the ERK/FOXO3a signaling pathway. *Toxicol In Vitro.* 2020;66:104852.DOI:10.1016/j.tiv.2020.104852

324. Pérez-Valero Á, Magadán-Corpas P, Ye S, et al. Antitumor Effect and Gut Microbiota Modulation by Quercetin, Luteolin, and Xanthohumol in a Rat Model for Colorectal Cancer Prevention. *Nutrients.* 2024;16(8).DOI:10.3390/nu16081161

325. Özerkan D. The Determination of Cisplatin and Luteolin Synergistic Effect on Colorectal Cancer Cell Apoptosis and Mitochondrial Dysfunction by Fluorescence Labelling. *J Fluoresc.* 2023;33(3):1217-1225.DOI:10.1007/s10895-023-03145-y

326. Monti E, Marras E, Prini P, Gariboldi MB. Luteolin impairs hypoxia adaptation and progression in human breast and colon cancer cells. *Eur J Pharmacol.* 2020;881:173210.DOI:10.1016/j.ejphar.2020.173210

327. Jang CH, Moon N, Lee J, Kwon MJ, Oh J, Kim JS. Luteolin Synergistically Enhances Antitumor Activity of Oxaliplatin in Colorectal Carcinoma via AMPK Inhibition. *Antioxidants (Basel).* 2022;11(4).DOI:10.3390/antiox11040626

328. Hu Y, Chen X, Li Z, Zheng S, Cheng Y. Thermosensitive In Situ Gel Containing Luteolin Micelles is a Promising Efficient Agent for Colorectal Cancer Peritoneal Metastasis Treatment. *J Biomed Nanotechnol.* 2020;16(1):54-64.DOI:10.1166/jbn.2020.2870

329. Ganai SA, Sheikh FA, Baba ZA, Mir MA, Mantoo MA, Yatoo MA. Anticancer activity of the plant flavonoid luteolin against preclinical models of various cancers and insights on different signalling mechanisms modulated. *Phytother Res.* 2021;35(7):3509-3532.DOI:10.1002/ptr.7044

330. Erdoğan MK, Ağca CA, Aşkın H. Quercetin and Luteolin Improve the Anticancer Effects of 5-Fluorouracil in Human Colorectal Adenocarcinoma In Vitro Model: A Mechanistic Insight. *Nutrition & Cancer.* 2022;74(2):660-676.DOI:10.1080/01635581.2021.1900301

331. Boeing T, Speca S, de Souza P, et al. The PPARγ-dependent effect of flavonoid luteolin against damage induced by the chemotherapeutic irinotecan in human intestinal cells. *Chem Biol Interact.* 2022;351:109712.DOI:10.1016/j.cbi.2021.109712

332. Khan I, Mahfooz S, Saeed M, Ahmad I, Ansari IA. Andrographolide Inhibits Proliferation of Colon Cancer SW-480 Cells via Downregulating Notch Signaling Pathway. *Anticancer Agents Med Chem.* 2021;21(4):487-497.DOI:10.2174/1871520620666200717143109

333. Khan I, Mahfooz S, Ansari IA. Antiproliferative and Apoptotic Properties of Andrographolide Against Human Colon Cancer DLD1 Cell Line. *Endocr Metab Immune Disord Drug Targets.* 2020;20(6):930-942.DOI:10.2174/1871530319666191125111920

334. Khan I, Mahfooz S, Faisal M, Alatar AA, Ansari IA. Andrographolide Induces Apoptosis and Cell Cycle Arrest through Inhibition of Aberrant Hedgehog Signaling Pathway in Colon Cancer Cells. *Nutrition & Cancer.* 2021;73(11-12):2428-2446.DOI:10.1080/01635581.2020.1828942

335. Sharda N, Ikuse T, Hill E, et al. Impact of Andrographolide and Melatonin Combinatorial Drug Therapy on Metastatic Colon Cancer Cells and Organoids. *Clin Med Insights Oncol.* 2021;15:11795549211012672.DOI:10.1177/11795549211012672

336. Hong H, Cao W, Wang Q, Liu C, Huang C. Synergistic antitumor effect of Andrographolide and cisplatin through ROS-mediated ER stress and STAT3 inhibition in colon cancer. *Med Oncol.* 2022;39(5):101.DOI:10.1007/s12032-022-01691-2

337. Yue Z, Zhu Y, Chen T, et al. Bletilla striata polysaccharide-coated andrographolide nanomicelles for targeted drug delivery to enhance anti-colon cancer efficacy. *Front Immunol.* 2024;15:1380229.DOI:10.3389/fimmu.2024.1380229

338. Xu L, Cai P, Li X, et al. Inhibition of NLRP3 inflammasome activation in myeloid-derived suppressor cells by andrographolide sulfonate contributes to 5-FU sensitization in mice. *Toxicology & Applied Pharmacology.* 2021;428:115672.DOI:10.1016/j.taap.2021.115672

339. Sokolov D, Sharda N, Giri B, et al. Melatonin and andrographolide synergize to inhibit the colospheroid phenotype by targeting Wnt/beta-catenin signaling. *J Pineal Res.* 2022;73(1):e12808.DOI:10.1111/jpi.12808

340. Quah SY, Wong CC, Wong HC, et al. Microarray-based identification of differentially expressed genes associated with andrographolide derivatives-induced resistance in colon and prostate cancer cell lines. *Toxicology & Applied Pharmacology.* 2021;425:115605.DOI:10.1016/j.taap.2021.115605

341. Liu YF, Feng ZQ, Chu TH, et al. Andrographolide sensitizes KRAS-mutant colorectal cancer cells to cetuximab by inhibiting the EGFR/AKT and PDGFRβ/AKT signaling pathways. *Phytomedicine.* 2024;126:155462.DOI:10.1016/j.phymed.2024.155462

342. Liu W, Fan T, Li M, et al. Andrographolide potentiates PD-1 blockade immunotherapy by inhibiting COX2-mediated PGE2 release. *Int Immunopharmacol.* 2020;81:106206.DOI:10.1016/j.intimp.2020.106206

343. Li X, Tian R, Liu L, et al. Andrographolide enhanced radiosensitivity by downregulating glycolysis via the inhibition of the PI3K-Akt-mTOR signaling pathway in HCT116 colorectal cancer cells. *J Int Med Res.* 2020;48(8):300060520946169.DOI:10.1177/0300060520946169

344. Fang YY, Huang JM, Wen JY, et al. AZGP1 Up-Regulation is a Potential Target for Andrographolide Reversing Radioresistance of Colorectal Cancer. *Pharmacogenomics & Personalized Medicine.* 2022;15:999-1017.DOI:10.2147/pgpm.s360147

345. Banerjee V, Sharda N, Huse J, et al. Synergistic potential of dual andrographolide and melatonin targeting of metastatic colon cancer cells: Using the Chou-Talalay combination index method. *Eur J Pharmacol.* 2021;897:173919.DOI:10.1016/j.ejphar.2021.173919

346. Xia J, Guo P, Yang J, Zhang T, Pan K, Wei H. Piperine induces autophagy of colon cancer cells: Dual modulation of AKT/mTOR signaling pathway and ROS production. *Biochemical & Biophysical Research Communications.* 2024;728:150340.DOI:10.1016/j.bbrc.2024.150340

347. Wu C, Qian Y, Jiang J, Li D, Feng L. Piperine inhibits the proliferation of colorectal adenocarcinoma by regulating ARL3-mediated endoplasmic reticulum stress. *Biomol Biomed.* 2024.DOI:10.17305/bb.2024.10525

348. Srivastava S, Dewangan J, Mishra S, et al. Piperine and Celecoxib synergistically inhibit colon cancer cell proliferation via modulating Wnt/β-catenin signaling pathway. *Phytomedicine.* 2021;84:153484.DOI:10.1016/j.phymed.2021.153484

349. Sharma S, Choudhary S, Kaur S, et al. Piperine analog PGP-41 treatment overcomes paclitaxel resistance in NCI/ADR-RES ovarian cells by inhibition of MDR1. *Chem Biol Interact.* 2023;381:110569.DOI:10.1016/j.cbi.2023.110569

350. Shaheer K, Somashekarappa HM, Lakshmanan MD. Piperine sensitizes radiation-resistant cancer cells towards radiation and promotes intrinsic pathway of apoptosis. *J Food Sci.* 2020;85(11):4070-4079.DOI:10.1111/1750-3841.15496

351. Rehman MU, Rashid S, Arafah A, et al. Piperine Regulates Nrf-2/Keap-1 Signalling and Exhibits Anticancer Effect in Experimental Colon Carcinogenesis in Wistar Rats. *Biology (Basel).* 2020;9(9).DOI:10.3390/biology9090302

352. Mohammadian M, Rostamzadeh Khameneh Z, Emamgholizadeh Minaei S, Ebrahimifar M, Esgandari K. Regulatory Effects of Apatinib in Combination with Piperine on MDM-2 Gene Expression, Glutathione Peroxidase Activity and Nitric Oxide level as Mechanisms of Cytotoxicity in Colorectal Cancer Cells. *Adv Pharm Bull.* 2022;12(2):404-409.DOI:10.34172/apb.2022.040

353. Li S, Nguyen TT, Ung TT, et al. Piperine Attenuates Lithocholic Acid-Stimulated Interleukin-8 by Suppressing Src/EGFR and Reactive Oxygen Species in Human Colorectal Cancer Cells. *Antioxidants (Basel).* 2022;11(3).DOI:10.3390/antiox11030530

354. Kirubhanand C, Selvaraj J, Rekha UV, et al. Molecular docking data of piperine with Bax, Caspase 3, Cox 2 and Caspase 9. *Bioinformation.* 2020;16(6):458-461.DOI:10.6026/97320630016458

355. de Almeida GC, Oliveira LFS, Predes D, et al. Piperine suppresses the Wnt/β-catenin pathway and has anti-cancer effects on colorectal cancer cells. *Sci Rep.* 2020;10(1):11681.DOI:10.1038/s41598-020-68574-2

356. Song L, Wang Y, Zhen Y, et al. Piperine inhibits colorectal cancer migration and invasion by regulating STAT3/Snail-mediated epithelial-mesenchymal transition. *Biotechnol Lett.* 2020;42(10):2049-2058.DOI:10.1007/s10529-020-02923-z

357. Wu H, Cui M, Li C, et al. Kaempferol Reverses Aerobic Glycolysis via miR-339-5p-Mediated PKM Alternative Splicing in Colon Cancer Cells. *Journal of Agricultural & Food Chemistry.* 2021;69(10):3060-3068.DOI:10.1021/acs.jafc.0c07640

358. Wu H, Du J, Li C, Li H, Guo H, Li Z. Kaempferol Can Reverse the 5-Fu Resistance of Colorectal Cancer Cells by Inhibiting PKM2-Mediated Glycolysis. *Int J Mol Sci.* 2022;23(7).DOI:10.3390/ijms23073544

359. Wang H, Quan J, Deng Y, Chen J, Zhang K, Qu Z. Utilizing network pharmacological analysis to investigate the key targets and mechanisms of kaempferol against oxaliplatin-induced neurotoxicity. *Toxicology Mechanisms & Methods.* 2023;33(1):38-46.DOI:10.1080/15376516.2022.2069531

360. Sharma A, Sinha S, Rathaur P, et al. Reckoning apigenin and kaempferol as a potential multi-targeted inhibitor of EGFR/HER2-MEK pathway of metastatic colorectal cancer identified using rigorous computational workflow. *Mol Divers.* 2022;26(6):3337-3356.DOI:10.1007/s11030-022-10396-7

361. Sharma A, Chorawala MR, Rawal RM, Shrivastava N. Integrated blood and organ profile analysis to evaluate ameliorative effects of kaempferol on 5-fluorouracil-induced toxicity. *Sci Rep.* 2024;14(1):2363.DOI:10.1038/s41598-024-52915-6

362. Pu Y, Han Y, Ouyang Y, et al. Kaempferol inhibits colorectal cancer metastasis through circ_0000345 mediated JMJD2C/β-catenin signalling pathway. *Phytomedicine.* 2024;128:155261.DOI:10.1016/j.phymed.2023.155261

363. Priyamvada P, Ashok G, Joshi T, Anbarasu S, Anbarasu A, Ramaiah S. Unravelling the molecular mechanistic pathway underlying the anticancer effects of kaempferol in colorectal cancer: a reverse pharmacology network approach. *Mol Divers.* 2024.DOI:10.1007/s11030-024-10890-0

364. Park J, Lee GE, An HJ, et al. Kaempferol sensitizes cell proliferation inhibition in oxaliplatin-resistant colon cancer cells. *Arch Pharm Res.* 2021;44(12):1091-1108.DOI:10.1007/s12272-021-01358-y

365. Li X, Khan I, Huang G, et al. Kaempferol acts on bile acid signaling and gut microbiota to attenuate the tumor burden in ApcMin/+ mice. *Eur J Pharmacol.* 2022;918:174773.DOI:10.1016/j.ejphar.2022.174773

366. Hassan ESG, Hassanein NM, Sayed Ahmed HM. Probing the chemoprevention potential of the antidepressant fluoxetine combined with epigallocatechin gallate or kaempferol in rats with induced early stage colon carcinogenesis. *J Pharmacol Sci.* 2021;145(1):29-41.DOI:10.1016/j.jphs.2020.10.005

367. Gu C, Tang L, Hao Y, et al. Network pharmacology and bioinformatics were used to construct a prognostic model and immunoassay of core target genes in the combination of quercetin and kaempferol in the treatment of colorectal cancer. *J Cancer.* 2023;14(11):1956-1980.DOI:10.7150/jca.85517

368. Xing F, Wang Z, Bahadar N, Wang C, Wang XD. Molecular insights into kaempferol derivatives as potential inhibitors for CDK2 in colon cancer: pharmacophore modeling, docking, and dynamic analysis. *Front Chem.* 2024;12:1440196.DOI:10.3389/fchem.2024.1440196

369. Sougiannis AT, VanderVeen B, Chatzistamou I, et al. Emodin reduces tumor burden by diminishing M2-like macrophages in colorectal cancer. *American Journal of Physiology-Gastrointestinal and Liver Physiology.* 2022;322(3):G383-G395.DOI:10.1152/ajpgi.00303.2021

370. Höhn P, Braumann C, Freiburger M, Koplin G, Dubiel W, Luu AM. Anti-tumorigenic Effects of Emodin and Its' Homologue BTB14431 on Vascularized Colonic Cancer in a Rat Model. *Asian Pac J Cancer Prev.* 2020;21(1):205-210.DOI:10.31557/apjcp.2020.21.1.205

371. Li T, Si W, Zhu J, Yin L, Zhong C. Emodin reverses 5-Fu resistance in human colorectal cancer via downregulation of PI3K/Akt signaling pathway. *Am J Transl Res.* 2020;12(5):1851-1861

372. Tang W, Hong L, Dai W, et al. MicroRNA‑500a‑5p inhibits colorectal cancer cell invasion and epithelial‑mesenchymal transition. *Int J Oncol.* 2020;56(6):1499-1508.DOI:10.3892/ijo.2020.5015

373. Zhang Y, Pu W, Bousquenaud M, et al. Emodin Inhibits Inflammation, Carcinogenesis, and Cancer Progression in the AOM/DSS Model of Colitis-Associated Intestinal Tumorigenesis. *Front Oncol.* 2020;10:564674.DOI:10.3389/fonc.2020.564674

374. Li M, Jin S, Cao Y, Xu J, Zhu S, Li Z. Emodin regulates cell cycle of non-small lung cancer (NSCLC) cells through hyaluronan synthase 2 (HA2)-HA-CD44/receptor for hyaluronic acid-mediated motility (RHAMM) interaction-dependent signaling pathway. *Cancer Cell Int.* 2021;21(1):19.DOI:10.1186/s12935-020-01711-z

375. Ahmad W, Ansari MA, Alsayari A, et al. In Vitro, Molecular Docking and In Silico ADME/Tox Studies of Emodin and Chrysophanol against Human Colorectal and Cervical Carcinoma. *Pharmaceuticals (Basel).* 2022;15(11).DOI:10.3390/ph15111348

376. Dai G, Wang D, Ma S, et al. ACSL4 promotes colorectal cancer and is a potential therapeutic target of emodin. *Phytomedicine.* 2022;102:154149.DOI:10.1016/j.phymed.2022.154149

377. Mohammed AE, Ameen F, Aabed K, et al. In-silico predicting as a tool to develop plant-based biomedicines and nanoparticles: Lycium shawii metabolites. *Biomedicine & Pharmacotherapy.* 2022;150:113008.DOI:10.1016/j.biopha.2022.113008

378. Shen Z, Zhao L, Yoo SA, et al. Emodin induces ferroptosis in colorectal cancer through NCOA4-mediated ferritinophagy and NF-κb pathway inactivation. *Apoptosis.* 2024;29(9-10):1810-1823.DOI:10.1007/s10495-024-01973-2

379. Zhou G, Xie RF, Li SN, et al. Synergic effects and possible mechanism of emodin and stilbene glycosides on colorectal cancer. *Phytomedicine.* 2024;132:155821.DOI:10.1016/j.phymed.2024.155821

380. Sun X, Zhou L, Wang Y, et al. Single-cell analyses reveal cannabidiol rewires tumor microenvironment via inhibiting alternative activation of macrophage and synergizes with anti-PD-1 in colon cancer. *J Pharm Anal.* 2023;13(7):726-744.DOI:10.1016/j.jpha.2023.04.013

381. Cherkasova V, Ilnytskyy Y, Kovalchuk O, Kovalchuk I. Transcriptome Analysis of Cisplatin, Cannabidiol, and Intermittent Serum Starvation Alone and in Various Combinations on Colorectal Cancer Cells. *Int J Mol Sci.* 2023;24(19).DOI:10.3390/ijms241914743

382. du Plessis J, Deroubaix A, Omar A, Penny C. A Bioinformatic Analysis Predicts That Cannabidiol Could Function as a Potential Inhibitor of the MAPK Pathway in Colorectal Cancer. *Curr Issues Mol Biol.* 2024;46(8):8600-8610.DOI:10.3390/cimb46080506

383. Feng P, Zhu L, Jie J, et al. Cannabidiol inhibits invasion and metastasis in colorectal cancer cells by reversing epithelial-mesenchymal transition through the Wnt/β-catenin signaling pathway. *Journal of Cancer Research & Clinical Oncology.* 2023;149(7):3587-3598.DOI:10.1007/s00432-022-04265-x

384. Kim NY, Mohan CD, Sethi G, Ahn KS. Cannabidiol activates MAPK pathway to induce apoptosis, paraptosis, and autophagy in colorectal cancer cells. *J Cell Biochem.* 2024;125(4):e30537.DOI:10.1002/jcb.30537

385. Kwon IS, Hwang YN, Park JH, et al. Metallothionein Family Proteins as Regulators of Zinc Ions Synergistically Enhance the Anticancer Effect of Cannabidiol in Human Colorectal Cancer Cells. *Int J Mol Sci.* 2023;24(23).DOI:10.3390/ijms242316621

386. Lee HS, Tamia G, Song HJ, Amarakoon D, Wei CI, Lee SH. Cannabidiol exerts anti-proliferative activity via a cannabinoid receptor 2-dependent mechanism in human colorectal cancer cells. *Int Immunopharmacol.* 2022;108:108865.DOI:10.1016/j.intimp.2022.108865

387. Nkune NW, Kruger CA, Abrahamse H. Synthesis of a novel nanobioconjugate for targeted photodynamic therapy of colon cancer enhanced with cannabidiol. *Oncotarget.* 2022;13:156-172.DOI:10.18632/oncotarget.28171

388. Raup-Konsavage WM, Carkaci-Salli N, Greenland K, Gearhart R, Vrana KE. Cannabidiol (CBD) Oil Does Not Display an Entourage Effect in Reducing Cancer Cell Viability in vitro. *Med Cannabis Cannabinoids.* 2020;3(2):95-102.DOI:10.1159/000510256

389. Wang F, Bashiri Dezfouli A, Multhoff G. The immunomodulatory effects of cannabidiol on Hsp70-activated NK cells and tumor target cells. *Mol Immunol.* 2024;174:1-10.DOI:10.1016/j.molimm.2024.07.008

390. Wang F, Dezfouli AB, Khosravi M, et al. Cannabidiol-induced crosstalk of apoptosis and macroautophagy in colorectal cancer cells involves p53 and Hsp70. *Cell Death Discov.* 2023;9(1):286.DOI:10.1038/s41420-023-01578-9

391. Zou W, Qian C, Zhang S, et al. Targeting the Ang2/Tie2 Axis with Tanshinone IIA Elicits Vascular Normalization in Ischemic Injury and Colon Cancer. *Oxidative Medicine & Cellular Longevity.* 2021;2021:7037786.DOI:10.1155/2021/7037786

392. Zhou X, Pan Y, Wang Y, et al. Tanshinones induce tumor cell apoptosis via directly targeting FHIT. *Sci Rep.* 2021;11(1):12217.DOI:10.1038/s41598-021-91708-z

393. Zhou L, Sui H, Wang T, et al. Tanshinone IIA reduces secretion of pro‑angiogenic factors and inhibits angiogenesis in human colorectal cancer. *Oncol Rep.* 2020;43(4):1159-1168.DOI:10.3892/or.2020.7498

394. Zhang Y, Zhao Y, Liu J, et al. Transgelin-2 Involves in the Apoptosis of Colorectal Cancer Cells Induced by Tanshinone-IIA. *Anal Cell Pathol (Amst).* 2022;2022:9358583.DOI:10.1155/2022/9358583

395. Zhang R, Wang Y, Liu D, et al. Sodium Tanshinone IIA Sulfonate as a Potent IDO1/TDO2 Dual Inhibitor Enhances Anti-PD1 Therapy for Colorectal Cancer in Mice. *Front Pharmacol.* 2022;13:870848.DOI:10.3389/fphar.2022.870848

396. Song Q, Yang L, Han Z, et al. Tanshinone IIA Inhibits Epithelial-to-Mesenchymal Transition Through Hindering β-Arrestin1 Mediated β-Catenin Signaling Pathway in Colorectal Cancer. *Front Pharmacol.* 2020;11:586616.DOI:10.3389/fphar.2020.586616

397. Qin C, Liu S, Zhou S, et al. Tanshinone IIA promotes vascular normalization and boosts Sorafenib's anti-hepatoma activity via modulating the PI3K-AKT pathway. *Front Pharmacol.* 2023;14:1189532.DOI:10.3389/fphar.2023.1189532

398. Qian J, Cao Y, Zhang J, et al. Tanshinone IIA Alleviates the Biological Characteristics of Colorectal Cancer via Activating the ROS/JNK Signaling Pathway. *Anticancer Agents Med Chem.* 2023;23(2):227-236.DOI:10.2174/1871520622666220421093430

399. Liu L, Gao H, Wen T, Gu T, Zhang S, Yuan Z. Tanshinone IIA attenuates AOM/DSS-induced colorectal tumorigenesis in mice via inhibition of intestinal inflammation. *Pharm Biol.* 2021;59(1):89-96.DOI:10.1080/13880209.2020.1865412

400. Ge T, Zhang Y. Tanshinone IIA reverses oxaliplatin resistance in colorectal cancer through microRNA-30b-5p/AVEN axis. *Open Med (Wars).* 2022;17(1):1228-1240.DOI:10.1515/med-2022-0512

401. Cao Y, Tang H, Wang G, et al. Targeting survivin with Tanshinone IIA inhibits tumor growth and overcomes chemoresistance in colorectal cancer. *Cell Death Discov.* 2023;9(1):351.DOI:10.1038/s41420-023-01622-8

402. Zhang Y, Zhang Y, Zhao Y, et al. Protection against ulcerative colitis and colorectal cancer by evodiamine via anti‑inflammatory effects. *Mol Med Rep.* 2022;25(5).DOI:10.3892/mmr.2022.12704

403. Wang M, Zhou B, Cong W, et al. Amelioration of AOM/DSS-Induced Murine Colitis-Associated Cancer by Evodiamine Intervention is Primarily Associated with Gut Microbiota-Metabolism-Inflammatory Signaling Axis. *Front Pharmacol.* 2021;12:797605.DOI:10.3389/fphar.2021.797605

404. Lu Y, Dong K, Yang M, Liu J. Network pharmacology-based strategy to investigate the bioactive ingredients and molecular mechanism of Evodia rutaecarpa in colorectal cancer. *BMC Complement Med Ther.* 2023;23(1):433.DOI:10.1186/s12906-023-04254-8

405. Zhu LQ, Zhang L, Zhang J, et al. Evodiamine inhibits high-fat diet-induced colitis-associated cancer in mice through regulating the gut microbiota. *J Integr Med.* 2021;19(1):56-65.DOI:10.1016/j.joim.2020.11.001

406. Zheng Q, Jing S, Hu L, Meng X. Evodiamine Inhibits Colorectal Cancer Growth via RTKs Mediated PI3K/AKT/p53 Signaling Pathway. *J Cancer.* 2024;15(8):2361-2372.DOI:10.7150/jca.92087

407. Zeng D, Zhou P, Jiang R, et al. Evodiamine inhibits vasculogenic mimicry in HCT116 cells by suppressing hypoxia-inducible factor 1-alpha-mediated angiogenesis. *Anticancer Drugs.* 2021;32(3):314-322.DOI:10.1097/cad.0000000000001030

408. Wang L, Fang K, Cheng J, et al. Scaffold Hopping of Natural Product Evodiamine: Discovery of a Novel Antitumor Scaffold with Excellent Potency against Colon Cancer. *J Med Chem.* 2020;63(2):696-713.DOI:10.1021/acs.jmedchem.9b01626

409. Mao M, Zheng X, Sheng Y, Chai J, Ding H. Evodiamine inhibits malignant progression of ovarian cancer cells by regulating lncRNA-NEAT1/miR-152-3p/CDK19 axis. *Chem Biol Drug Des.* 2023;102(1):101-114.DOI:10.1111/cbdd.14228

410. Li FS, Huang J, Cui MZ, et al. BMP9 mediates the anticancer activity of evodiamine through HIF‑1α/p53 in human colon cancer cells. *Oncol Rep.* 2020;43(2):415-426.DOI:10.3892/or.2019.7427

411. Liu L, Chen Y, Liu S, et al. Therapeutic potential of Pien Tze Huang in colitis-associated colorectal cancer: mechanistic insights from a mouse model. *Cancer Cell Int.* 2024;24(1):250.DOI:10.1186/s12935-024-03428-9

412. Huang B, Lu Y, Ni Z, et al. ANRIL promotes the regulation of colorectal cancer on lymphatic endothelial cells via VEGF-C and is the key target for Pien Tze Huang to inhibit cancer metastasis. *Cancer Gene Ther.* 2023;30(9):1260-1273.DOI:10.1038/s41417-023-00635-w

413. Gou H, Su H, Liu D, et al. Traditional Medicine Pien Tze Huang Suppresses Colorectal Tumorigenesis Through Restoring Gut Microbiota and Metabolites. *Gastroenterology.* 2023;165(6):1404-1419.DOI:10.1053/j.gastro.2023.08.052

414. Fu YB, Liu CF, Wang JJ, et al. Immunomodulatory Function of Pien Tze Huang in T Cell-Mediated Anti-tumor Activity against B16-F10, MC38 and Hep1-6 Tumor Models. *Chin J Integr Med.* 2024;30(4):348-358.DOI:10.1007/s11655-023-3749-2

415. Chen Q, Hong Y, Weng S, et al. Traditional Chinese Medicine Pien-Tze-Huang Inhibits Colorectal Cancer Growth and Immune Evasion by Reducing β-catenin Transcriptional Activity and PD-L1 Expression. *Front Pharmacol.* 2022;13:828440.DOI:10.3389/fphar.2022.828440

416. Chen Q, Hao H, Guo Z, et al. Pien Tze Huang (PZH) protects endothelial function in diabetic mice. *Life Sci.* 2024;349:122723.DOI:10.1016/j.lfs.2024.122723

417. Cao LJ, Liu LY, Chen YQ, et al. Pien Tze Huang Inhibits Proliferation of Colorectal Cancer Cells through Suppressing PNO1 Expression and Activating p53/p21 Signaling Pathway. *Chin J Integr Med.* 2024;30(6):515-524.DOI:10.1007/s11655-024-3709-5

418. Lai JQ, Zhao LL, Hong C, et al. Baicalein triggers ferroptosis in colorectal cancer cells via blocking the JAK2/STAT3/GPX4 axis. *Acta Pharmacol Sin.* 2024;45(8):1715-1726.DOI:10.1038/s41401-024-01258-z

419. Zeng Q, Zhang Y, Zhang W, Guo Q. Baicalein suppresses the proliferation and invasiveness of colorectal cancer cells by inhibiting Snail‑induced epithelial‑mesenchymal transition. *Mol Med Rep.* 2020;21(6):2544-2552.DOI:10.3892/mmr.2020.11051

420. Yu Q, Tang R, Mo W, Zhao L, Li L. Baicalein Enhances Radiosensitivity in Colorectal Cancer via JAK2/STAT3 Pathway Inhibition. *Chem Biol Drug Des.* 2024;104(2):e14611.DOI:10.1111/cbdd.14611

421. Wang CZ, Zhang CF, Luo Y, et al. Baicalein, an enteric microbial metabolite, suppresses gut inflammation and cancer progression in Apc(Min/+) mice. *Clin Transl Oncol.* 2020;22(7):1013-1022.DOI:10.1007/s12094-019-02225-5

422. Song G, Park WY, Jiao W, et al. Moderating AKT signaling with baicalein protects against weight loss by preventing muscle atrophy in a cachexia model caused by CT26 colon cancer. *Biochim Biophys Acta Mol Cell Res.* 2024;1871(3):119670.DOI:10.1016/j.bbamcr.2024.119670

423. Phan T, Nguyen VH, A'Lincourt Salazar M, et al. Inhibition of Autophagy Amplifies Baicalein-Induced Apoptosis in Human Colorectal Cancer. *Mol Ther Oncolytics.* 2020;19:1-7.DOI:10.1016/j.omto.2020.08.016

424. Chen M, Zhong K, Tan J, et al. Baicalein is a novel TLR4-targeting therapeutics agent that inhibits TLR4/HIF-1α/VEGF signaling pathway in colorectal cancer. *Clin Transl Med.* 2021;11(11):e564.DOI:10.1002/ctm2.564

425. Gu C, Lu H, Qian Z. Matrine reduces the secretion of exosomal circSLC7A6 from cancer-associated fibroblast to inhibit tumorigenesis of colorectal cancer by regulating CXCR5. *Biochemical & Biophysical Research Communications.* 2020;527(3):638-645.DOI:10.1016/j.bbrc.2020.04.142

426. Ren H, Wang Y, Guo Y, et al. Matrine impedes colorectal cancer proliferation and migration by downregulating endoplasmic reticulum lipid raft associated protein 1 expression. *Bioengineered.* 2022;13(4):9780-9791.DOI:10.1080/21655979.2022.2060777

427. Liu J, Guo Y, Cao J. Matrine triggers colon cancer cell apoptosis and G0/G1 cell cycle arrest via mediation of microRNA-22. *Phytother Res.* 2020;34(7):1619-1628.DOI:10.1002/ptr.6626

428. Li X, Lu Y, Wen P, et al. Matrine restrains the development of colorectal cancer through regulating the AGRN/Wnt/β-catenin pathway. *Environ Toxicol.* 2023;38(4):809-819.DOI:10.1002/tox.23730

429. Du Q, Lin Y, Ding C, Wu L, Xu Y, Feng Q. Pharmacological Activity of Matrine in Inhibiting Colon Cancer Cells VM Formation, Proliferation, and Invasion by Downregulating Claudin-9 Mediated EMT Process and MAPK Signaling Pathway. *Drug Design, Development & Therapy.* 2023;17:2787-2804.DOI:10.2147/dddt.s417077

430. Cheng Y, Yu C, Li W, He Y, Bao Y. Matrine Inhibits Proliferation, Invasion, and Migration and Induces Apoptosis of Colorectal Cancer Cells Via miR-10b/PTEN Pathway. *Cancer Biother Radiopharm.* 2022;37(10):871-881.DOI:10.1089/cbr.2020.3800

431. Chen Z, Dong Y, Yan Q, et al. Liquid chromatography-tandem mass spectrometry analysis of a ratio-optimized drug pair of Sophora flavescens Aiton and Coptis chinensis Franch and study on the mechanism of anti-colorectal cancer effect of two alkaloids thereof. *Front Oncol.* 2023;13:1198467.DOI:10.3389/fonc.2023.1198467

432. Liang L, Sun W, Wei X, et al. Oxymatrine suppresses colorectal cancer progression by inhibiting NLRP3 inflammasome activation through mitophagy induction in vitro and in vivo. *Phytother Res.* 2023;37(8):3342-3362.DOI:10.1002/ptr.7808

433. Zhong Y, Luo B, Hong M, et al. Oxymatrine induces apoptosis in non-small cell lung cancer cells by downregulating TRIM46. *Toxicon.* 2024;244:107773.DOI:10.1016/j.toxicon.2024.107773

434. Su JW, Zhou XH, Ye YX, Jiang Q. [Effects of oxymatrine and vincristine on drug resistance in HCT-8/VCR cells and its mechanism]. *Zhongguo Ying Yong Sheng Li Xue Za Zhi.* 2020;36(4):350-353.DOI:10.12047/j.cjap.5925.2020.075

435. Pan D, Zhang W, Zhang N, et al. Oxymatrine Synergistically Enhances Doxorubicin Anticancer Effects in Colorectal Cancer. *Front Pharmacol.* 2021;12:673432.DOI:10.3389/fphar.2021.673432

436. Li X, Sun J, Xu Q, et al. Oxymatrine Inhibits Colorectal Cancer Metastasis via Attenuating PKM2-Mediated Aerobic Glycolysis. *Cancer Management & Research.* 2020;12:9503-9513.DOI:10.2147/cmar.s267686

437. Hua S, Gu M, Wang Y, Ban D, Ji H. Oxymatrine reduces expression of programmed death-ligand 1 by promoting DNA demethylation in colorectal cancer cells. *Clin Transl Oncol.* 2021;23(4):750-756.DOI:10.1007/s12094-020-02464-x

438. Ahmed S, Keniry M, Anaya-Barbosa N, et al. Oxymatrine Loaded Cross-Linked PVA Nanofibrous Scaffold: Design and Characterization and Anticancer Properties. *Macromol Biosci.* 2023;23(10):e2300098.DOI:10.1002/mabi.202300098

439. Zhou B, Lu Y, Zhao Z, et al. B7-H4 expression is upregulated by PKCδ activation and contributes to PKCδ-induced cell motility in colorectal cancer. *Cancer Cell Int.* 2022;22(1):147.DOI:10.1186/s12935-022-02567-1

440. Wang Y, Zhang Z, Auyeung KK, Cho CH, Yung KK, Ko JK. Cryptotanshinone-Induced p53-Dependent Sensitization of Colon Cancer Cells to Apoptotic Drive by Regulation of Calpain and Calcium Homeostasis. *Am J Chin Med.* 2020;48(5):1179-1202.DOI:10.1142/s0192415x20500585

441. Wang L, Wang R, Wei GY, et al. Cryptotanshinone alleviates chemotherapy-induced colitis in mice with colon cancer via regulating fecal-bacteria-related lipid metabolism. *Pharmacol Res.* 2021;163:105232.DOI:10.1016/j.phrs.2020.105232

442. Vundavilli H, Datta A, Sima C, Hua J, Lopes R, Bittner M. Targeting oncogenic mutations in colorectal cancer using cryptotanshinone. *PLoS One.* 2021;16(2):e0247190.DOI:10.1371/journal.pone.0247190

443. Su YS, Kuo MZ, Kuo YT, et al. Diterpenoid anthraquinones as chemopreventive agents altered microRNA and transcriptome expressions in cancer cells. *Biomedicine & Pharmacotherapy.* 2021;136:111260.DOI:10.1016/j.biopha.2021.111260

444. Fu X, Zhao W, Li K, Zhou J, Chen X. Cryptotanshinone Inhibits the Growth of HCT116 Colorectal Cancer Cells Through Endoplasmic Reticulum Stress-Mediated Autophagy. *Front Pharmacol.* 2021;12:653232.DOI:10.3389/fphar.2021.653232

445. Chen L, Yang Q, Zhang H, et al. Cryptotanshinone prevents muscle wasting in CT26-induced cancer cachexia through inhibiting STAT3 signaling pathway. *J Ethnopharmacol.* 2020;260:113066.DOI:10.1016/j.jep.2020.113066

446. Park SM, Kim DY, Lee KH, Shin YI, Han SC, Kwon SM. Anti-Tumor Efficacy of Oleuropein-Loaded ZnO/Au Mesoporous Silica Nanoparticle in 5-FU-Resistant Colorectal Cancer Cells. *Int J Nanomedicine.* 2024;19:2675-2690.DOI:10.2147/ijn.s439392

447. Blanco E, Silva-Pilipich N, Bocanegra A, et al. Oleuropein-driven reprogramming of the myeloid cell compartment to sensitise tumours to PD-1/PD-L1 blockade strategies. *Br J Cancer.* 2024;130(5):869-879.DOI:10.1038/s41416-023-02561-y

448. Chioccioli S, Rocchetti G, Ruzzolini J, et al. Changes in Faecal Microbiota Profile and Plasma Biomarkers following the Administration of an Antioxidant Oleuropein-Rich Leaf Extract in a Rat Model Mimicking Colorectal Cancer. *Antioxidants (Basel).* 2024;13(6).DOI:10.3390/antiox13060724

449. Litewski S, Koss-Mikołajczyk I, Kusznierewicz B. Comparative Analysis of Phytochemical Profiles and Selected Biological Activities of Various Morphological Parts of Ligustrum vulgare. *Molecules.* 2024;29(2).DOI:10.3390/molecules29020399

450. Mahdavi Niyaki Z, Salehzadeh A, Peymani M, Zaefizadeh M. Exploring the Therapeutic Potential of Fe(3)O(4)@Glu-Oleuropein Nanoparticles in Targeting KRAS Pathway-Regulating lncRNAs in Colorectal Cancer Cells. *Biol Trace Elem Res.* 2024;202(7):3073-3085.DOI:10.1007/s12011-023-03892-w

451. Mehdinejad S, Peymani M, Salehzadeh A, Zaefizadeh M. Genetic insights and therapeutic potential for colorectal cancer: mutation analysis of KRAS gene and efficacy of Oleuropein-conjugated iron oxide nanoparticles. *Naunyn Schmiedebergs Arch Pharmacol.* 2024.DOI:10.1007/s00210-024-03182-9

452. Sain A, Sahu S, Naskar D. Potential of olive oil and its phenolic compounds as therapeutic intervention against colorectal cancer: a comprehensive review. *Br J Nutr.* 2022;128(7):1257-1273.DOI:10.1017/s0007114521002919

453. Zhang Z, Zhu Q, Wang S, Shi C. Epigallocatechin-3-gallate inhibits the formation of neutrophil extracellular traps and suppresses the migration and invasion of colon cancer cells by regulating STAT3/CXCL8 pathway. *Molecular & Cellular Biochemistry.* 2023;478(4):887-898.DOI:10.1007/s11010-022-04550-w

454. Zhang Z, Zhang S, Yang J, et al. Integrated transcriptomic and metabolomic analyses to characterize the anti-cancer effects of (-)-epigallocatechin-3-gallate in human colon cancer cells. *Toxicology & Applied Pharmacology.* 2020;401:115100.DOI:10.1016/j.taap.2020.115100

455. Ding F, Yang S. Epigallocatechin-3-gallate inhibits proliferation and triggers apoptosis in colon cancer via the hedgehog/phosphoinositide 3-kinase pathways. *Canadian Journal of Physiology & Pharmacology.* 2021;99(9):910-920.DOI:10.1139/cjpp-2020-0588

456. Choi C, Song HD, Son Y, et al. Epigallocatechin-3-Gallate Reduces Visceral Adiposity Partly through the Regulation of Beclin1-Dependent Autophagy in White Adipose Tissues. *Nutrients.* 2020;12(10):3072.DOI:10.3390/nu12103072

457. Kwon OS, Jung JH, Shin EA, Park JE, Park WY, Kim SH. Epigallocatechin-3-Gallate Induces Apoptosis as a TRAIL Sensitizer via Activation of Caspase 8 and Death Receptor 5 in Human Colon Cancer Cells. *Biomedicines.* 2020;8(4).DOI:10.3390/biomedicines8040084

458. Wu D, Liu Z, Wang Y, et al. Epigallocatechin-3-Gallate Alleviates High-Fat Diet-Induced Nonalcoholic Fatty Liver Disease via Inhibition of Apoptosis and Promotion of Autophagy through the ROS/MAPK Signaling Pathway. *Oxidative Medicine & Cellular Longevity.* 2021;2021:5599997.DOI:10.1155/2021/5599997

459. Zhou J, Li L, Pu Y, et al. Astragaloside IV inhibits colorectal cancer metastasis by reducing extracellular vesicles release and suppressing M2-type TAMs activation. *Heliyon.* 2024;10(10):e31450.DOI:10.1016/j.heliyon.2024.e31450

460. Wen LP, Gao SW, Chen HX, et al. Astragaloside IV Ameliorates Colonic Adenomatous Polyps Development by Orchestrating Gut Bifidobacterium and Serum Metabolome. *Am J Chin Med.* 2024;52(5):1527-1554.DOI:10.1142/s0192415x24500605

461. Liu F, Ran F, He H, Chen L. Astragaloside IV Exerts Anti-tumor Effect on Murine Colorectal Cancer by Re-educating Tumor-Associated Macrophage. *Arch Immunol Ther Exp (Warsz).* 2020;68(6):33.DOI:10.1007/s00005-020-00598-y

462. Liang J, Yang C, Li P, et al. Astragaloside IV inhibits AOM/DSS-induced colitis-associated tumorigenesis via activation of PPARγ signaling in mice. *Phytomedicine.* 2023;121:155116.DOI:10.1016/j.phymed.2023.155116

463. Kong P, Tang X, Liu F, Tang X. Astragaloside IV regulates circ_0001615 and miR-873-5p/LASP1 axis to suppress colorectal cancer cell progression. *Chem Biol Drug Des.* 2024;103(1):e14423.DOI:10.1111/cbdd.14423

464. Hashemi M, Esbati N, Rashidi M, et al. Biological landscape and nanostructural view in development and reversal of oxaliplatin resistance in colorectal cancer. *Transl Oncol.* 2024;40:101846.DOI:10.1016/j.tranon.2023.101846

465. Zhao Y, Deng L, Cao Y, et al. [Inhibitory Effect of Ginsenoside Rg3 Combined With 5-Fluorouracil on Tumor Angiogenesis and Tumor Growth of Colon Cancer in Mice: An Experimental Study]. *Sichuan Da Xue Xue Bao Yi Xue Ban.* 2024;55(1):111-117.DOI:10.12182/20240160506

466. Sun D, Zou Y, Song L, et al. A cyclodextrin-based nanoformulation achieves co-delivery of ginsenoside Rg3 and quercetin for chemo-immunotherapy in colorectal cancer. *Acta Pharm Sin B.* 2022;12(1):378-393.DOI:10.1016/j.apsb.2021.06.005

467. Liu Z, Wang D, Cao Q, Li J. The treatment efficacy of three-layered functional polymer materials as drug carrier for orthotopic colon cancer. *Drug Deliv.* 2022;29(1):2971-2983.DOI:10.1080/10717544.2022.2122633

468. Li X, Liu W, Geng C, et al. Ginsenoside Rg3 Suppresses Epithelial-Mesenchymal Transition via Downregulating Notch-Hes1 Signaling in Colon Cancer Cells. *Am J Chin Med.* 2021;49(1):217-235.DOI:10.1142/s0192415x21500129

469. Hong S, Cai W, Huang Z, et al. Ginsenoside Rg3 enhances the anticancer effect of 5‑FU in colon cancer cells via the PI3K/AKT pathway. *Oncol Rep.* 2020;44(4):1333-1342.DOI:10.3892/or.2020.7728

470. Huang JY, Hsu TW, Chen YR, Kao SH. Rosmarinic Acid Potentiates Cytotoxicity of Cisplatin against Colorectal Cancer Cells by Enhancing Apoptotic and Ferroptosis. *Life (Basel).* 2024;14(8).DOI:10.3390/life14081017

471. Yang K, Shen Z, Zou Y, Gao K. Rosmarinic acid inhibits migration, invasion, and p38/AP-1 signaling via miR-1225-5p in colorectal cancer cells. *Journal of Receptor & Signal Transduction Research.* 2021;41(3):284-293.DOI:10.1080/10799893.2020.1808674

472. Liu H, Deng R, Zhu CW, et al. Rosmarinic acid in combination with ginsenoside Rg1 suppresses colon cancer metastasis via co-inhition of COX-2 and PD1/PD-L1 signaling axis. *Acta Pharmacol Sin.* 2024;45(1):193-208.DOI:10.1038/s41401-023-01158-8

473. Jin BR, Chung KS, Hwang S, et al. Rosmarinic acid represses colitis-associated colon cancer: A pivotal involvement of the TLR4-mediated NF-κB-STAT3 axis. *Neoplasia.* 2021;23(6):561-573.DOI:10.1016/j.neo.2021.05.002

474. Ilhan N, Bektas I, Susam S, Ozercan IH. Protective effects of rosmarinic acid against azoxymethane-induced colorectal cancer in rats. *Journal of Biochemical & Molecular Toxicology.* 2022;36(2):e22961.DOI:10.1002/jbt.22961

475. Memari F, Mirzavi F, Jalili-Nik M, Afshari AR, Ghorbani A, Soukhtanloo M. Tumor-Inhibitory Effects of Zerumbone Against HT-29 Human Colorectal Cancer Cells. *Int J Toxicol.* 2022;41(5):402-411.DOI:10.1177/10915818221104417

476. Radaei Z, Zamani A, Najafi R, et al. Promising Effects of Zerumbone on the Regulation of Tumor-promoting Cytokines Induced by TNF-α-activated Fibroblasts. *Curr Med Sci.* 2020;40(6):1075-1084.DOI:10.1007/s11596-020-2289-7

477. Hwang S, Jo M, Hong JE, Park CO, Lee CG, Rhee KJ. Protective Effects of Zerumbone on Colonic Tumorigenesis in Enterotoxigenic Bacteroides fragilis (ETBF)-Colonized AOM/DSS BALB/c Mice. *Int J Mol Sci.* 2020;21(3).DOI:10.3390/ijms21030857

478. Dehghan R, Najafi R, Azizi Jalilian F, et al. A promising effect of zerumbone with improved anti-tumor-promoting inflammation activity of miR-34a in colorectal cancer cell lines. *Mol Biol Rep.* 2021;48(1):203-218.DOI:10.1007/s11033-020-06035-9

479. Cho HW, Rhee KJ, Eom YB. Zerumbone Restores Gut Microbiota Composition in ETBF Colonized AOM/DSS Mice. *Journal of Microbiology & Biotechnology.* 2020;30(11):1640-1650.DOI:10.4014/jmb.2006.06034

480. Cai R, Zhou YP, Li YH, Zhang JJ, Hu ZW. Baicalin Blocks Colon Cancer Cell Cycle and Inhibits Cell Proliferation through miR-139-3p Upregulation by Targeting CDK16. *Am J Chin Med.* 2023;51(1):189-203.DOI:10.1142/s0192415x23500118

481. Zhang W, Liu Q, Luo L, et al. Use Chou's 5-steps rule to study how Baicalin suppresses the malignant phenotypes and induces the apoptosis of colorectal cancer cells. *Archives of Biochemistry & Biophysics.* 2021;705:108919.DOI:10.1016/j.abb.2021.108919

482. Yang B, Bai H, Sa Y, Zhu P, Liu P. Inhibiting EMT, stemness and cell cycle involved in baicalin-induced growth inhibition and apoptosis in colorectal cancer cells. *J Cancer.* 2020;11(8):2303-2317.DOI:10.7150/jca.37242

483. Song L, Zhu S, Liu C, Zhang Q, Liang X. Baicalin triggers apoptosis, inhibits migration, and enhances anti-tumor immunity in colorectal cancer via TLR4/NF-κB signaling pathway. *J Food Biochem.* 2022;46(3):e13703.DOI:10.1111/jfbc.13703

484. Jafari B, Bahrami AR, Matin MM. Targeted bacteria-mediated therapy of mouse colorectal cancer using baicalin, a natural glucuronide compound, and E. coli overexpressing β-glucuronidase. *Int J Pharm.* 2023;642:123099.DOI:10.1016/j.ijpharm.2023.123099

485. Zhang Z, Chen Y, Zheng Y, et al. Quxie Capsule Alleviates Colitis-associated Colorectal Cancer Through Modulating the Gut Microbiota and Suppressing A. fumigatus-induced Aerobic Glycolysis. *Integr Cancer Ther.* 2022;21:15347354221138534.DOI:10.1177/15347354221138534

486. Zhang T, Xu Y, Sun LY, et al. Efficacy of Quxie Capsule in Metastatic Colorectal Cancer: Long-Term Survival Update of A Double-Blind, Randomized, Placebo Controlled Trial. *Chin J Integr Med.* 2022;28(11):971-974.DOI:10.1007/s11655-021-3281-1

487. Zhang S, Lian P, Huang T, Zhou J. Effect of Quxie capsule in patients with colorectal cancer: A systematic review and meta-analysis. *Medicine (Baltimore).* 2021;100(7):e24322.DOI:10.1097/md.0000000000024322

488. Sun L, Yan Y, Chen D, Yang Y. Quxie Capsule Modulating Gut Microbiome and Its Association With T cell Regulation in Patients With Metastatic Colorectal Cancer: Result From a Randomized Controlled Clinical Trial. *Integr Cancer Ther.* 2020;19:1534735420969820.DOI:10.1177/1534735420969820

489. Gu Z, Wang L, Zhai Ma J, Zhang T, Yang Y. Conventional Therapy Combined With Quxie Capsule Modulating Gut Microbiome in Metastatic Colorectal Cancer Patients With the Third or Above Line Setting: Result From an Investigator-Initiated, Open-Label, Single-Arm, Phase II Study. *Integr Cancer Ther.* 2024;23:15347354241280390.DOI:10.1177/15347354241280390

490. Shin JM, Lim E, Cho YS, Nho CW. Cancer-preventive effect of phenethyl isothiocyanate through tumor microenvironment regulation in a colorectal cancer stem cell xenograft model. *Phytomedicine.* 2021;84:153493.DOI:10.1016/j.phymed.2021.153493

491. Xiao J, Zhou N, Li Y, et al. PEITC inhibits the invasion and migration of colorectal cancer cells by blocking TGF-β-induced EMT. *Biomedicine & Pharmacotherapy.* 2020;130:110743.DOI:10.1016/j.biopha.2020.110743

492. Lai KC, Chueh FS, Ma YS, et al. Phenethyl isothiocyanate and irinotecan synergistically induce cell apoptosis in colon cancer HCT 116 cells in vitro. *Environ Toxicol.* 2024;39(1):457-469.DOI:10.1002/tox.23993

493. Bae I, Kim TG, Kim T, et al. Phenethyl Isothiocyanate-Conjugated Chitosan Oligosaccharide Nanophotosensitizers for Photodynamic Treatment of Human Cancer Cells. *Int J Mol Sci.* 2022;23(22).DOI:10.3390/ijms232213802

494. You W, Di A, Zhang L, Zhao G. Effects of wogonin on the growth and metastasis of colon cancer through the Hippo signaling pathway. *Bioengineered.* 2022;13(2):2586-2597.DOI:10.1080/21655979.2021.2019173

495. Radajewska A, Moreira H, Bęben D, et al. Combination of Irinotecan and Melatonin with the Natural Compounds Wogonin and Celastrol for Colon Cancer Treatment. *Int J Mol Sci.* 2023;24(11).DOI:10.3390/ijms24119544

496. Liu Y, Lu L, Cheng P, et al. Wogonin Inhibits Colorectal Cancer Proliferation and Epithelial Mesenchymal Transformation by Suppressing Phosphorylation in the AKT Pathway. *Am J Chin Med.* 2024;52(4):1155-1172.DOI:10.1142/s0192415x24500460

497. Li YP, Gong YT, Wang JX, et al. [Inhibitory effect of wogonin on human colorectal cancer cell SW480 based on network pharmacology]. *Zhongguo Zhong Yao Za Zhi.* 2020;45(8):1772-1778.DOI:10.19540/j.cnki.cjcmm.20191211.401

498. Liu T, Li K, Zhang Z, et al. Tetrandrine Inhibits Cancer Stem Cell Characteristics and Epithelial to Mesenchymal Transition in Triple-Negative Breast Cancer via SOD1/ROS Signaling Pathway. *Am J Chin Med.* 2023;51(2):425-444.DOI:10.1142/s0192415x23500222

499. Ling J, Li X, Wang M, et al. Novel sulfonyl-substituted tetrandrine derivatives for colon cancer treatment by inducing mitochondrial apoptosis and inhibiting PI3K/AKT/mTOR pathway. *Bioorg Chem.* 2024;143:107069.DOI:10.1016/j.bioorg.2023.107069

500. Tsai SC, Wu WC, Yang JS. Tetrandrine Inhibits Epithelial-Mesenchymal Transition in IL-6-Induced HCT116 Human Colorectal Cancer Cells. *OncoTargets & Therapy.* 2021;14:4523-4536.DOI:10.2147/ott.s324552

501. Li SY, Shang J, Mao XM, et al. Diosgenin exerts anti-tumor effects through inactivation of cAMP/PKA/CREB signaling pathway in colorectal cancer. *Eur J Pharmacol.* 2021;908:174370.DOI:10.1016/j.ejphar.2021.174370

502. Lai Z, Wang H, Tang X, Zhang L, Wang T, Cheng J. Study on the Mechanism of Diosgenin Targeting STAT3 to Inhibit Colon Cancer Proliferation and Migration. *Dis Markers.* 2022;2022:7494887.DOI:10.1155/2022/7494887

503. Mohammad-Sadeghipour M, Mahmoodi M, Noroozi Karimabad M, Mirzaei MR, Hajizadeh MR. Diosgenin and 4-Hydroxyisoleucine from Fenugreek Are Regulators of Genes Involved in Lipid Metabolism in The Human Colorectal Cancer Cell Line SW480. *Cell J.* 2021;22(4):514-522.DOI:10.22074/cellj.2021.6751

504. Mao Q, Min J, Zeng R, et al. Self-assembled traditional Chinese nanomedicine modulating tumor immunosuppressive microenvironment for colorectal cancer immunotherapy. *Theranostics.* 2022;12(14):6088-6105.DOI:10.7150/thno.72509

505. Gao X, Yao Y, Chen X, et al. Lentinan-functionalized selenium nanoparticles induce apoptosis and cell cycle arrest in human colon carcinoma HCT-116 cells. *Front Nutr.* 2022;9:987807.DOI:10.3389/fnut.2022.987807
